# High Affinity Dimeric Uracil-Based Receptor for the Recognition of Adenine Derivatives through Triplex-like Interactions

**DOI:** 10.1021/acs.joc.5c01309

**Published:** 2025-09-01

**Authors:** Stefano Volpi, Nicola Rivi, Saša Korom, Martina Neri, Wolfgang Knoll, Roberto Corradini

**Affiliations:** † Department of Chemistry, Life Sciences, and Environmental Sustainability, 9370 University of Parma, Parco Area delle Scienze 17 A, 43124 Parma, Italy; ‡ Istituto Nazionale Biostrutture e Biosistemi, INBB, Via dei Carpegna 19, 00165 Roma, Italy; § Faculty of Medicine and Dentistry, Danube Private University, Steiner Landstraße 124, 3500 Krems an der Donau, Austria

## Abstract

Adenine recognition through triplex-like interactions was explored using clamp-shaped artificial receptor **1**, bearing two uracil units and an extended naphthalene scaffold. The requisite binding mode was confirmed for 9-ethyladenine in CDCl_3_ via NMR investigations. Phase-transfer experiments demonstrated that **1** selectively binds adenosine from an aqueous mixture of four ribonucleosides.

## Graphical abstract

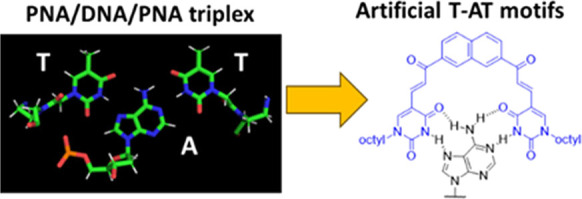

T-AT (or U-AU) and C^+^-GC triplets are the most common motifs of nucleobases that can be found in DNA (or RNA) triple helices.^1,2^ These systems consist of a central purine interacting with two complementary pyrimidines through simultaneous Hoogsteen (H) and Watson–Crick-Franklin (WCF) hydrogen bonds (Figure 1a). Similar patterns of interactions, hereafter indicated as “triplex-like interactions”, have been observed in various artificial systems, generally consisting of triple-stranded oligonucleotide structures.^1–7^ However, triplex-like interactions can also be generated in binary systems of components using synthetic nucleobases or artificial receptors that engage purine targets with both H and WCF hydrogen bonds.

**Figure 1:**
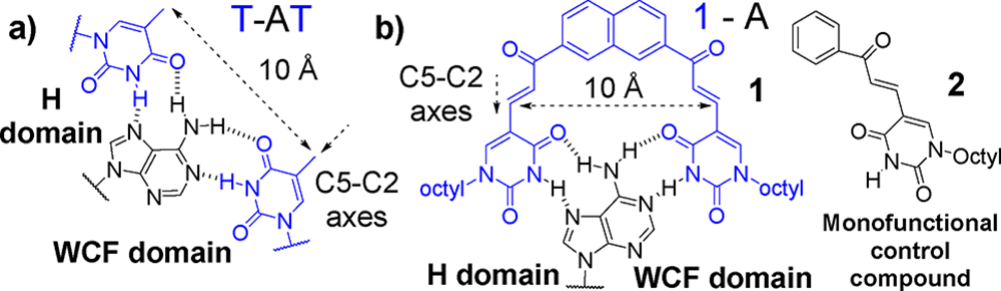
(a) Structure of natural T-AT triplets showing critical features for adenine recognition via H and WCF hydrogen bonds. (b) Artificial receptor **1** and control compound **2** used to form triplex-like motifs formed in this work.

In the case of guanine, this approach has been explored by means of the so-called “G-clamps”.^8,9^ DNA strands bearing C-to-G-clamp substitutions showed enhanced affinity and selectivity for complementary nucleic acids,^8,9^ encouraging the incorporation of these nucleobases into artificial oligonucleotides like peptide nucleic acids (PNAs)^10–12^ and the development of fluorescent probes^13–17^ and potential therapeutic agents.^18–20^ Hydrogen bonding systems have been extensively studied in supramolecular chemistry also in aqueous media,^21^ developing solid concepts for designing “artificial receptors” that target specific nucleobases. In the case of adenine, a mode of binding based on synergistic H and WCF hydrogen bonds has been reported for a class of artificial receptors named “Rebek’s clefts”, consisting of two cyclic imide units separated by an aromatic scaffold.^22–26^ However, these molecules were proposed to adopt a pocket-like conformation upon binding adenine derivatives,^23–29^ making them unsuitable to serve as artificial nucleobases. Nevertheless, enhanced adenine recognition within biological systems could enable important applications, such as the detection of oncogenic mutations involving C:G-to-T:A transitions.^30^

Accordingly, we decided to design potential nucleobases capable of using a triplex-like interaction to bind adenine residues in nucleic acid targets. Focusing on the crystal structure of a PNA-DNA-PNA triplex (PDB ID: 1 PNN),^31^ we observed that the arrangement of T-AT triplets was highly conserved, with the main geometric features being a 10 Å distance between the methyl groups of the two thymine units and a quasi-parallel orientation of their C5–C2 axes (Figure 1a). Molecular dynamics simulations involving PNA-DNA duplexes indicated that similar architectures could be assembled using planar systems like compound **1** (Figure 1b) to form triplex-like interactions with complementary adenines.^32^ Specifically, we suggested that this potential nucleobase, consisting of two uracil units and an extended, fully conjugated naphthalene scaffold, could mimic the formation of T-AT or U-AU triplets within DNA or RNA substrates (Section S1) and could be incorporated in oligonucleotide analogs or advanced sensory systems.^33,34^ Before incorporating this model into PNAs or other nucleic acid analogs, we validated the suggested mode and geometry of binding using artificial receptor **1**, which is soluble in organic solvents.

This communication describes the synthesis and binding activity of this nucleobase model, highlighting its very high affinity and selectivity for adenine derivatives and its enhanced recognition properties compared with the monofunctional control compound **2** (Figure 1b).

Artificial receptor **1** was synthesized through a convergent pathway based on the separate synthesis of its uracil and naphthalene units, which required the preparation of precursors **6a** (or **6b**, Section S2.3) and **10** (Scheme 1). The former was obtained from 2,7-dihydroxynaphthalene (**3**), initially using a six-step pathway ending with Arbuzov phosphonation (Scheme S1). This route had some drawbacks due to the competition with Perkow and halogen-elimination side reactions during the phosphonation step (Section S2.3).^35,36^ In the optimized route reported here, **3** was first activated via triflation (compound **4**) and then converted into diester **5** through Pd-catalyzed methoxycarbonylation. This intermediate was reacted with dimethyl methylphosphonate under basic conditions, leading to the formation of the bis-β-ketophosphonate **6a**. Compound **10** was obtained from 5-hydroxymethyl uracil (**7**), which was subjected to N-1 alkylation to give intermediate **8**. In this case, octyl chains were incorporated to enhance the solubility and minimize the self-aggregation of **1** in organic solvents. The same transformation could eventually allow us to introduce linker and/or active units on the pyrimidine ring, giving access to advanced variants of this artificial receptor (Section S1). Then, **8** was converted into **9** through oxidation of its 5-hydroxymethyl unit and protected on its N-3 position with a bis­(4-methoxyphenyl)­methyl (Dod) group to give **10**. The Dod protecting group was incorporated to improve the solubility of this precursor during the following manipulations. Compounds **6a** and **10** were connected using a Wittig–Horner-Emmons (WHE) reaction to give **11**, enabling the formation of α-β unsaturated spacers in the E configuration, as required for effective adenine binding. Finally, removal of the Dod protecting groups led to the formation of artificial receptor **1**.

**Scheme 1:**
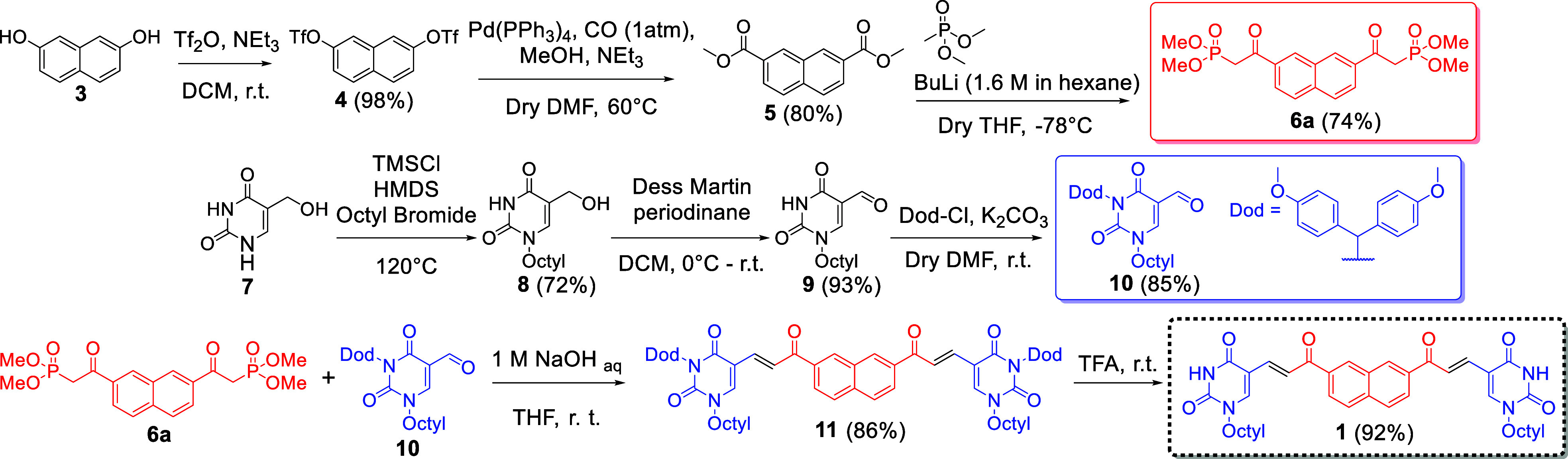
Convergent Synthesis of Artificial Receptor **1**

Similarly, the monofunctional analogue **2** was obtained by reacting methyl benzoate 13 with dimethyl methylphosphonate to give β-ketophosphonate **12**, followed by conjugation with precursor **10** under WHE conditions and removal of the Dod protecting group from compound **14** (Section S2).

The binding activity of **1** and **2** was assessed via ^1^H NMR titrations (CDCl_3_, 50 °C, Figures 2a and S6), monitoring their proton resonances in the presence of increasing amounts of 9-ethyladenine (**9-Et-A**) as a model substrate. At 25 °C, a reliable estimation of the binding constant was prevented by the exceedingly high affinity of **1** for **9-Et-A** (*K*_a_ > 10^6^ M^–1^, Figure S4c) and by the occurrence of intermediate exchange (vide infra). Instead, at 50 °C, the *K*_a_ of both **1** and **2** could be calculated, allowing comparison of the binding affinity of the dimeric receptor to that of its monomeric control compound. Working at 50 °C allowed us to measure the stability constants while remaining below the boiling point of the solvent. The concentration of both compounds was set at 0.25 mM to minimize their self-association while providing the highest attainable signal intensity, as referred to by preliminary dilution experiments (Section S3.1). The signals of **1** and **2** generally experienced gradual chemical shift perturbations (Δδ) without undergoing spectral broadening, suggesting a tendency for fast exchange between the bound and free states of the artificial receptors on the NMR time scale. The only exception was represented by the imidic resonances (*H*_im_) of **1**, which generated a broad singlet in the presence of 0.1–1.0 equiv of **9-Et-A** (Figure 2a). This effect was more pronounced at lower temperatures, leading to the disappearance of the *H*_im_ signal and to the extension of spectral broadening to the α,β unsaturated systems at 25 °C (Figure S4a,b). These observations might indicate intermediate exchange or multiple conformations adopted by **1** at low substrate concentrations (vide infra). Over the course of the titrations, the imidic resonances of **1** and **2** experienced a significant downfield shift, indicating hydrogen bond donor activity of the uracil units upon the addition of **9-Et-A** (Figures 2a and S6). However, in the case of **1**, the titrations reached their end point at lower substrate excess and caused larger chemical shift changes of *H*_im_ with respect to **2** (δΔ = 6.4 ppm at 15 equiv of **9-Et-A** vs δΔ = 4.1 ppm at 150 equiv of **9-Et-A**, Figures 2a and S6, respectively), suggesting that the dimeric artificial receptor experiences stronger interactions than its monofunctional control compound.

**Figure 2:**
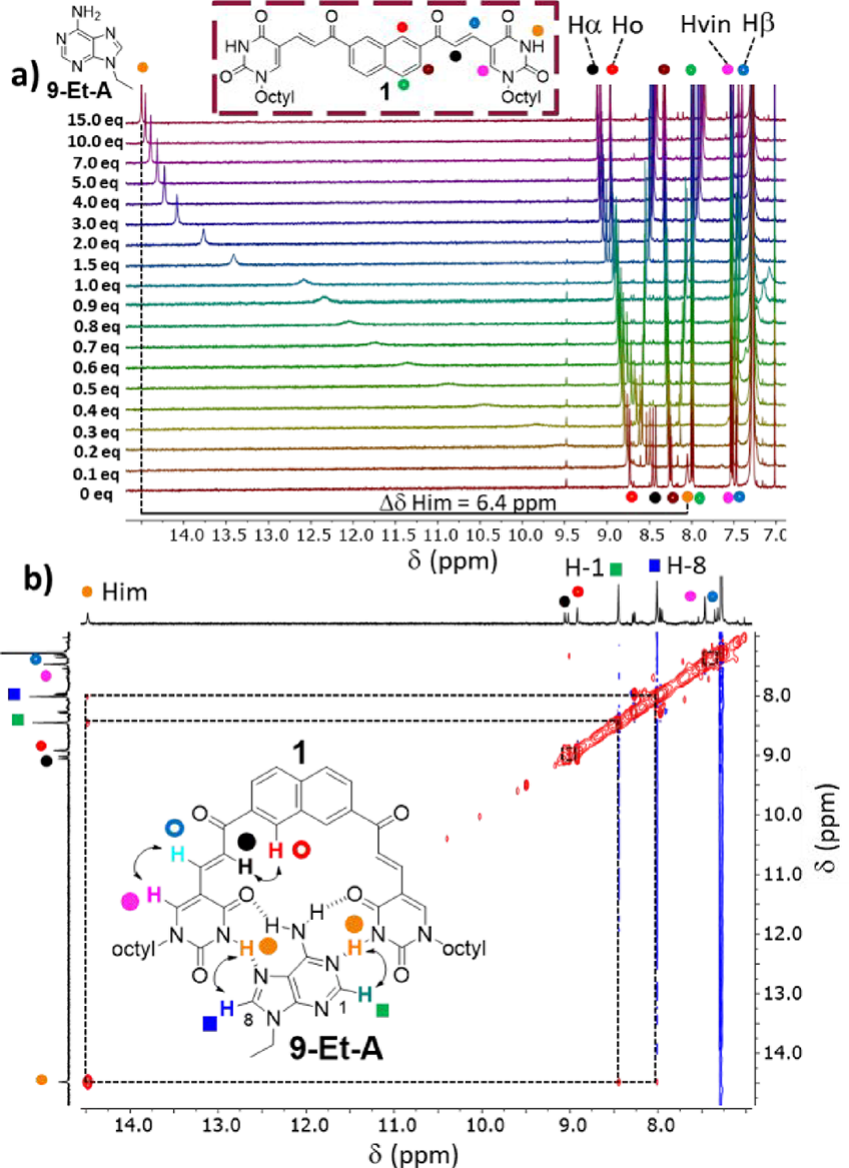
(a) ^1^H NMR titrations of 0.25 mM artificial receptor **1** with 0–15 equiv of **9-Et-A,** (CDCl_3_, 50 °C). (b) Region from a 2D NOESY spectrum of a 1/1 mixture of **1** and **9-Et-A** (CDCl_3_, 25 °C, 1.8 mM each) and proposed structure of the corresponding complex.

Substantial perturbations (δΔ ≥ 0.05 ppm) were also recorded for other four resonances of **1**, belonging to the protons of the α,β unsaturated systems, the naphthalene scaffold, and the pyrimidine rings (H_α_, H_β_, H_o_, H_vin_, Figure 2a). By contrast, all other signals of **2** remained unaffected by the addition of **9-Et-A**. Hence, the spectral changes experienced by the scaffold of **1** should be due to structural rearrangements induced by substrate recognition, rather than to the reduction of electron density around *H*_im_. Moreover, the occurrence of complex conformational equilibria between the bound and free states of **1** might explain spectral broadening in the initial part of these titrations (vide supra).

The affinity of compounds **1** and **2** for **9-Et-A** was estimated by nonlinear regression of the experimental NMR data. In both cases, the plots of the observed δΔs against **9-Et-A** concentration yielded hyperbolic curves that were fit to a 1:1 binding model, giving association constants (*K*_a_) of (4.0 ± 0.7) × 10^4^ M^–1^ and 58 ± 2 M^–1^ for the **1/9-Et-A** and **2/9-Et-A** complexes, respectively (Figure S7). The *K*_a_ value of compound **2** was close to those calculated for other adenine-uracil systems in chloroform solution,^37–41^ suggesting distinct contributions for the recognition of the H and WCF domains of **9-Et-A** (Figure S11c). On the contrary, the *K*_a_ of **1** exceeded that of **2** by ∼690 folds, implying the onset of cooperative H and WCF interactions. The occurrence of triplex-like interactions in the **1/9-Et-A** complex was assessed by NOESY experiments (CDCl_3_, 25 °C, [**1**] = [**9-Et-A**] = 1.8 mM). 2D NOESY spectra revealed cross-peaks between the H-1 and H-8 resonances of **9-Et-A** and the *H*_im_ one of **1** (Figures 2b and S10), confirming the spatial proximity of the uracil units with both the H and WCF domains of their target.

Analogous results were obtained via selective 1D NOESY experiments, where the suppression of the H-1 and H-8 signals of **9-Et-A** was observed upon irradiation of *H*_im_ and vice versa (Figure S10b). Further analyses also revealed the spatial proximity between the H_α_–H_o_ and H_β_–H_vin_ pairs of protons (Figures 2b and S10).

Based on these studies and previous molecular modeling,^32,33^ we propose that adenine recognition constrains **1** in a pinched structure (Figures 2b and S10), gradually restricting access to more extended conformations that could be adopted through rotations of the α-β unsaturated spacers.

In the case of **2**, 2D experiments under the same conditions exhibited a single cross peak between *H*_im_ and the NH_2_ signal of **9-Et-A** (Figure S11). Although this correlation could be compatible with the formation of H and WCF hydrogen bonds, the absence of NOE effects with the H-1 and H-8 resonances of **9-Et-A** suggested that these interactions were weaker than those installed by **1**.

Additional NMR titrations were carried out to evaluate the selectivity of artificial receptor **1**. These experiments were performed under the conditions used for **9-Et-A** (CDCl_3_, 50 °C, [**1**] = 0.25 mM) but exploring 1-ethyl uracil (**1-Et-U**, Figure S8), 1-ethyl cytosine (**1-Et-C**, Figure S9), and 9-ethyl guanine (**9-Et-G**) as possible substrates.

However, while a *K*_a_ of (12 ± 1) M^–1^ was estimated for the formation of the **1**/**1-Et-U** complex (Figure S8c), the scarce solubility of **1-Et-C** and **9-Et-G** in CDCl_3_ prevented the collection of full titrations. Using an excess of the dimeric artificial receptor did not improve the solubility of these substrates, suggesting a weak interaction with **1**.

A further evaluation of the selectivity for adenine-containing substrates was achieved via phase-transfer experiments (Section S4) consisting of stirring (500 rpm, 2 h, r. t.) a 1 mM CDCl_3_ solution of **1** against an equimolar D_2_O mixture of four ribonucleosides (adenosine, cytidine, uridine, and guanosine, 1 mM each), which, unlike nucleotides such as AMP or ATP, could form neutral complexes. These experiments led to the formation of a solid at the interphase, which was identified as a 1/1 mixture of **1** and adenosine (**Ade**) via ^1^H NMR analysis in DMSO-*d*6, as inferred through peak integration and comparison with the reference spectra of these two components (Figure 3, bottom, and Figure S14b).

**Figure 3:**
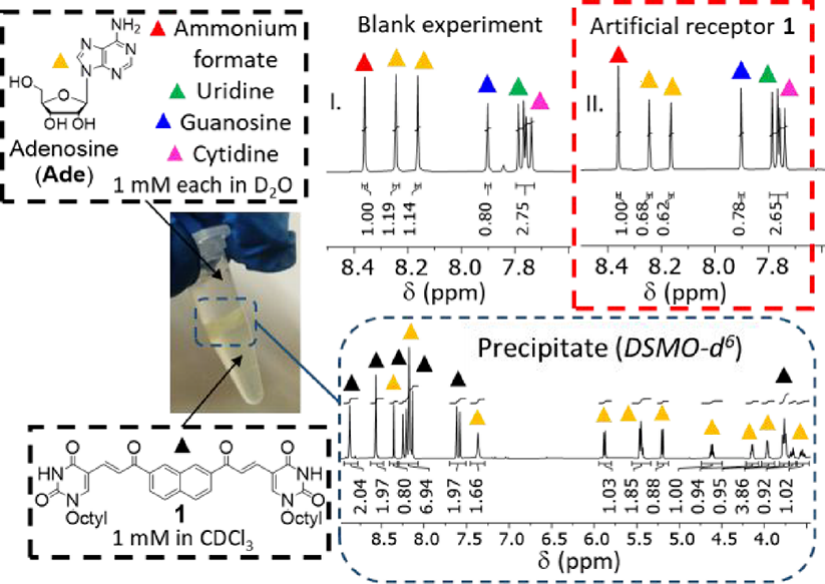
Top: ^1^H NMR spectra (D_2_O, 25 °C) of equimolar mixtures of four ribonucleosides stirred against blank CDCl_3_ (panel I) or CDCl_3_ solutions of **1** (panel II). Ammonium formate (red triangles) was used as an internal standard for peak integration. The aromatic protons of **Ade**, uridine, cytidine, and guanosine are labeled with yellow, green, magenta, and blue triangles, respectively. Bottom: ^1^H NMR spectrum (DMSO-*d* ^6^) of the solid isolated from the interphase.

The absence of signals belonging to the other three ribonucleosides provided evidence of selective adenosine binding. Solid formation was not observed using CDCl_3_ solutions of compound **2**, indicating a superior binding affinity of the dimeric artificial receptor when compared to its monofunctional control compound. Adenosine precipitation was quantified through ^1^H NMR analyses of D_2_O aliquots taken after experiments using blank CDCl_3_ and CDCl_3_ solution of **1** or **2**, estimating the relative concentration of the four ribonucleosides by using ammonium formate as an internal standard (Figures 3 and S14a, red triangles). The averaged value of **Ade** aromatic protons (yellow triangles) was 1.16 for blank experiments (Figure 3, top, panel I, and Figure S14a) and 0.65 for those using CDCl_3_ solutions of **1** (Figure 3, top, panel II, and Figure S14a), corresponding to an artificial receptor-induced precipitation of ∼44% **Ade** (**%Ade**). Conversely, the aromatic protons of cytidine, uridine, and guanosine (Figures 3, top, and S14a, magenta, green, and blue triangles, respectively) did not undergo substantial variations, indicating selective binding of **Ade** by **1**. The integrals of **Ade** aromatic protons remained unaffected upon using CDCl_3_ solutions of **2** (Figure S14a), confirming the inability of the monofunctional analogue to bind this substrate under these conditions. Similar results (**%Ade** ∼43%, no precipitation in the presence of **2**) were obtained for preliminary experiments using only **Ade** in the D_2_O layer (Section S4.1). The cooperativity of the triplex-like interactions involving **9-Et-A** and **1** (vide supra) suggests that the same mode of binding should drive adenosine recognition, explaining the complete selectivity observed in these phase-transfer experiments.

Remarkably, the hydrogen-bond-based recognition of adenine derivatives is also effective in the highly competitive aqueous environment, highlighting the importance of synergistic effects. Furthermore, the artificial receptor remained stable when stirred against excess water, showing no chemical changes in its NMR spectra. This suggests that the α,β-unsaturated system is unreactive toward weak nucleophiles such as nucleobase amino groups or water, although further analysis is needed to assess stability against stronger nucleophiles or longer reaction times.

The presented investigations demonstrated that dimeric systems based on uracil active units are suited for adenine recognition via triplex-like interactions. In fact, **1** has proven effective in forming the requisite combination of H and WCF hydrogen bonds, showing high affinity for model substrates and selectivity toward other nucleobases. The conversion of this artificial receptor into a nucleobase for PNA oligomers is underway to explore the development of PNA-based components for sensing devices^42–44^ and nanoscaled materials^45,46^ with functions deriving from enhanced adenine recognition.

## Supplementary Material



## Data Availability

The data underlying this study are available in the published article and its Supporting Information.

## Notes

General information, synthetic procedures, NMR and phase transfer protocols, additional comments and figures on NMR studies and phase transfer experiments, and NMR spectra of all compounds (PDF)

## ∥

Chemistry Department Selvita S.A., Podole 79, 30–394 Krakow, Poland
