# Supplementary material for: High Affinity Dimeric Uracil-Based Receptor for the Recognition of Adenine Derivatives through Triplex-like Interactions
Source: J Org Chem. 2025 Sep 1;90(36):12814–9. doi: 10.1021/acs.joc.5c01309 (PMC12442077; doi:10.1021/acs.joc.5c01309)
Supplement: Supplementary file 1 [file jo5c01309_si_001.pdf]

Supporting Information

High Affinity Dimeric Uracil-based Receptor for the Recognition of Adenine Derivatives through Triplex-like Interactions

Stefano Volpi,[a] Nicola Rivi,[a] Saša Korom,[a,b†] Martina Neri,[a] Wolfgang Knoll,[c] Roberto Corradini[a,b] *

[a] Department of Chemistry, Life Sciences, and Environmental Sustainability, University of Parma, Parco Area delle Scienze 17 A, 43124, Parma, Italy; [b] Istituto Nazionale Biostrutture e Biosistemi, INBB, Via dei Carpegna 19 - 00165 Roma; [c] Faculty of Medicine and Dentistry, Danube Private University, Steiner Landstraße 124, 3500 Krems an der Donau, Austria

† Present address: Chemistry Department Selvita S.A. Podole 79, 30-394 Krakow, Poland.

Table of Contents

1. Design of the artificial receptor **1** and Similar Nucleobase Analogs S3

2. Synthetic Procedures S3

2.1 Materials and reagents S3

2.2 Synthesis of the β-ketophosphonato precursors **6a** and **12** S4

2.2.1 Synthesis of 2,7-bis(trifluoromethanesulfonyloxy)naphthalene (**4**). S4

2.2.2 Synthesis of dimethyl 2,7-naphthalenedicarboxylate **5**. S4

2.2.3 Synthesis of tetramethyl (2,7-naphthoyldimethyl)bis(phosphonate) (**6a**). S5

2.2.4 Synthesis of dimethyl (benzoylmethyl)phosphonate (**12**). S5

2.3 Synthesis of the β-ketophosphonato precursor **6b** S5

2.3.1 Synthesis of 2,7-bis((trimethylsilyl)ethynyl) naphthalene (**15**). S6

2.3.2 Synthesis of 2,7-diethynylnaphthalene (**16**). S7

2.3.3 Synthesis of 2,7-diacetylnaphthalene (**17**). S7

2.3.4 Synthesis of 2,7-bis(bromoacetyl)naphthalene (**18**). S7

2.3.5 Synthesis of 2,7-bis(iodoacetyl)naphthalene (**19**). S8

2.3.6 Synthesis of tetraethyl (2,7-naphthoyldimethyl)bis(phosphonate) (**6b**). S8

2.4 Synthesis of the formyluracil precursor **10** S10

2.4.1 Synthesis of 5-hydroxymethyl-1-octyluracil (**8**)....S10
2.4.2 Synthesis of 5-formyl-1-octyluracil (**9**). S10
2.4.3 Synthesis of 5-formyl-3-Dod-1-octyluracil (**10**)....S11
2.5 Synthesis of artificial receptor **1** and control compound **2** S11
2.5.1 Synthesis of the Dod-protected dimeric receptor (**11**). S11
2.5.2 Synthesis of the dimeric receptor (**1**). S12
2.5.3 Synthesis of the Dod-protected monomeric control receptor (**14**)....S12
2.5.4 Synthesis of the monomeric control compound (**2**) S13
3. NMR investigations S13
3.1 Dilution experiments of artificial receptor **1** and compound **2** S14
3.2 NMR titrations S15
3.2.1 Titrations of artificial receptor **1** with **9-Et-A** S15
3.2.2 Titrations of control compound **2** with **9-Et-A**....S17
3.2.3 Fitting of NMR data....S18
3.2.3 Titrations of artificial receptor **1** with **1-Et-U** S19
3.2.4 Titrations of artificial receptor **1** with **1-Et-C** S20
3.3 NOESY experiments....S21
4. Nucleoside Selective Phase-Transfer experiments S23
4.1 Precipitation of adenosine....S23
4.2 Precipitation of adenosine from a mixture of four nucleosides S24
4.3 Quantification of precipitated adenosine S25
5. Additional Figures- NMR characterizations....S28
7. References....S47

1. Design of the artificial receptor 1 and Similar Nucleobase Analogs

Artificial receptor **1** was synthetized to investigate the behavior of potential nucleobases that, upon incorporation into PNA oligomers, could bind adenine targets through triplex-like interactions (**Figure S1**). These systems have a modular structure, enabling the independent modification of their naphthalene scaffold, the planar bridges, and N-attached group of the uracil units, giving access to different functions via rational modification of these building blocks. For example, artificial receptor **1** could be converted from an independent receptor to a nucleobase for oligonucleotide oligomers by changing the group at the N-1 positions of its pyrimidine rings. In fact, while these sites were alkylated with octyl chains to provide solubility and minimize self-aggregation of this receptor during the presented investigations, replacing one of them with a linker unit (e. g. deoxyribose or a carboxymethyl group) could enable the connection to a nucleotide or PNA backbone, giving a modified nucleobase. Furthermore, the geometrical features of the dimeric artificial receptor can serve to guide the design of new adenine-specific binders, based on variation of the proposed structure for improving solubility, chemical stability, and electronic properties of the various components.

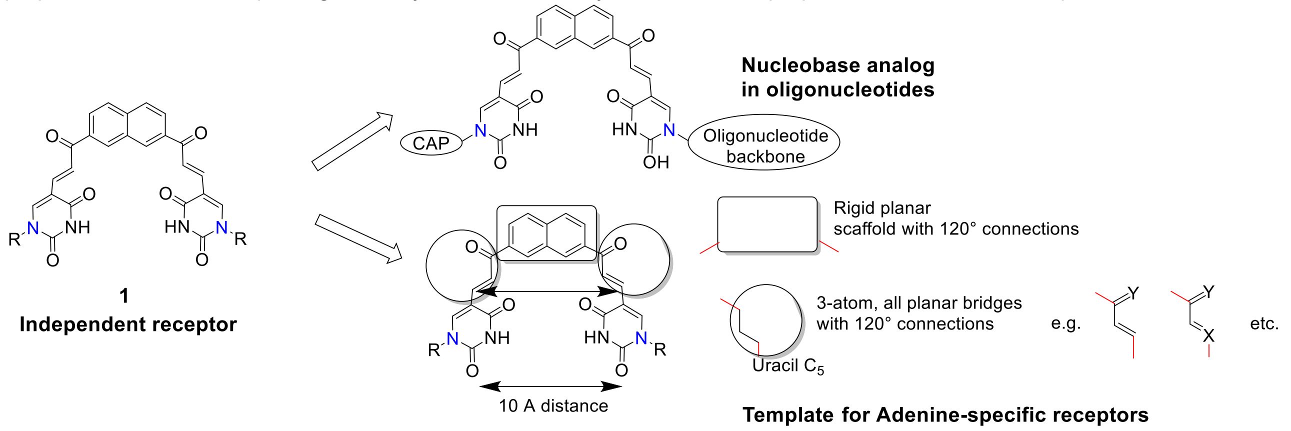

*Figure S 1: Comparison between the structure of receptor **1** and a potential nucleobase for oligonucleotide analogs and as a template for guiding the design of other receptor variants.*

The design of the artificial receptor **1** and potential daughter nucleobases was inspired by the assembly of T-AT or U-AU nucleobases triplets (**Figure 1**, **Main text**), to expand the range of systems that can bind purine derivatives through cooperative Hoogsteen and Watson-Crick-Franklin hydrogen bonds. In the following lines, we briefly summarize the main phases of this design, which was the subject of previous investigations of our group of research. [1–3] Initially, the crystal structure of a PNA-DNA-PNA triple helix (PDB ID: 1 PNN)[4] was analyzed to assess the arrangement of its T-AT triplets, revealing an averaged distance of 10 Å between the methyl groups of the thymines composing this nucleobase motif and a quasi-parallel orientation of their C5-C2 axes. Then, detailed molecular modeling showed that artificial nucleobases or receptors that mimic this architecture could be obtained by connecting the C5-positions of two uracil units to a naphthalene scaffold via α–β unsaturated spacers in E configuration.[2,3] These investigations also referred a crucial role of these spacers to modulate the conformational mobility and binding activity of these receptor or nucleobases. In fact, a more flexible version of these dimeric systems, featuring amidomethyl spacers, was previously found to induce low-to-moderate increase of affinity for DNA targets once incorporated into PNA oligomers.[2] Preliminary studies based on molecular dynamics indicated that nucleobases bearing more rigid α-β unsaturated spacers could form stable triplex-like interactions with complementary adenine residues, due to the better balance between the preorganization of their uracil units and the residual mobility of the whole system, but still lacked of experimental evidence.[3] The present study was therefore aimed at providing an experimental proof of these type of dimeric systems using the model artificial receptor **1**. This was accomplished using synthetic procedures that allowed to control the geometrical features of the designed components, such as the Wittig-Horner reaction to impart E configuration to the α–β unsaturated bridges. Different synthetic routes were tested and eventually an optimized one was identified. The results obtained allowed to demonstrate the possibility of binding simple adenine derivatives via triplex-like interactions (see the **Main text**), generating very high binding affinity and encouraging the synthesis of potential nucleobases that may translate this mode of binding to nucleic acid substrates (**Figure S1**).

2. Synthetic Procedures

2.1 Materials and reagents

9-ethyladenine (**9-Et-A**), 2,7-dihydroxynaphthalene **3**, methyl benzoate **13**, 1,3-[bis(diphenylphosphino)propane]palladium(II)chloride $PdCl_2$(dppp), and all the other commercially available reagents and solvents were bought from Merck, Carlo Erba, TCI Europe, or Fluorochem and used without further purification. 5-hydroxymethyluracil (**7**),[5] 4,4'-dimethoxydiphenylmethyl chloride (**Dod-Cl**),[6] 9-ethylguanine (**9-Et-G**),[7,8] 1-ethylcytosine (**1-Et-C**),[7] and 1-ethyluracyl (**1-Et-U**)[9] were synthesized according to literature procedures. When required, DMF was dried over 4 Å molecular sieves and purged

with nitrogen to remove dimethylamine residues, while THF was dried over 3 Å molecular sieves. $CDCl_3$ for NMR titrations was stored over 4 Å molecular sieves. TLCs were run on Merck 5554 silica 60 aluminum sheets. Flash column chromatography was performed on Merck 9385 silica gel 60 (0.040- 0.063 mm) for manual purifications, while a CombiFlash NextGen 300 system by TeleDyne ISCO and PUREZZA-Daily Standard Cartridges (silica pore size 60Å, 50 mm particle size) by Sepachrom were used for automated purifications.
NMR spectra were recorded on Bruker Avance 400 and 300 MHz instruments. δ values are expressed in ppm relative to $CDCl_3$ (7.29 ppm for ^{1}H and 76.9 ppm for ^{13}C), DMSO-*d6* (2.50 ppm for ^{1}H and 39.5 ppm for ^{13}C), or D_2O (4.76 ppm for ^{1}H). The following abbreviations are used to explain multiplicities: s=singlet, d=doublet, t=triplet, q=quartet, qui=quintuplet, dd = doublet of doublets, td = triplet of doublets m=multiplet, and br s.=broad signal. Structural assignments were made with additional information from gCOSY, gHSQC, and gHMBC experiments.
ESI-MS analyses were carried out using a Waters Acquity Ultra Performance LC instrument equipped with a Waters Acquity SQ Detector and an ESI interface. HR-MS analyses were performed on a Thermo LTQ ORBITRAP XL equipped with an ESI source.
Common reagents, solvents, and compounds have been abbreviated as follows: DMF (dimethylformamide); DCM (dichloromethane); THF (tetrahydrofuran); AcOEt (ethyl acetate); NEt_3 (triethylamine); BuLi (butyllithium); $P(OEt)_3$ (triethylphosphite); Tf_2O (trifluometahnesulfonic anhydride); TMSCl (trimethylsilyl chloride); HDMS (hexamethyldisilazane) ; PNA (peptide nucleic acid); $PdCl_2$(dppp) ([1,3-Bis(diphenylphosphino)propane]palladium(II)dichloride).

2.2 Synthesis of the β-ketophosphonato precursors 6a and 12

*2.2.1 Synthesis of 2,7-bis(trifluoromethanesulfonyloxy)naphthalene (**4**).*

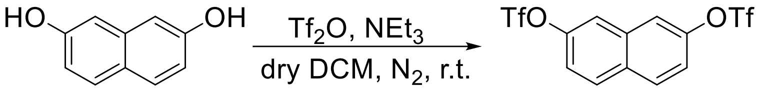

3 **4** (98%)

In an oven-dried (90°C, 1 hour) round-bottomed flask and under nitrogen atmosphere, 2,7 dihydroxynaphthalene **3** (2,3 g, 14.2 mmol) was suspended in 50 ml of dry DCM and NEt_3 (4.5 mL, 33.0 mmol) was added. After 20 minutes, Tf_2O (5.95 ml, 35.4 mmol) was added dropwise, leading to the formation of a homogeneous, brick red solution. The mixture was reacted overnight at room temperature and was quenched with distilled H_2O (50 mL) under vigorous stirring. Then, the organic layer was washed with distilled H_2O (2 x 50 ml) and brine (50 ml), dried with anhydrous Na_2SO_4, and the solvent was removed under reduced pressure. Recrystallization of the product occurred through the addition of hexane (50 mL) to the oily residue, giving compound **4** as a light brown solid (5.9 g, 13.9 mmol, 98% yield).
^{1}H-NMR (400 MHz, $CDCl_3$) δ 8.05 (d, 2H, J = 9.1 Hz, Ar-*H*-meta); 7.84 (d, 2H, J = 2.3 Hz, *Ar-H* ortho); 7.52 (dd, 2H, J_1 = 9.1 Hz, J_2 = 2.4 Hz, *Ar-H* ortho'). **Figure S15**.
The complete characterization of this compound is reported in literature.[10]

*2.2.2 Synthesis of dimethyl 2,7-naphthalenedicarboxylate **5**.*

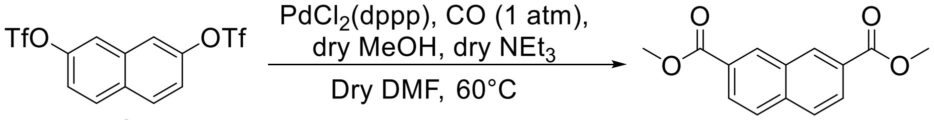

4 **5** (80%)

*Note: manual column chromatography is recommended for this crude due to the tendency of compound **5** to precipitate from the indicated eluent mixture.*

A Schlenk tube was dried in an oven (90°C, 1 hour) and provided with an inert atmosphere through three vacuum-nitrogen cycles. Then, compound **4** (2.0 g, 4.7 mmol) and $PdCl_2$(dppp) (280 mg, 0.5 mmol) were dissolved in 40 ml of dry DMF, followed by the addition of dry NEt_3 (2.6 ml, 18.9 mmol) and CH_3OH (14.3 ml, 0.4 mol). The tube was purged with CO and the resulting mixture was stirred overnight at 60°C (oil bath) under CO atmosphere (1 atm), which was provided with a ballon (Caution!). The reaction mixture was filtered through celite, and the filtrated was diluted with AcOEt and washed with 0.5 M HCl (2x50 ml) and distilled H_2O (2x50 ml). The organic layer was dried over anhydrous Na_2SO_4, and the solvent was removed under reduced pressure. The crude was purified by manual column chromatography (Hexane/AcOEt 95/5 → Hexane/AcOEt 8/2), to give compound **5** as a white solid (920 mg, 3.8 mmol, 80% yield).
^{1}H-NMR (400 MHz, $CDCl_3$) δ 8.73 (s, 2H, *Ar-H* ortho); 8.20 (dd, 2H, J_1 = 8.8 Hz, J_2 = 1.6 Hz, *Ar-H* ortho'); 7.96 (d, 2H, J = 8.8 Hz, *Ar-H* meta); 4.03 (s, 6H, OCH_3). **Figure S16**.

The complete characterization of this compound is reported in literature.[11]

*2.2.3 Synthesis of tetramethyl (2,7-naphthoyldimethyl)bis(phosphonate) (**6a**).*

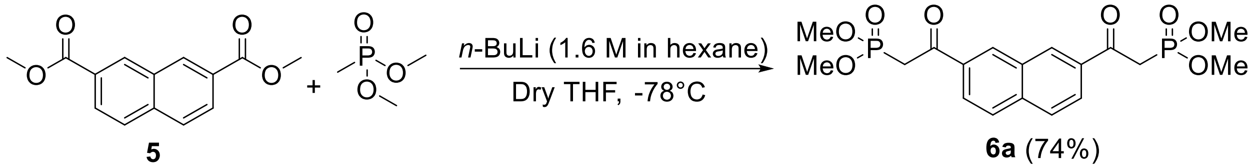

5 **6a** (74%)

Note: this reaction should be performed with freshly dried THF. The solvent should be dried overnight over 3 Å molecular sieves and stored under nitrogen atmosphere for maximum two days.

A Schlenk tube was dried in an oven (90°C, 1 hour) and provided with an inert atmosphere through three vacuum-nitrogen cycles. Then, dimethyl methylphosphonate (0.26 mL, 2.43 mmol) was dissolved in 5 mL of dry THF, the solution was cooled to -78°C (acetone - liquid nitrogen bath), and *n*-BuLi (1.6 M in hexane, 1.5 mL, 2.43 mmol) was added. After 15 minutes, compound **5** (200 mg, 0.81 mmol) was introduced in the tube, and the reaction mixture was further diluted with 4 ml of dry THF and stirred at -78°C for 2.5 hours. The reaction was quenched with 1 M HCl (10 mL) at 0°C and the resulting suspension was extracted with AcOEt, (10 mL) under vigorous stirring for 15 minutes. The aqueous layer was extracted with additional AcOEt (3x30 ml) and the organic layers were combined, dried over anhydrous Na_2SO_4, and the solvent was removed under reduced pressure. The crude was triturated in diethyl ether, giving compound **6** as a white solid (290 mg, 0.60 mmol, 74% yield). Melting Point 94 – 96 °C.
^{1}H NMR (400 MHz, $CDCl_3$) δ 8.72 (s, 2H, Ar-*H* ortho); 8.24 (dd, 2H, J_1 = 8.7 Hz, J_2 = 1.7 Hz, Ar-*H* ortho'); 8.00 (d, 2H, *J*=8.5 Hz, Ar-*H* meta); 3.86 (d, 12H, J_{HP} = 11.2 Hz, OCH_3); 3.84 (d, 4H, J_{HP} = 22.4 Hz, CH_2PO). **Figure S17**.
^{13}C{1H} NMR (101 MHz, $CDCl_3$) δ 191.3 (d, J_{CP} = 6.6 Hz); 138.1, 134.7, 133.0, 131.7, 128.6, 127.2, 53.3 (d, J_{CP} = 130.8 Hz), 38.4 (d, J_{CP} = 6.6 Hz). **Figure S18**, top.
^{31}P NMR (162 MHz, $CDCl_3$) δ 24.8. **Figure S18**, bottom.
HRMS (ESI) m/z: $[M + H]^+$ Calcd for $C_{18}H_{23}O_8P_2$ 428.0789; Found 428.0787. $[M + Na]^+$ Calcd for $C_{18}H_{22}O_8P_2Na$ 451.0682; Found 451.0679.

*2.2.4 Synthesis of dimethyl (benzoylmethyl)phosphonate (**12**).*

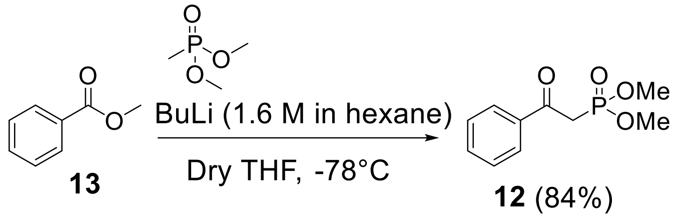

13 **12** (84%)

Note: this reaction should be performed with freshly dried THF. The solvent should be dried overnight over 3 Å molecular sieves and stored under nitrogen atmosphere for maximum two days.

A Schlenk tube was dried in an oven (90°C, 1 hour) and provided with an inert atmosphere through three vacuum-nitrogen cycles. Then, dimethyl methylphosphonate (0.9 mL, 8.14 mmol) was dissolved in 5 mL of anhydrous THF, the solution was cooled to -78°C (acetone - liquid N_2 bath), and *n*-BuLi (1.6 M in hexane, 5.1 mL, 8.14 mmol) was added. After 15 minutes, methyl benzoate (**13)** (0.79 g, 5.82 mmol) was introduced in the tube and the reaction mixture was stirred for 2 hours at -78°C. The reaction was quenched with 1 M HCl (10 mL) at 0°C and the resulting suspension was extracted with AcOEt, (10 mL) under vigorous stirring for 15 minutes. The aqueous layer was extracted with additional AcOEt (3x30 mL), and the organic layers were combined, dried over anhydrous Na_2SO_4, and the solvent was removed under reduced pressure. The crude was purified by automatic flash chromatography (AcOEt) giving compound **12** as a colorless oil (1.12 g, 4.91 mmol, 84 % yield).
^{1}H-NMR (400 MHz, $CDCl_3$) δ 8.04 (d, 2H, *J* = 7.1 Hz, Ar*H-ortho*); 7.63 (td, 1H, J_1 = 7.4 Hz, J_2 = 1.4 Hz, Ar*H-para*); 7.51 (t, 2H, J=8.1 Hz, Ar*H-meta*); 3.81 (d, 6H, J_{HP}=11.2 Hz, OCH_3); 3.66 (d, 2H, J_{HP} = 22.6 Hz, CH_2PO). **Figure S32**.
The complete characterization of this compound is reported in literature.[12]

2.3 Synthesis of the β-ketophosphonato precursor 6b

The bis-β-ketophosphonate precursor **6b**, required for the synthesis of receptor **1**, was synthetized according to the strategies reported in **Scheme S1**. 2,7-dihydroxynaphthalene **3** was converted in its bistriflated derivative **4,** which was first conjugated to

two trimethylsilyl acetylene units via Sonogashira coupling and then deprotected to give, in the order, **15** and **16**. Subsequently, iron(III)-catalyzed hydration of the two alkyne units led to the formation of the diketone **17**, which was submitted to α-bromination to give **18**. The formation of the 2,7-bis-β-ketophosphonate **6b** was initially attempted through standard Arbuzov protocols (i. e. neat $P(OEt)_3$, 160°C), giving the target product in only 37% yield (**section S2.3.6,** method A). The low yield of the process was ascribed to the interference of the Perkow reaction, which, under Arbuzov conditions, causes the conversion of α-bromoketones in the corresponding α-vinyl phosphates.[13,14] In fact, the bis-α-vinyl phosphate **20** and the β-ketophosphonate-α-vinyl phosphate **21** were isolated as major byproducts for the phosphonation of **18**. Further analyses of the crude composition also revealed the presence of the mono- and diketones **22**, **23**, and **17**, which might have formed through the hydrolysis of the α-vinyl phosphates **20** and **21** or via bromine elimination from **18**. Ketone-containing byproducts were only present in traces under these conditions.
To suppress the Perkow reaction, the literature proposes the use of α-iodoketones instead of α-bromoketones, as the lower electrophilicity and better leaving group activity of their carbonyl and iodide units, respectively, should favor the attack of $P(OEt)_3$ on their α-position.[15–17] Accordingly, the conversion of **18** in the bis-iodinated derivative **19** was examined to improve the synthesis of **6b**. The resulting sequence of reactions, including an iodination and a phosphonation step, was performed according to the following protocols (**section S2.3**): Finkelstein iodination of **18** (acetone, NaI, r.t), isolation of intermediate **19**, and subsequent submission to the Arbuzov step (toluene, $P(OEt)_3$, 80°C); Finkelstein iodination of **18** to give **19** (acetone, NaI, r.t) and immediate submission of the crude to the Arbuzov step (toluene, $P(OEt)_3$, 80°C); in-situ Finkelstein iodination of **18** before performing the Arbuzov step (acetone, NaI and then $P(OEt)_3$, r.t). These strategies also explored moderate phosphonation conditions to exploit the higher reactivity of the bis-α-iodoketone **19**, and will be hereafter indicated as methods B, C, and D, respectively. A comparison of their outcome with that of method A can be found in **Table S1** (**section S2.3.6**).

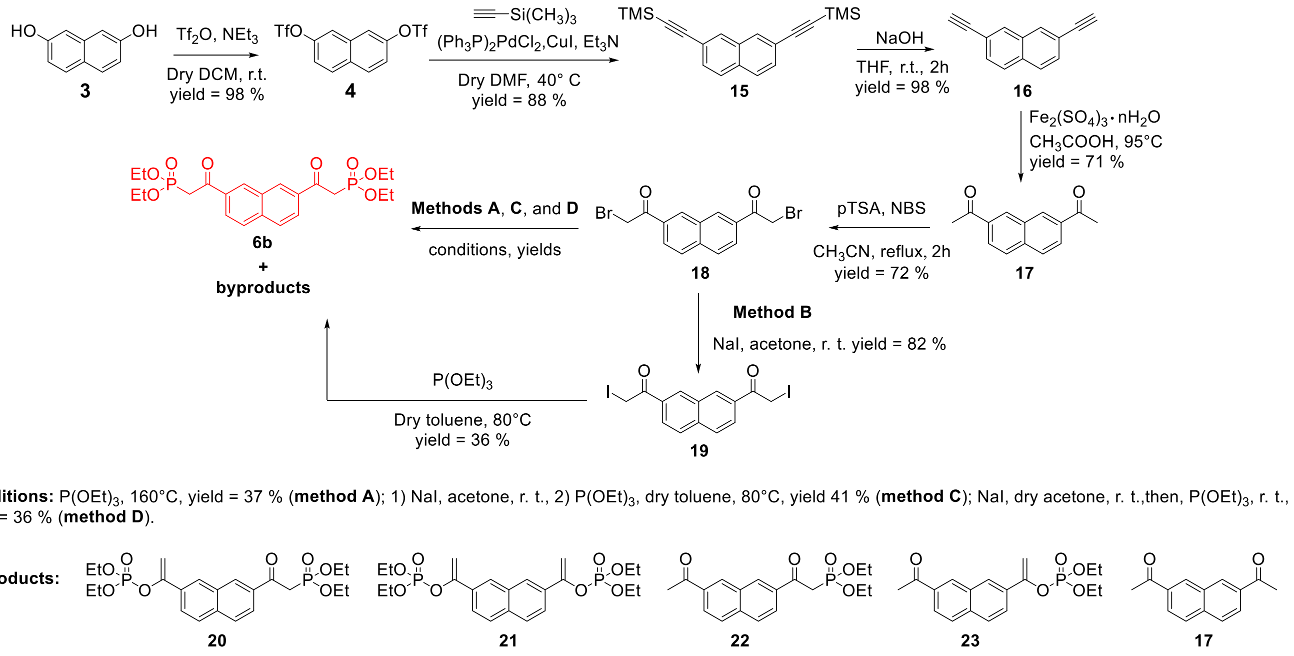

Scheme S 1. Synthesis of compound **6b**.

Compound **19** was isolated in 82% yield after the first step of method B (**section S2.3.5**), while for methods C and D the formation of this intermediate was indirectly demonstrated by the full conversion of the bis-brominated starting material. In fact, **18** did not react under the phosphonation conditions used in these protocols (i. e. moderate excess of $P(OEt)_3$, toluene, 80°C or acetone, r.t.), implying that its consumption was due to the transformation in its bis-iodinated derivative.
Regarding the phosphonation step, methods B, C, and D gave compound **21** as the only α-vinyl phosphate byproduct, with yields of 24%, 27% and 11%, respectively (**Table S1**). Variable amounts of ketone-containing byproduct were also identified (**Table S1**), with their formation being ascribable to the hydrolysis of the corresponding α-vinyl phosphates and/or the partial degradation of the α-halogenated starting materials (vide supra). The formation of dark residues during the isolation and characterization of **19** (method B, first step, **section S2.3.5**) suggested a certain tendency for this compound to degrade.
In conclusion, the use of α-haloketone intermediates to synthesize **6b** proved cumbersome due to the interference of Perkow and/or degradation side-reactions. Thus, although this route allowed to obtain an aliquot of **1** for preliminary binding studies, the synthesis of the bis-β-ketophosphonate precursor was optimized using the route presented in the main text (**Main text, Scheme 1**).[12] Precisely, the refined pathway exploited the reaction between the diester intermediate **5** and dimethyl methylphosphonate to produce **6a**. The choice of dimethyl methylphosphonate over diethyl methylphosphonate, which would have led to the formation of **6b**, was simply based on the lower cost of the former reagent.

*2.3.1 Synthesis of 2,7-bis((trimethylsilyl)ethynyl) naphthalene (**15**).*

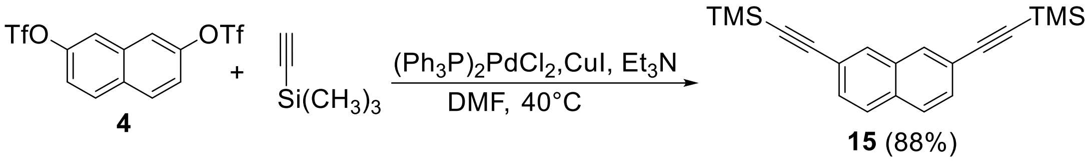

A Schlenk tube was dried in an oven (90°C, 1 hour) and provided with an inert atmosphere through three vacuum-nitrogen cycles. Then, compound **4** (2 g, 5.1 mmol), palladium(II) bis-triphenylphosphine chloride (104 mg, 0.15 mmol) and copper(I) iodide (44 mg, 0.23 mmol) were dissolved in dry DMF (20 mL), followed by the addition of ethynyltrimethylsilane (1.7 mL, 2.95 mmol) and dry triethylamine (3 mL, 5.30 mmol). The reaction was stirred overnight at 40°C (oil bath), quenched with a saturated solution of $KHSO_4$ (30 mL), and the resulting mixture was extracted with AcOEt. The combined organic layer was washed with distilled H_2O (4 x 50 ml), dried over anhydrous Na_2SO_4, and the solvent was removed under reduced pressure. The crude was taken up in hexane and filtered through celite to remove palladium residues. Compound **15** was then isolated as a beige solid (1.44 g, 4.50 mmol, 88% yield).

^1H NMR (300 MHz, $CDCl_3$) δ 7.93 (s, 2H, *ArH*-ortho), 7.75 (d, J = 8.5 Hz, 2H, *ArH*-meta), 7.53 (dd, J = 8.5, 1.0 Hz, 2H, *ArH*-ortho'), 0.31 (s, 18H, Si*CH₃*). **Figure S19**.

The complete characterization of this compound is reported in literature.[10]

*2.3.2 Synthesis of 2,7-diethynylnaphthalene (**16**).*

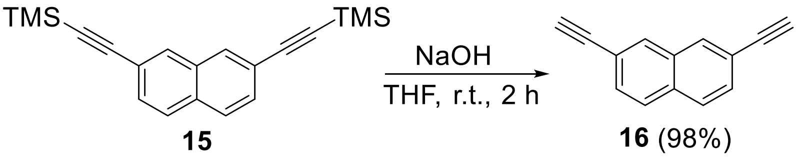

Compound **15** (734 mg, 2.29 mmol) was dissolved in THF (22 mL) and a 1 M solution of KOH (22 mL) was added. The solution was stirred for 2 h at rt, and then AcOEt (20 mL) was added to extract the product. The organic layer was dried over Na_2SO_4, and the solvent was removed under reduced pressure to give **16** as a white solid (395 mg, 2.24 mmol, 98% yield).

^1H NMR (300 MHz, $CDCl_3$) δ 8.00 (s, 2H, Ar*H*-ortho), 7.80 (d, J = 8.5 Hz, 2H, Ar*H*-meta), 7.58 (dd, J = 8.5, 1.2 Hz, 2H, Ar*H*-ortho'), 3.19 (s, 2H, C≡C*H*). **Figure S20**.

The complete characterization of this compound is reported in literature.[10]

*2.3.3 Synthesis of 2,7-diacetylnaphthalene (**17**).*

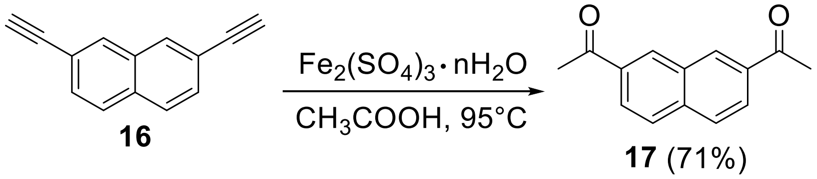

Compound **16** (600 mg, 3.40 mmol) and iron(III) sulfate hydrate (180 mg, 0.45 mmol) were dissolved in glacial acetic acid (15 mL) and the solution was heated at 95°C (oil bath) for three days. Then, the reaction was quenched with 1 M NaOH (final pH ~ 7) and the resulting mixture was extracted with AcOEt (3 x 15 mL). The combined organic layer was washed with a saturated solution of $NaHCO_3$ (3 x 30 mL), dried over Na_2SO_4, and the solvent was removed under reduced pressure. The crude was purified via automatic flash chromatography (Hex/AcOEt 8/2 → Hex/AcOEt 6/4), giving compound **17** as a beige solid (512 mg, 2.41 mmol, 71% yield).

^1H NMR (300 MHz, $CDCl_3$) δ 8.61 (s, 2H, Ar*H-ortho*), 8.19 (dd, *J* = 8.7, 1.7 Hz, 2H, Ar*H-ortho'*), 7.98 (d, *J* = 8.7 Hz, 2H, Ar*H-meta*), 2.77 (s, 6H, C*H₃*C=O). **Figure S21**.

The complete characterization of this compound is reported in literature.[18]

*2.3.4 Synthesis of 2,7-bis(bromoacetyl)naphthalene (**18**).*

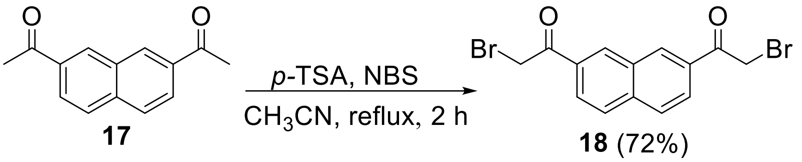

17 **18** (72%)

Note: UV detector was set at λ = 290 nm for automatic flash chromatography (toluene cut off at λ = 285 nm).

Compound **17** (219 mg, 1.03 mmol) and *p*-toluenesulfonic acid monohydrate (*p*-TSA, 533 mg, 3.10 mmol) were dissolved in acetonitrile (90 mL) and NBS was added portion wise (3 x 125 mg, 2.11 mmol). The reaction mixture was refluxed (oil bath) for 2 hours and then quenched by removing the solvent under reduced pressure. The residue was taken up in AcOEt (30 mL) and the resulting organic layer was washed with a saturated solution of $NaHCO_3$ (3 x 30 mL), dried over Na_2SO_4, and the solvent was removed under reduced pressure. The crude was purified via automatic flash chromatography (toluene → toluene/DCM 7/3), giving compound **18** as a white solid (276 mg, 0.75 mmol, 72% yield). Melting point 143-145 °C.
^{1}H NMR (400 MHz, $CDCl_3$) δ 8.68 (s, 2H, Ar*H-ortho*), 8.23 (d, *J* = 8.6 Hz, 2H, Ar*H-ortho'*), 8.03 (d, *J* = 8.6 Hz, 2H, Ar*H-meta*), 4.60 (s, 4H, C*H*$_2$Br). **Figure S22**.
^{13}C{1H} NMR (101 MHz, $CDCl_3$) δ 190.8, 138.2, 132.4, 131.7, 128.9, 127.3, 30.4. **Figure S23**.
HRMS (ESI) m/z: [M - H]$^-$ Calcd for $C_{14}H_9Br_2O_2$ 366.8975; Found 366.8968, 368.8947, 370.8926 (isotopic distribution of the two bromine atoms).

*2.3.5 Synthesis of 2,7-bis(iodoacetyl)naphthalene (**19**).*

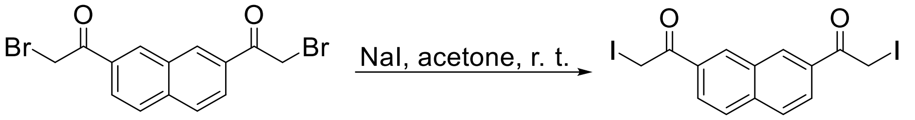

18 **19** (82%)

*Notes: The formation of dark residues in the $CDCl_3$ and MeOH solution used for the characterization of **19** suggested its tendency for degradation, which might justify the poor signal intensity of its ^{13}C NMR spectrum (**Figure S25**) and the lack of ionization in ESI HRMS analyses (see below).*

Compound **17** (33 mg, 0.090 mmol) and NaI (33 mg, 0.23 mmol) were dissolved in acetone (3 mL), and the reaction mixture was stirred in the dark and at room temperature for 30 minutes. Then, suspended NaBr was filtered off and the solvent was removed under reduced pressure. The residue was taken in an 8/2 AcOEt/acetone mixture (10 mL), and the resulting organic layer was washed with a saturated solution of $Na_2S_2O_3$ (3 x 10 mL) and dried over Na_2SO_4. Then, the solvent was removed under reduced pressure giving compound **19** as a yellow solid (34 mg, 0.073 mmol, 82% yield). Melting point not measurable; Degrades at 95 °C.
^{1}H NMR (300 MHz, $CDCl_3$) δ 8.68 (s, 2H, Ar*H-ortho*), 8.21 (dd, J_1 = 8.7 Hz, J_2 = 1.8 Hz, 2H, Ar*H-ortho'*), 8.02 (d, *J* = 8.7 Hz, 2H, Ar*H-meta*), 4.52 (s, 4H, C*H*$_2$Br). **Figure S24**.
^{13}C{1H} NMR (101 MHz, $CDCl_3$) δ 192.3, 143.4, 132.3, 131.9, 131.2, 128.8, 127.4, 1.2. **Figure S25**.
HRMS (ESI) m/z: [M + H]$^+$ Calcd for $C_{14}H_{11}I_2O_2$ 463.8770; Not found.

*2.3.6 Synthesis of tetraethyl (2,7-naphthoyldimethyl)bis(phosphonate) (**6b**).*

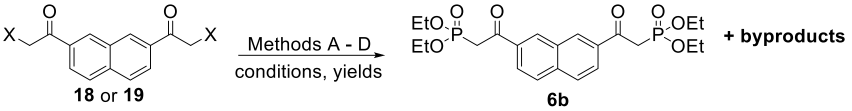

18 or **19**
Methods A, C and D: X = Br
Method B: X = I

6b

Byproducts:

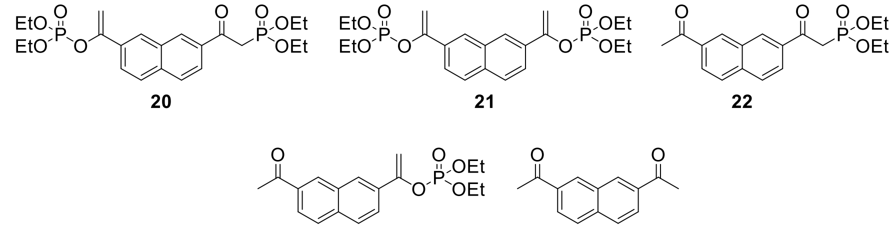

20 **21** **22** **23** **17**

Method A: Compound **18** (200 mg, 0.54 mmol) was dissolved in triethyl phosphite (3 mL, 17.5 mmol), and the resulting mixture was reacted overnight at 180°C (oil bath). Then, the reaction was quenched by removing the excess of $P(OEt)_3$ under reduced pressure. See below for crude purification.

Method B: Compound **19** (34 mg, 0.073 mmol) and $P(OEt)_3$ (40 μL, 0.23 mmol) were dissolved in dry toluene (5 mL), and the reaction mixture was stirred in the dark and at 80°C (oil bath) for 3 hours. Then, the reaction was quenched by removing the solvent and unreacted $P(OEt)_3$ under reduced pressure. The residue was taken in AcOEt, the resulting organic layer was washed with a saturated solution of $Na_2S_2O_3$ (3 x 10 mL), dried over Na_2SO_4, and evaporated under reduce pressure. See below for crude purification.

Method C: Compound **18** (50 mg, 0.14 mmol) and NaI (46 mg, 0.30 mmol) were dissolved in acetone (5 mL), and the reaction mixture was stirred in the dark and at room temperature for 30 minutes. Then, suspended NaBr was filtered off and the solvent was removed under reduced pressure. The residue was immediately taken in dry toluene (5 mL) and submitted to phosphonation through addition of $P(OEt)_3$ (140 μL, 0.81 mmol). The reaction mixture was stirred in the dark and at 80°C (oil bath) for 3 hours and quenched by removing the solvent and unreacted $P(OEt)_3$ under reduced pressure. The residue was taken in AcOEt, the resulting organic layer was washed with a saturated solution of $Na_2S_2O_3$ (3 x 10 mL), dried over Na_2SO_4, and evaporated under reduce pressure. See below for crude purification.

Method D: Compound **18** (187 mg, 0.51 mmol) and NaI (167 mg, 1.1 mmol) were dissolved in dry acetone (5 mL), and the reaction mixture was stirred in the dark and at room temperature for 30 minutes. Then, $P(OEt)_3$ (540 μL, 3.0 mmol) was added, and the resulting mixture was stirred in the dark and at room temperature for 3 hours. The reaction quenched by removing the solvent and unreacted $P(OEt)_3$ under reduced pressure. The residue was taken in AcOEt, the resulting organic layer was washed with a saturated solution of $Na_2S_2O_3$ (3 x 10 mL), dried over Na_2SO_4, and evaporated under reduce pressure. See below for crude purification.

For all methods, the crude was purified via automatic flash chromatography (3/7 acetonitrile/AcOEt → acetonitrile). Product **6b** was isolated as a white solid**,** as well as the α-vinyl phosphate byproducts **20** and **21** (the latter was only produced by method A). Ketone-containing byproducts **22**, **23**, and **17** were instead obtained as yellow oils. All the byproducts were identified by ^{1}H NMR analysis but not fully characterized.

The isolated amount and the yield of each compound are listed in **Table S1**. The ^{1}H NMR spectra of **21**, **22**, **23** and **17** revealed their co-elution with residual $P(OEt)_3$. Hence, their weighs and yields were corrected by considering the integral ratio between their OCH_2CH_3 signal and that of the contaminant.

Table S 1. Crude composition for methods A - D (n. d. = not detected).

**Protocol (scale)**	**Crude composition (mmol, yield %)**					
	**6b**	**20**	**21**	**22**	**23**	**17**
Method A (0.54 mmol)	97 mg (0.20 mmol, 37 %)	115 mg % (0.24 mmol, 45 %)	24 mg[a] (0.05 mmol, 10 %)	traces	traces	traces
Method B (0.073 mmol)	11 mg (0.022 mmol, 30 %)	8 mg (0.016 mmol, 24 %)	n. d.	4 mg[a] (0.012 mmol, 17 %)	traces	traces
Method C (0.14 mmol)	28 mg (0.057 mmol, 41 %)	18 mg (0.038 mmol, 27 %)	n. d.	5 mg[a] (0.015 mmol, 11 %)	traces	traces
Method D (0.51 mmol)	89 mg (0.18 mmol, 36 %)	27 mg (0.06 mmol, 11 %)	n. d.	45 mg[a] (0.14 mmol, 27 %)	14 mg[a] (0.04 mmol, 8%)	8 mg[a] (0.038 mmol, 7 %)

[a] Co-eluted with residual $P(OEt)_3$, corrected via ^{1}H NMR integration (vide supra).

Compound **6b**
Melting point 136 – 138 °C.
^{1}H NMR (400 MHz, $CDCl_3$) δ 8.73 (s, 2H, Ar*H-ortho*), 8.22 (dd, J_1 = 8.7 Hz, J_2 = 1.7 Hz, 2H, Ar*H-ortho'*), 7.97 (d, *J* = 8.6 Hz, 2H, Ar*H-meta*), 4.21-4.14 (m, 8H, OC*H*$_2$CH$_3$), 3.78 (d, J_{HP}= 22.8 Hz, C*H*$_2$PO), 1.30 (td, J_{HH} = 7.1 Hz, J_{HP} = 0.6 Hz, 12H, OCH$_2$C*H*$_3$). **Figure S26**.
^{13}C{1H} NMR (101 MHz, $CDCl_3$) δ 191.4 (d, J_{CP} = 6.5 Hz), 138.0, 134.8 (d, J_{CP} = 1.8 Hz), 133.0, 131.6, 128.3, 127.2, 62.8 (d, J_{CP} = 6.6 Hz), 39.41 (d, J_{CP} = 130.1 Hz), 16.32 (d, J_{CP} = 6.4 Hz). **Figure S27**, top.
^{31}P NMR (162 MHz, $CDCl_3$) δ 19.6. **Figure S27**, bottom.
HRMS (ESI) m/z: [M + H]$^+$ Calcd for $C_{22}H_{31}O_8P_2$ 485.1488; Found 485.1486. [M + Na]$^+$ Calcd for $C_{22}H_{30}O_8P_2$Na 507.1308; Found 507.1302.

Compound **20**
^{1}H NMR (300 MHz, $CDCl_3$) δ 8.56 (d, 1H, *J* = 1.8 Hz, Ar*H-ortho*), 8.20 (d, 1H, *J* = 1.7 Hz, Ar*H-ortho*), 8.06 (dd, 1H, J_1 = 8.6, J_2 = 1.8 Hz, Ar*H-ortho'*), 7.92 – 7.74 (m, 3H, Ar*H-ortho'*, Ar*H-meta*), 5.46 (m, 1H, C=C*H*$_2$), 5.38 (m, 1H, C=C*H*$_2$), 4.33 – 4.04 (m, 8H, OC*H*$_2$CH$_3$), 3.75 (d, 2H *J* = 22.7 Hz, C*H*$_2$PO), 1.37 (t, 6H, *J* = 7.1 Hz, OCH$_2$C*H*$_3$), 1.28 (t, 6H, *J* = 7.1 Hz, OCH$_2$C*H*$_3$). **Figure S28**.

Compound **21**
^{1}H NMR (400 MHz, $CDCl_3$) δ 8.09 (d, *J* = 1.8 Hz, 2H, Ar*H-ortho*), 7.80 (d, *J* = 8.8 Hz, 2H, Ar*H-meta*), 7.68 (dd, J_1 = 8.7 Hz, J_2 = 1.8 Hz, 1H, Ar*H-ortho'*), 5.44 (m, 2H, C=C*H*$_2$), 5.35 (m, 2H, C=C*H*$_2$), 4.30 - 4.22 (m, 8H, OC*H*$_2$CH$_3$), 1.39 – 1.31 (m, 12H, OCH$_2$C*H*$_3$). **Figure S29**.

Compound **22**
^{1}H NMR (400 MHz, $CDCl_3$) δ 8.62 (s, 2H, Ar*H-ortho*), 8.50 (s, 2H, Ar*H-ortho*), 8.09 (dd, 1H, J_1 = 8.7 Hz, J_2 = 1.7 Hz, Ar*H-meta*), 8.06 (dd, 1H, J_1 = 8.7 Hz, J_2 = 1.7 Hz, Ar*H-meta)*, 7.83 (dd, 2H J_1 = 8.7 Hz, J_2 = 2.3 Hz, Ar*H-ortho'*), 4.10 – 4.00 (m, 4H, OC*H*$_2$CH$_3$), 2.64 (s, 3H, C=OC*H*$_3$), 1.31 – 1.19 (m, 12H, OCH$_2$C*H*$_3$). **Figure S30**.

Compound **23**
^{1}H NMR (400 MHz, $CDCl_3$) δ 8.51 (s, 1H, Ar*H-ortho*), 8.22 (s, 1H, Ar*H-ortho*), 8.06 (dd, 1H, J_1 = 8.6 Hz, J_2 = 1.7 Hz, Ar*H-ortho'*), 7.91 (d, 1H, *J* = 8.6, Ar*H-meta*), 7.89 (d, 1H, *J* = 8.6, Ar*H-meta*), 7.80 (dd, 1H J_1 = 8.7 Hz, J_2 = 1.8 Hz, Ar*H-ortho'*), 5.49 (m, 1H, C=C*H*$_2$), 5.39 (m, 1H, C=C*H*$_2$), 4.27 – 4.22 (m, 4 H, OC*H*$_2$CH$_3$), 2.75 (s, 3H, C=OC*H*$_3$), 1.49 – 1.33 (m, 6H). **Figure S31**.

2.4 Synthesis of the formyluracil precursor 10

*2.4.1 Synthesis of 5-hydroxymethyl-1-octyluracil (**8**).*

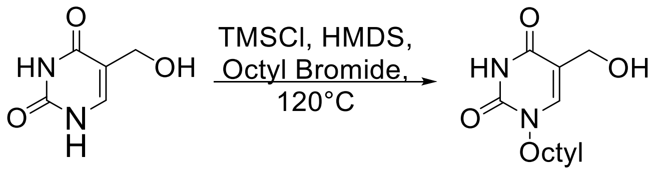

7 **8** (72%)

A Schlenk tube was dried in an oven (90°C, 1 hour) and provided with an inert atmosphere through three vacuum-nitrogen cycles. Then, compound **7** (1 g, 7.05 mmol) was suspended in HMDS (9.3 mL, 44.4 mmol), TMSCl (0.54 ml, 4.22 mmol) was added, and the mixture was heated to 120°C (oil bath). When the suspension turned into a homogeneous solution (~ 30 minutes) octyl bromide (20 mL, 0.116 mol) was added and the resulting mixture was kept at 120°C overnight. The reaction was cooled to r. t. and quenched by adding glacial acetic acid (25 mL) and distilled H_2O (25 mL) under vigorous stirring. The aqueous layer was extracted with DCM (3 x 50 ml), the combined organic layers were dried over anhydrous Na_2SO_4, and the solvent was removed under reduced pressure. The residue consisted in an orange oil, from which compound **8** was precipitated as a light brown solid (1.29 g, 5.08 mmol, 72% yield) by adding hexane. Melting point 129 -130 °C.
^{1}H-NMR (400 MHz, CDCl3, 25°C) δ 9.60 (s, 1H, N*H*); 7.24 (s, 1H, C(6)*H*); 4.43 (s, 2H, C*H*$_2$OH); 3.86 (t, 2H, *J* = 7.4 Hz, NC*H*$_2$CH$_2$); 1.75 (qui, *J* = 7.2 Hz, 2H, NCH$_2$C*H*$_2$); 1.32 (m, 10H, C*H*$_2$ chain); 0.91 (t, *J* = 6.8 Hz, 3H, CH$_2$C*H*$_3$). **Figure S33**.
^{13}C{1H} NMR (101 MHz, $CDCl_3$) δ 164.0, 150.6, 141.8, 113.5, 58.5, 49.0, 31.7, 29.1, 29.1, 26.5, 22.6, 14.1. **Figure S34**.
HRMS (ESI) m/z: [M + Na]$^+$ Calcd for $C_{13}H_{22}N_2O_3$Na 277.1523; Found 277.1510.

*2.4.2 Synthesis of 5-formyl-1-octyluracil (**9**).*

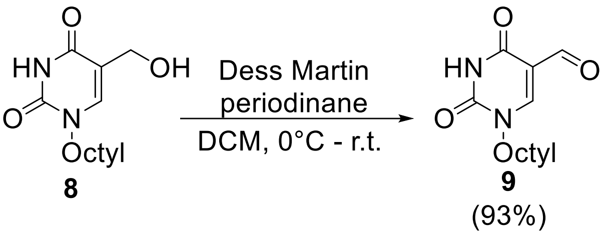

Compound **8** (1g, 3.93 mmol) was dissolved in DCM (60 mL), the solution was cooled to 0°C (ice bath), and Dess-Martin periodinane was added portion wise (2 x 1.17 g, 7.87 mmol). After 30 minutes, the reaction mixture was allowed to heat to room temperature and stirred for additional 2.5 hours. The reaction was quenched by sequentially adding a saturated solution of $NaHCO_3$ (50 mL) and a saturated solution of $Na_2S_2O_3$ (50 mL) under vigorous stirring. The organic layer was dried over anhydrous Na_2SO_4 and the solvent was removed under reduced pressure. The crude was dissolved in the minimum quantity of AcOEt and the product was recrystallized through the addition of hexane. Compound **9** was isolated as a white solid (0.91 g, 3.61 mmol, 93% yield).

^{1}H NMR (400 MHz, $CDCl_3$) δ 10.03 (s, 1H, C*H*O), 8.84 (s, 1H, N*H*), 8.10 (s, 1H, C(6)*H*), 3.86 (t, *J* = 7.6 Hz, 2H, NC*H₂*), 1.81 – 1.71 (m, 2H, NCH_2CH_2), 1.38 – 1.26 (m, 10H, CH_2 chain), 0.90 (t, *J* = 6.8 Hz, 3H, CH_2CH_3). **Figure S35**.

^{13}C NMR (101 MHz, $CDCl_3$) δ 186.1, 161.9, 149.6, 149.2, 111.0, 50.2, 31.7, 29.1, 29.0, 29.0, 26.3, 22.6, 14.0. **Figure S36**.

HRMS (ESI) m/z: $[M + H]^+$ Calcd for $C_{13}H_{21}N_2O_3$ 253.1552; Found 253.1546, $[M + Na]^+$ Calcd for $C_{13}H_{20}N_2O_3Na$ 275.1372; Found 275.1367.

The complete characterization of this compound is reported in the literature.[19]

*2.4.3 Synthesis of 5-formyl-3-Dod-1-octyluracil (**10**).*

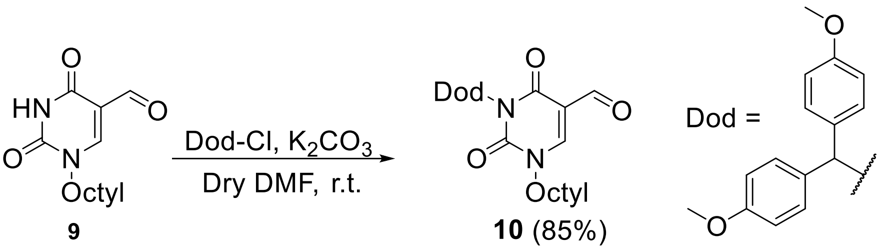

*Note: **Dod-Cl** have the same retention factor of **10** on silica, and it was used in sub-stoichiometric amount to facilitate product purification.*

A Schlenk tube was dried in an oven (90°C, 1 hour) and provided with an inert atmosphere through three vacuum-nitrogen cycles. Then, compound **9** (586 mg, 2.32 mmol) was dissolved in dry DMF (15 mL), K_2CO_3 (642 mg, 4.64 mmol) was added, and the solution was stirred at room temperature for 2 hours, during which its color first turned from light yellow to blue and then from blue to white. Subsequently, **Dod-Cl** (549 mg, 2.09 mmol) was added, and the reaction mixture was stirred overnight at room temperature and then quenched with distilled H_2O (20 mL). The resulting suspension was extracted AcOEt (3 x 30 mL), and the combined organic layers were washed with distilled H_2O (3 x 50 mL), dried over anhydrous Na_2SO_4, and the solvent was removed under reduced pressure. The residue consisted in an orange oil and was submitted to automatic flash chromatography (Hex/AcOEt 7/3), giving compound **10** as a colorless oil (955 mg, 2.0 mmol, 86% yield).

^{1}H NMR (400 MHz, $CDCl_3$) δ 10.05 (s, 1H, C*H*O), 8.06 (s, 1H, C*H*), 4H, 7.34 (d, *J* = 8.6 Hz, Ar*H-meta* Dod), 7.32 (s, 1H, Ar-C*H*-Ar Dod), 6.89 (d, 4H, *J* = 8.8 Hz, Ar*H-ortho* Dod), 3.81 – 3.79 (m, 8H, OC*H₃* Dod, NC*H₂*), 1.74-1.68 (m, 2H, NCH_2CH_2), 1.29 (m, 10H, CH_2 chain), 0.90 (t, 3H, *J* = 6.9 Hz, CH_2CH_3). **Figure S37**.

^{13}C{1H} NMR (101 MHz, $CDCl_3$) δ 187.1, 162.1, 159.0, 147.1, 130.0, 129.9, 127.7, 113.9, 113.7, 110.4, 55.2, 50.9, 31.6, 29.1, 29.0, 29.0, 26.3, 22.6, 14.0. **Figure S38**.

HRMS (ESI) m/z: $[M + Na]^+$ Calcd for $C_{56}H_{68}N_4O_{10}$ Na 979.4828; Found 979.4838.

2.5 Synthesis of artificial receptor 1 and control compound 2

*2.5.1 Synthesis of the Dod-protected dimeric receptor (**11**).*

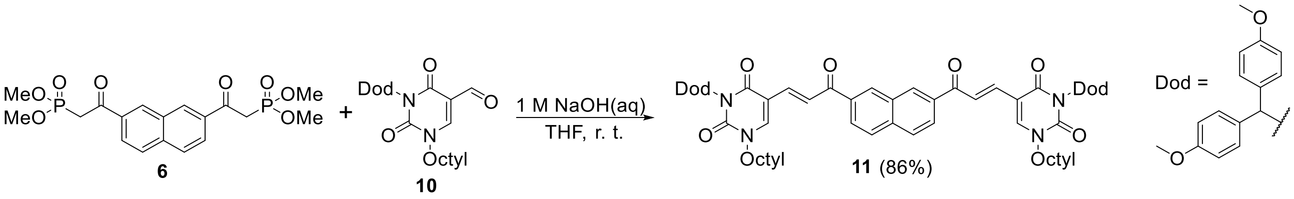

Compound **6** (35 mg, 0.072 mmol) and **10** (93 mg, 0.20 mmol) were dissolved in 10 mL of THF and an aqueous solution of NaOH (1 M, 720 µL, 0.72 mmol) was added. The THF layer readily turned orange, and the resulting biphasic mixture was left under vigorous stirring at room temperature for 2 hours. Then, the reaction mixture was diluted with brine (10 mL), giving an aqueous suspension that was extracted with AcOEt (3 x 15 mL). The collected organic layers were dried over anhydrous Na_2SO_4, and the solvent was removed under reduced pressure. Flash chromatography was performed in two-steps: after dry loading, the crude was adsorbed on silica (9/1 DCM/Hex, circa 1.5 dead volumes) and then eluted with a suitable solvent mixture (Hex/AcOEt 6.5/3.5). Compound **11** was isolated as a yellow solid (70 mg, 0.062 mmol, 86% yield). Melting point 188 -190 °C.

^{1}H NMR (400 MHz, $CDCl_3$) δ 8.75 (s, 2H, C*H-ortho* napthalene), 8.46 (d, *J* = 15.2 Hz, 2H, C=OC*H*=CH), 8.25 (dd, 2H J_1 = 8.6, J_2 1.2 Hz, Ar*H-ortho'* napthalene), 7.97 (d, 2H, *J* = 8.6 Hz, Ar*H-meta* napthalene), 7.58 (s, 2H, C(6)*H* uracil), 7.56 (d, 2H, C=OCH=C*H*), 7.45 (s, 2H, Ar-C*H*-Ar Dod), 7.36 (d, 8H, *J* = 8.7 Hz, Ar*H-meta* Dod), 6.89 (d, 8H, *J* = 8.8 Hz, Ar*H-ortho* Dod), 3.83 – 3.80 (m, 16H, NCH_2, OCH_3 Dod), 1.72 (qui, 4H, *J* = 6.4 Hz, NCH_2CH_2), 1.31 – 1.28 (m, 20H, CH_2 chains), 0.92 (t, 6H, *J* = 6.8 Hz, CH_2CH_3). **Figure S39**.

^{13}C{1H} NMR (101 MHz, $CDCl_3$) δ 190.0, 161.5, 158.8, 146.4, 137.5, 137.2, 136.2, 131.6, 130.4, 129.9, 128.4, 127.0, 122.2, 113.6, 55.2, 53.8, 50.4, 31.7, 29.7, 29.3, 29.2, 29.1, 26.4, 22.6, 14.1. **Figure S40**.

HRMS (ESI) m/z: $[M + Na]^+$ Calcd for $C_{70}H_{76}N_4O_{10}Na$ 1155.5454; Found 1155.5464.

*2.5.2 Synthesis of the dimeric receptor (**1**).*

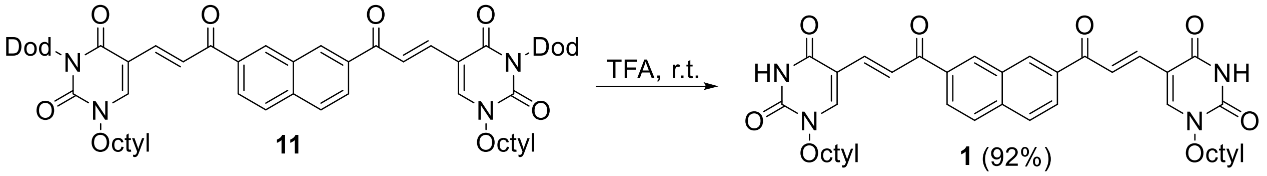

Compound **11** (100 mg, 0.096 mmol) was dissolved in TFA (4mL, 52 mmol) and the reaction mixture, which turned bright red, was stirred at room temperature for 2 hours. Then, TFA was removed under reduced pressure and the crude was triturated in CH_3CN at room temperature. The suspended solid was filtered and washed with cold diethyl ether. Compound **1** was isolated as a yellow solid (60 mg, 0.088 mmol, 92% yield). Melting point 102 - 104 °C.

^{1}H NMR (400 MHz, $CDCl_3$) δ 8.74 (s, 2H, Ar*H-ortho* napthalene), 8.49 (d, 2H, *J* = 15.2 Hz, 2H, C=OC*H*=CH), 8.42 (s, 2H, N*H*), 8.25 (d, 2H, *J* = 8.6 Hz, Ar*H-meta* napthalene), 7.99 (d, 2H, *J* = 8.6 Hz, Ar*H-ortho'* napthalene), 7.55 (s, 2H, C(6)*H* uracil), 7.51 (d, 2h, *J* = 15.2 Hz, C=OCH=C*H*), 3.87 (t, 4H, *J* = 7.3 Hz, NCH_2), 1.81-1.74 (m, 4H, NCH_2CH_2), 1.33 (m, 20H, CH_2 chains), 0.92 (t, 6H, *J* = 6.8 Hz, CH_2CH_3). **Figure S41**.

^{13}C{1H} NMR (101 MHz, $CDCl_3$) δ 189.8, 161.2, 149.2, 147.7, 136.1, 136.1, 131.8, 128.4, 127.0, 122.9, 110.3, 49.6, 31.7, 29.2, 29.1, 29.1, 26.4, 22.6, 14.1. **Figure S42**.

HRMS (ESI) m/z: $[M + H]^+$ Calcd for $C_{40}H_{49}N_4O_6$ 680.3574; Found 680.3597. $[M + Na]^+$ Calcd for $C_{40}H_{48}N_4O_6Na^+$ 703.3472; Found 703.3479.

*2.5.3 Synthesis of the Dod-protected monomeric control receptor (**14**)*

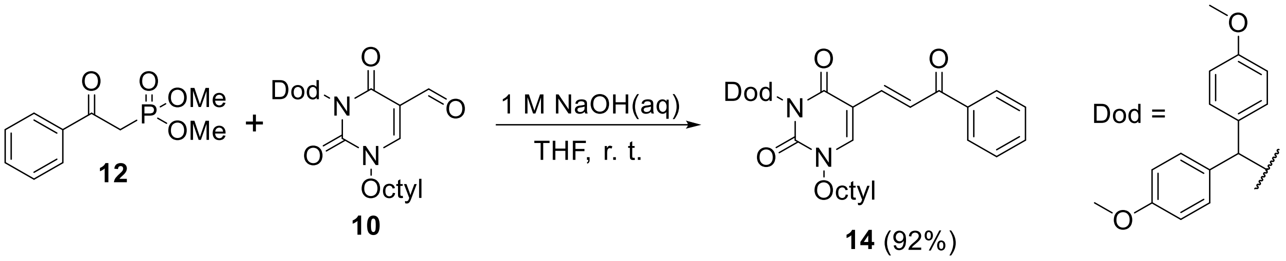

*Note: compound **10** have almost the same retention factor of **14** on silica, and it was used in sub-stoichiometric amount to facilitate product purification.*

Compounds **12** (62 mg, 0.27 mmol) and **10** (97 mg, 0.20 mmol) were dissolved in THF (2 mL) and an aqueous solution of NaOH (1 M, 2.0 mL, 2.00 mmol) was added. The resulting biphasic mixture was vigorously stirred for 2 hours, followed by the dilution of the organic and aqueous layers with ethyl acetate (10 mL) and brine (10 mL), respectively, and additional 30 minutes of vigorous stirring. Then, the organic layer was dried over anhydrous Na_2SO_4, the solvent was removed under reduced pressure, and the crude material was purified by flash chromatography (Hex/AcOEt 7/3). Compound **14** was isolated as a light green oil (110 mg, 0.19 mmol, 71% yield).

^{1}H NMR (400 MHz, $CDCl_3$) δ 8.32 (d, 1H, *J* = 15.3 Hz, C=OC*H*=CH), 8.08 (d, *J* = 7.2 Hz, 2H, Ar*H-ortho*), 7.58 (t, 1H, *J* = 7.3 Hz, Ar*H-para*), 7.52 (s, 1H, C(6)*H* uracil), 7.49 (t, 2H, *J* = 7.5 Hz, Ar*H-meta*), 7.47 (d, 1H, *J* = 15.3 Hz, C=OCH=C*H*), 7.41 (s, 1H, ArC*HAr* Dod), 7.36 (d, 4H, *J* = 8.7 Hz, Ar*H-meta* Dod), 6.90 (d, 4H *J* = 8.8 Hz, Ar*H-ortho* Dod), 3.81 – 3.77 (m, 8H, OC*H*$_3$ Dod, NC*H*$_2$), 1.71 (m, 2H, NCH_2CH_2), 1.31 – 1.23 (m, 10H, C*H*$_2$ chain), 0.92 (t, 3H, *J* = 6.9 Hz, CH_2CH_3). **Figure S43**.

^{13}C{1H} NMR (101 MHz, $CDCl_3$) δ 190.6, 161.4, 158.8, 149.7, 146.2, 138.1, 136.8, 132.8, 130.4, 129.9, 128.7, 128.6, 122.6, 113.6, 109.5, 58.7, 55.2, 50.4, 31.7, 30.9, 29.2, 29.1, 26.4, 22.6, 14.1. **Figure S44**.

HRMS (ESI) m/z: [M + Na]$^+$ Calcd for $C_{36}H_{40}N_2O_5Na$ 603.2829; Found 603.2832.

*2.5.4 Synthesis of the monomeric control compound (**2**)*

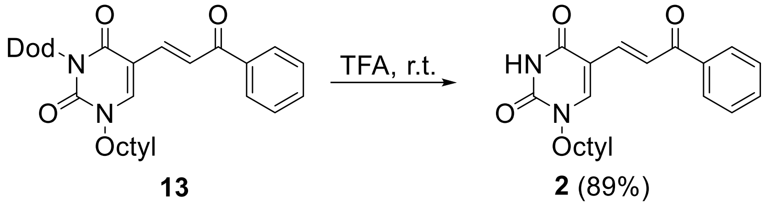

Compound **14** (59 mg, 0.10 mmol) was dissolved in TFA (2 mL, 26 mmol) and the solution was stirred at room temperature for 45 minutes. Then, the reaction was quenched with methanol (5 mL) and the volatiles were removed under reduced pressure. The crude was purified by flash chromatography (Hex/THF 6/4), giving compound **2** as a white solid (32 mg, 0.09 mmol, 89% yield). Melting Point 173 – 175 °C.

^{1}H NMR (400 MHz, $CDCl_3$) δ 8.60 (s, 1H, N*H*), 8.35 (d, 1H, *J* = 15.3 Hz, C=OC*H*=CH), 8.09 (d, 2H, *J* = 7.3 Hz, Ar*H-ortho*), 7.60 (t, 1H, *J* = 7.3 Hz, Ar*H-para*), 7.51 – 7.49 (m, 3H, Ar*H-meta*, C(6)*H* uracil), 7.44 (d, 1H, *J* = 15.3 Hz, C=OCH=C*H*), 3.85 (t, 2H *J* = 7.4 Hz, NC*H*$_2$), 1.78 – 1.74 (m, 2H, NCH_2CH_2), 1.36 (m, 10H, C*H*$_2$ chain), 0.92 (t, 3 H, *J* = 6.8 Hz, CH_2CH_3). **Figure S45**.

^{13}C{1H} NMR (101 MHz, $CDCl_3$) δ 190.5, 161.3, 149.3, 147.6, 138.0, 135.7, 132.9, 128.7, 128.6, 123.2, 110.3, 49.5, 31.7, 29.2, 29.1, 29.1, 26.4, 22.6, 14.1. **Figure S46**.

HRMS (ESI) m/z: [M + H]$^+$ Calcd for $C_{21}H_{27}N_2O_3$ 355.2022; Found 355.2013. [M + Na]$^+$ Calcd for $C_{21}H_{26}N_2O_3Na^+$ 377.1841; Found 377.1832.

3. NMR investigations

Unless otherwise stated, all the experiments were performed on a Bruker Avance 400 MHz spectrometer, and all the samples were prepared form the following stock solutions:

- **Dimer stock1**: 2.5 mM **1** in $CDCl_3$.
- **Monomer stock**: 2.5 mM **2** in $CDCl_3$.
- **A stock1**: 5 mM **9-Et-A** in $CDCl_3$.
- **A stock2**: 0.5 mM **9-Et-A** in $CDCl_3$. Prepared through 1/10 dilution of **A stock1** in $CDCl_3$.
- **A stock3:** 50 mM **9-Et-A** in $CDCl_3$.
- **A stock4:** 25 mM **9-Et-A** in $CDCl_3$. Prepared through 1/2 dilution of **A stock3** in $CDCl_3$.
- **U stock**: 50 mM **1-Et-U** in $CDCl_3$.
- **C stock**: 0.75 mM **1-Et-C** in $CDCl_3$. To avoid precipitation of **1-Et-C**, the solution should be sonicated for 1 hour at 50°C before use.

3.1 Dilution experiments of artificial receptor 1 and compound 2

The self-association of **1** and **2** was monitored through ^{1}H NMR dilution experiments ($CDCl_3$, 50°C) in the 2.5 – 0.04 mM concentration range (**Figure S2** and **S3,** respectively). The required 600 μL x 2.5 - 0.04 mM samples were prepared by diluting 600 – 10 μL aliquots of **Dimer stock1** or **Monomer stock** with 0 – 590 μL of $CDCl_3$. Then, each sample was submitted to ^{1}H NMR analysis at 50°C (**Figures S2** and **S3**), collecting 16, 64, or 128 scans for the solutions containing the compounds at 2.5 – 0.5 mM, 0.25 – 0.1 mM, or 0.04 concentration, respectively.

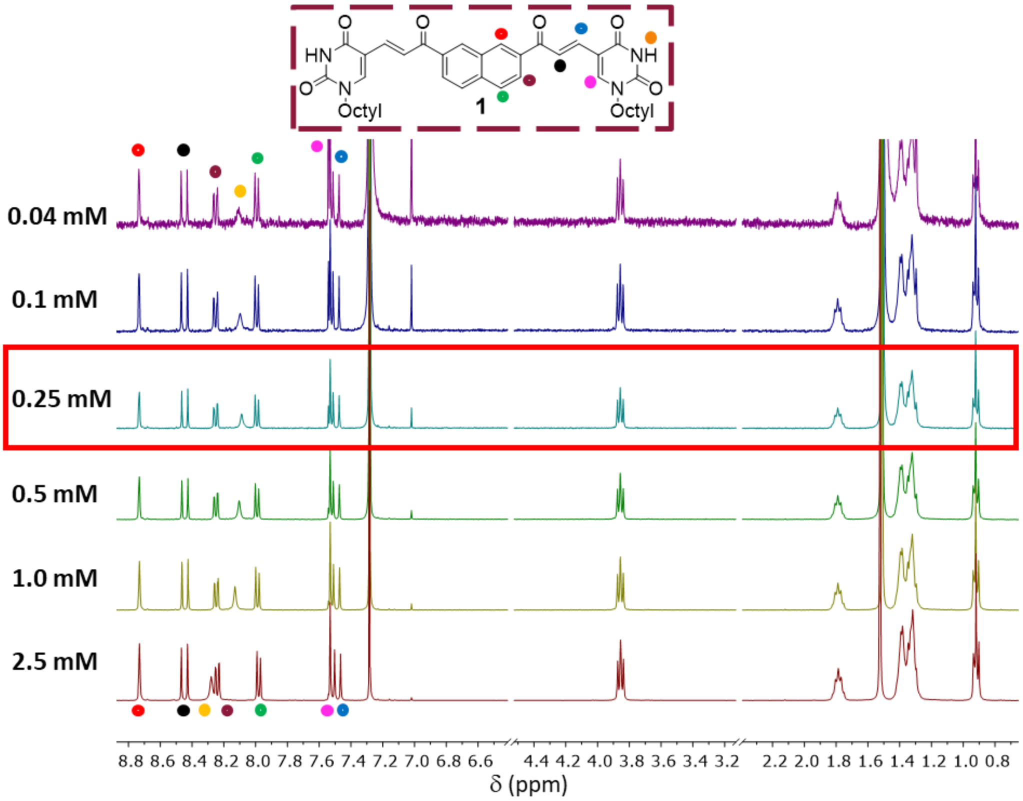

Figure S2. *Stacked ^{1}H NMR spectra obtained for the artificial receptor **1** at decreasing concentration levels (400 MHz, 50°C, $CDCl_3$).*

Dilution experiments of both compounds **1** and **2** revealed a progressive downfield shift for the imide resonances (H_{im}) at [**1**] and [**2**] ≥ 0.5 mM, suggesting the onset of intermolecular hydrogen bonds between the uracil units of the tested compounds (in the case of **1**, intramolecular interactions are prevented by the rigidity of the naphthalene scaffold). At contrary, for [**1**] and [**2**] ≤ 0.25 mM the chemical shift of the imide protons remained unaffected by dilution, indicating negligible self-association under these conditions and excluding that these processes could interfere with adenine recognition. Hence, during NMR titrations (**Main text,** and **Section S3.2**), the concentration of **1** and **2** was set at 0.25 mM to inhibit their self-association while providing the highest attainable signal intensity. For both compounds, the chemical shifts of the imidic protons (yellow circles) were not affected by dilution below 0.25mM, indicating negligible self-association.

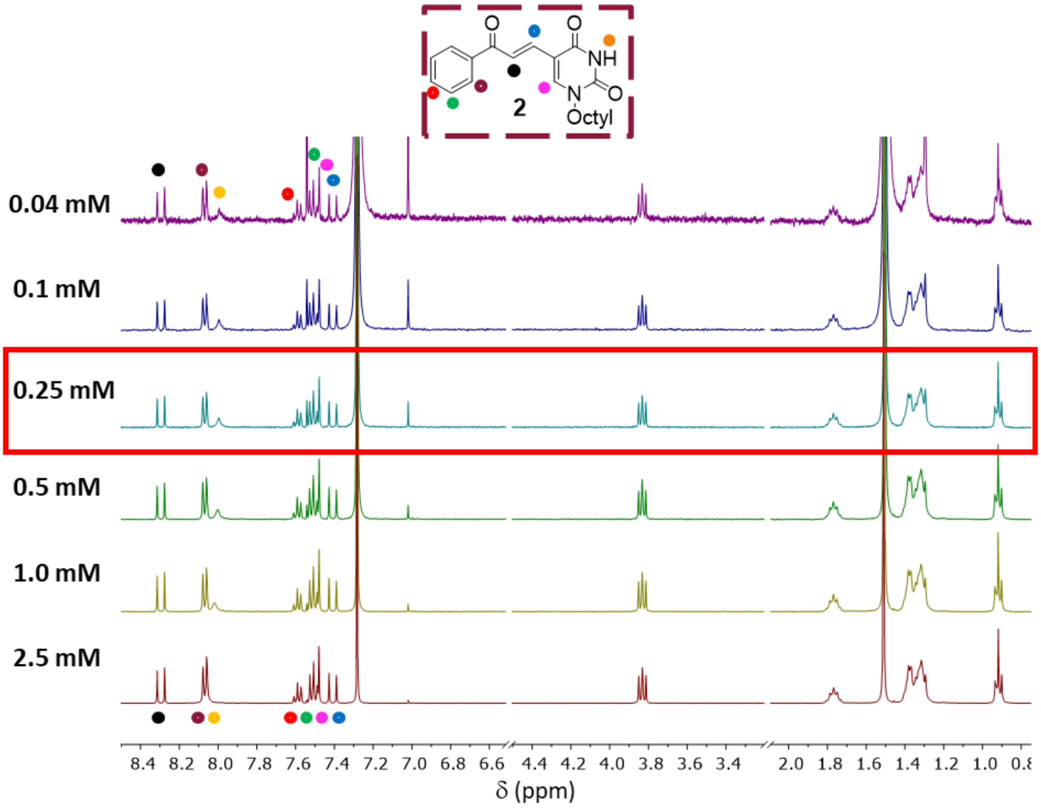

Figure S3: *Stacked ^{1}H NMR spectra obtained for compound **2** at decreasing concentration levels (400 MHz, 50°C, $CDCl_3$).*

3.2 NMR titrations

All the titrations were performed at 50°C on 600 μL solutions of $CDCl_3$ containing 0.25 mM **1** or **2** and increasing amounts of **9-Et-A**, **1-Et-U**, or **1-Et-C**. **1-Et-G** was not included in these experiments, as it was not soluble in $CDCl_3$. Poor solubility in $CDCl_3$ was also observed for **1-Et-C**, and, for this substrate, only partial titrations were performed. All the ^{1}H NMR spectra were acquired by collecting 64 scans. Each sample was prepared by mixing proper amounts of the corresponding stock solutions and made up to volume with $CDCl_3$ (vide infra). The binding isotherms were obtained by plotting the chemical shift perturbations (Δδ) experienced by the protons of compounds **1** and **2** as a function of **9-Et-A** concentration (**Figure 2a** and **S5**, respectively), and by plotting those experienced by the protons of artificial receptor **1** as a function of **1-Et-U** concentration (**Figure S8c**).

*3.2.1 Titrations of artificial receptor **1** with **9-Et-A***

0.25 mM solutions of **1** in the presence of 0 – 15.0 mol eq. of **9-Et-A** were prepared by mixing 60 μL of **Dimer stock** with 0 – 450 μL aliquots of **A stock2** (0 – 1.5 mol eq. range) or 60 – 450 μL aliquots of **A stock1** (2.0 – 15.0 mol eq. range) and made up to volume with 540 – 90 μL or 480 – 90 μL of $CDCl_3$, respectively. Then, each sample was submitted to ^{1}HNMR analysis (**Figures 2a** and **S1**) . A preliminary titration was performed at 25°C (**Figure S4a-c**), giving a $K_a > 10^6$ M^{-1}. Reliable estimation of the association constant was obtained from titration performed in triplicate at 50°C (**Figures S7a**), $K_a = (4.0 \pm 0.7) \cdot 10^4$ M^{-1}.

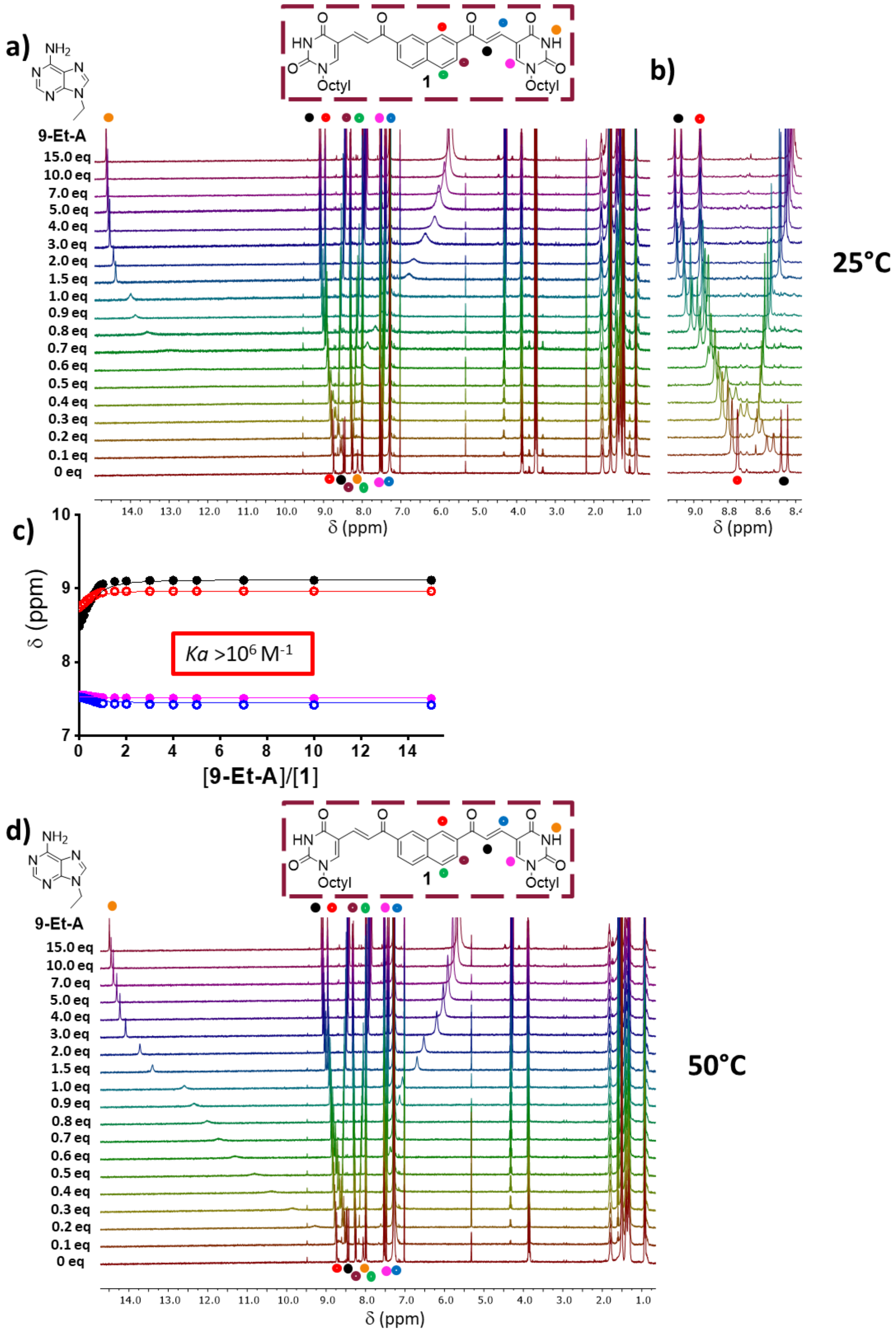

Figure S4: ***a)*** *Full-scale stacked ^{1}H NMR spectra obtained for the titrations of 0.25 mM artificial receptor* ***1*** *with 0 - 15 mol eq of* ***9-Et-A*** *at 25°C (400 MHz, $CDCl_3$). The disappearance of the imidic resonances of* ***1*** *(yellow circles) for 0.1 - 0.7 mol eq of* ***9-Et-A*** *might indicate intermediate exchange or multiple conformations adopted by* ***1***. ***b)*** *Region of the same spectra revealing the extension of spectral broadening to one signal of the α–β unsaturated systems of* ***1*** *(black circles).* ***c)*** *Fitting of the Δδs experienced by the resonances of* ***1*** *as a function of* ***9-Et-A*** *concentration. Imidic protons were excluded from the fitting due to their disappearance in the presence of 0.1-0.7 eq of* ***9-Et-A. d)*** *Full-scale stacked ^{1}H NMR spectra obtained for the titrations of 0.25 mM artificial receptor* ***1*** *with 0 - 15 mol eq of* ***9-Et-A*** *at 50°C(400 MHz, $CDCl_3$).*

*3.2.2 Titrations of control compound **2** with **9-Et-A***

0.25 mM solutions of **2** in the presence of 0 – 150 mol eq. of **9-Et-A** were prepared by mixing 60 μL of **Dimer stock** with 0 – 300 μL aliquots of **A stock4** (0 – 50 mol eq. range) or 180 – 450 μL aliquots of **A stock3** (60 – 150 mol eq. range) and made up to volume with 540 – 240 μL or 360 – 90 μL of $CDCl_3$, respectively. Then, each sample was submitted to ^{1}H NMR analysis. The titration was performed in triplicate (**Figure S5** and **S6**). K_a = 58 ± 2 M^{-1} (**Figure S7b**).

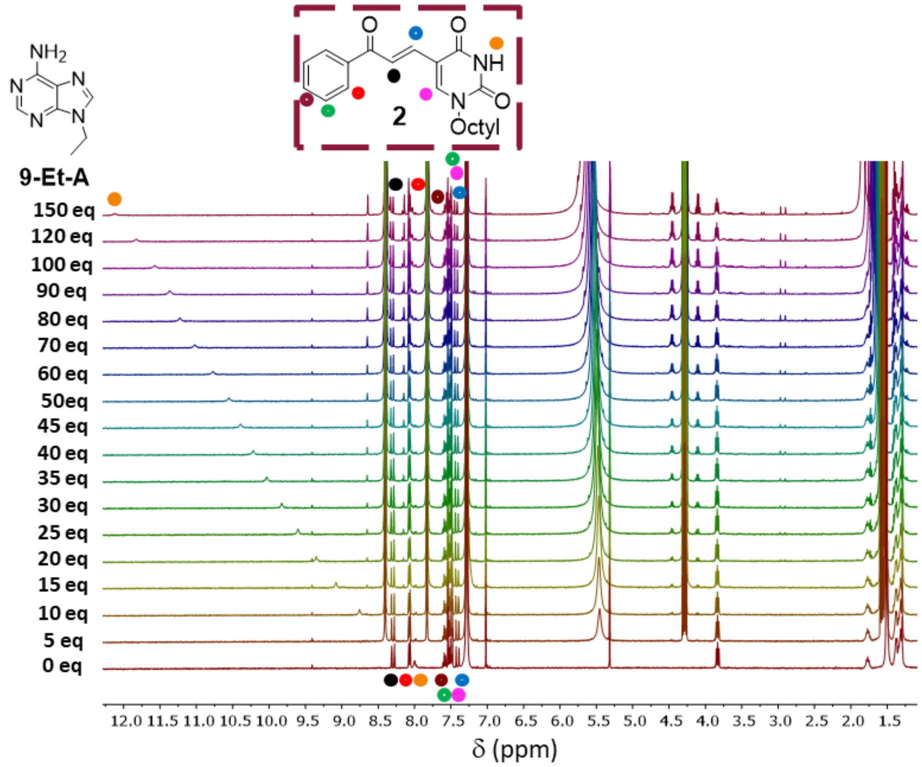

Figure S5: *Full-scale stacked ^{1}H NMR spectra obtained for the titrations of 0.25 mM control compound **2** with 0 - 150 mol eq of **9-Et-A** (400 MHz, 50° C, $CDCl_3$).*

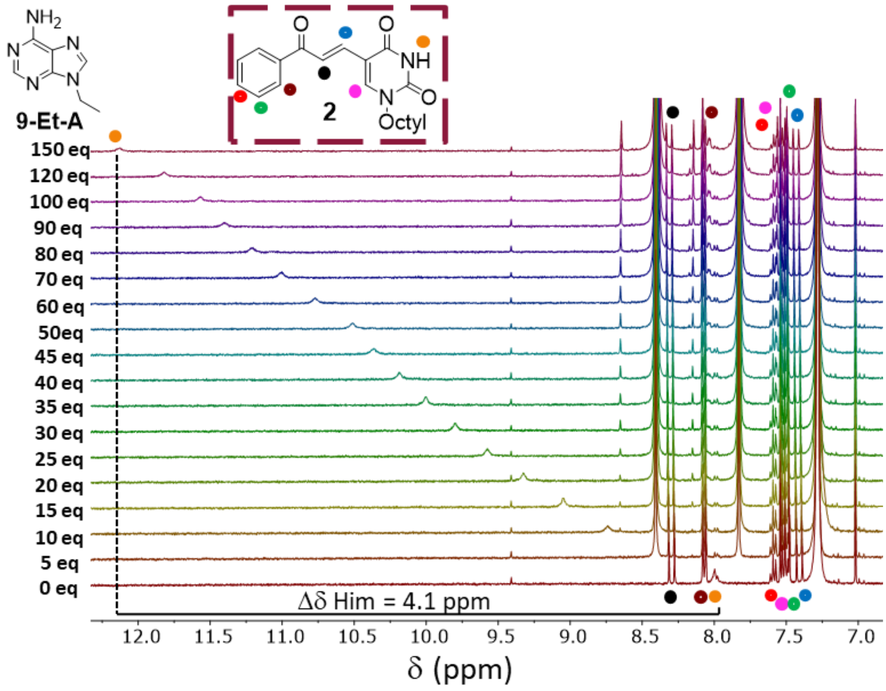

Figure S6: *Region from stacked ^{1}H NMR spectra obtained for the titrations of 0.25 mM solution of control compound* ***2*** *with 0 - 150 of* ***9-Et-A,*** *($CDCl_3$, 50° C). Conditions are the same as for titration of artificial receptor* ***1*** *reported in* ***Figure 2a*** *in the main text.*

3.2.3 Fitting of NMR data

The experimental data were fit to a 1:1 binding model using the “bindfit” tool of the www.supramolecular.org online facility.[20,21] Non-linear regression was performed according to the following equations:

$$\Delta\delta = \delta_{max}\left(\frac{[HG]}{[H]_0}\right),\ [HG] = \frac{1}{2}\left([G]_0 + [H]_0 + \frac{1}{K_a}\right) - \sqrt{\left([G]_0 + [H]_0 + \frac{1}{K_a}\right)^2 + 4[H]_0[G]_0}$$

where $[H]_0$ and $[G]_0$ are the total concentrations of the tested receptors and substrates, respectively, [HG] is the concentration of the forming complexes, and the endpoint chemical shifts δ_{max} and the association constant K_a are the fitting parameters (**Figures S7** and **S8c**)

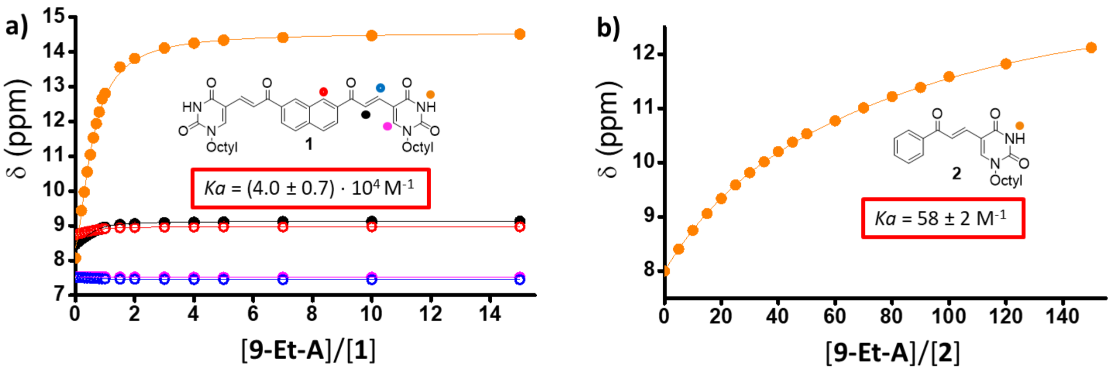

Figure S7*. Fitting of the dDs experienced by the resonances of* ***a)*** *artificial receptor* ***1*** *and* ***b)*** *control compound* ***2*** *against increasing* ***9-Et-A*** *concentration (dots: experimental points, curves: calculated values)****.***

*3.2.3 Titrations of artificial receptor **1** with **1-Et-U***

0.25 mM solutions of **1** in the presence of 0 – 150 mol eq. of **1-Et-U** were prepared by mixing 60 μL of **Dimer stock** with 0 – 450 μL aliquots of **U stock** and made up to volume with 540 –90 μL of $CDCl_3$. Then, each sample was submitted to ^1H NMR analysis. The titration was performed in duplicate (**Figure S8a,b**). K_a = 12 ± 1 M^{-1} (**Figure S8c**).

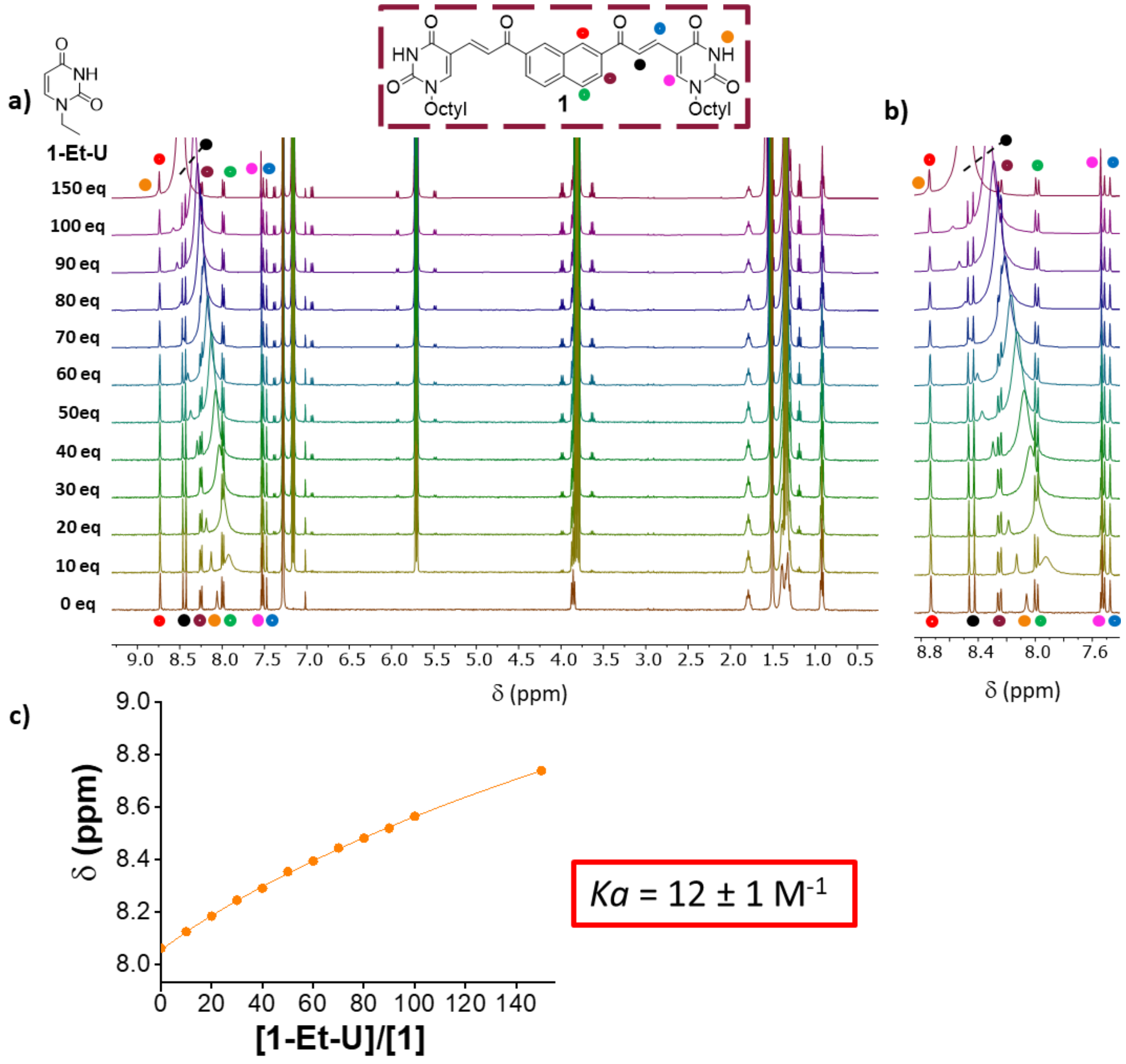

Figure S8. ***a)*** *Full-scale stacked ^1H NMR spectra obtained for the titrations of 0.25 mM artificial receptor **1** with 0 - 150 mol eq of **1-Et-U** (400 MHz, 50° C, $CDCl_3$).* ***b)*** *Region from the same spectra revealing a gradual downfield shift of the imidic resonances of **1** (yellow circles) over the course of the titrations.* ***c)*** *Fitting of the Δδs experienced by the resonances of **1** as a function of **1-Et-U** concentration.*

*3.2.4 Titrations of artificial receptor **1** with **1-Et-C***

0.25 mM solutions of **1** in the presence of 0 – 2.0 mol eq. of **1-Et-C** were prepared by mixing 60 μL of **Dimer stock** with 0 – 400 μL aliquots of **C stock** and made up to volume with 540 –140 μL of $CDCl_3$. Then, each sample was submitted to ^1H NMR analysis. The experiments were performed in duplicate (**Figure S9a,b**). Full titrations were not performed due to the poor solubility of **1-Et-C** in $CDCl_3$, impairing K_a calculation.

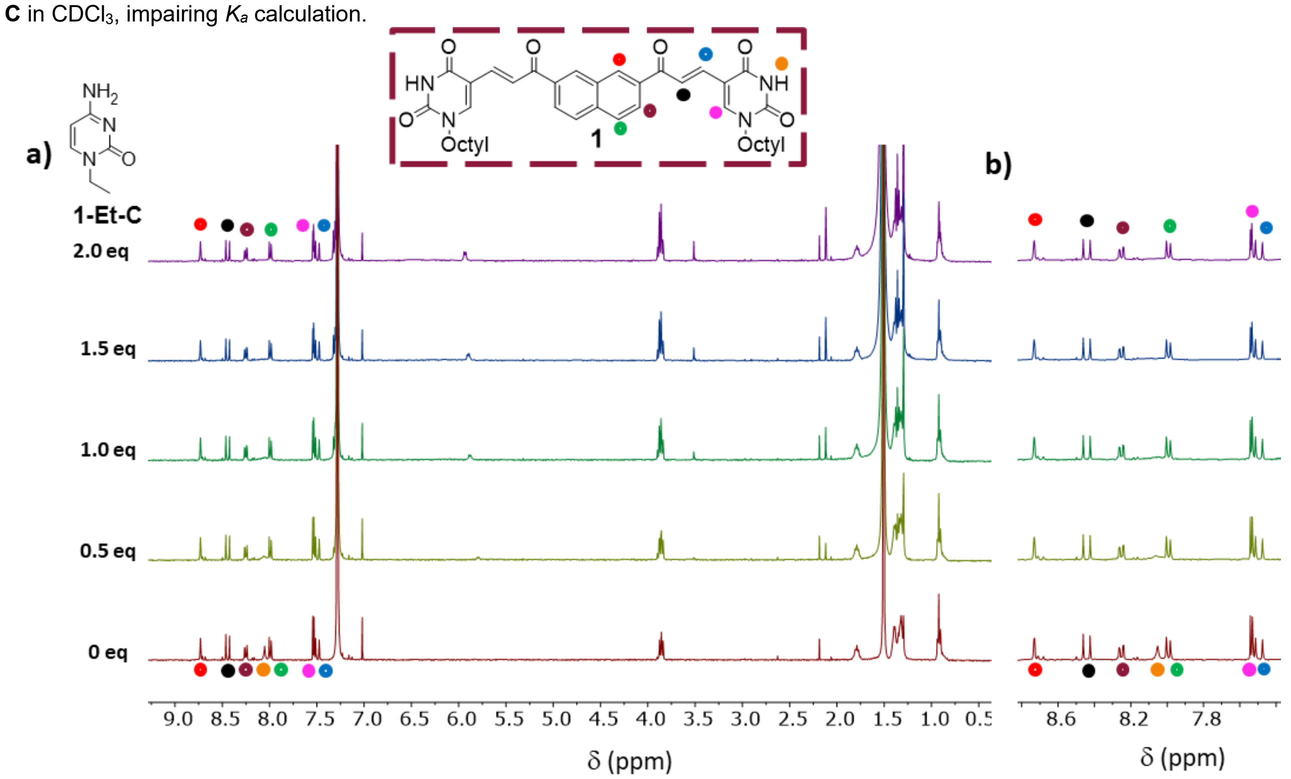

Figure S9. ***a)*** *Full-scale stacked ^1H NMR spectra obtained for the titration of 0.25 mM artificial receptor **1** with 0 – 2.0 mol eq of **1-Et-C** (400 MHz, 50° C, $CDCl_3$). Higher molar excesses of **1-Et-C** were not explored due to its poor solubility in $CDCl_3$.* ***b)*** *Region from the same spectra revealing broadening of the imidic resonances (yellow circles) of **1** in the investigate concentration range.*

3.3 NOESY experiments

1.9 mM solutions of **1** or **2** in the presence of 1 mol eq. of **9-Et-A** were prepared by mixing 450 μL of **Dimer stock** or **Monomer stock** with a 23 μL aliquot of **A stock 3**, respectively, and made up to volume with 127 μL of $CDCl_3$. Then, the samples were submitted to 2D-NOESY (**Figure 2b** and **S10a** for artificial receptor **1**, and **Fig. S11a,b** for compound **2**) and 1D-selective NOESY (**Figure S10b)** experiments at 25°C, using a mixing time of 500 ms. Fore selective NOE/ROE experiments the ^{1}H SELNOGR and ^{1}H SELROGR NMR programs were used, with mixing time of 500 ms.

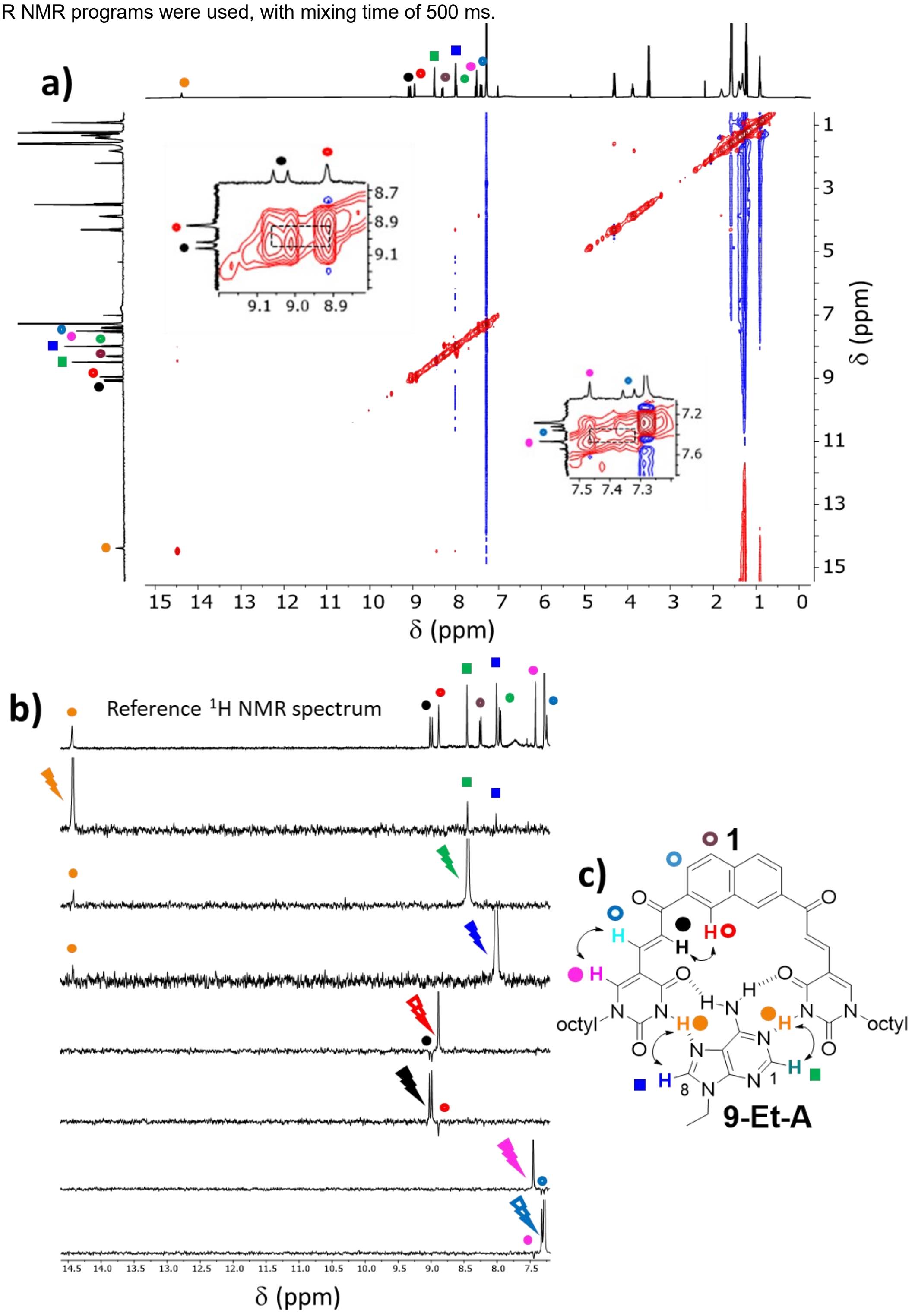

Figure S10. ***a)*** *Full-scale 2D NOESY spectrum of an equimolar mixture of artificial receptor* ***1*** *and* ***9-Et-A*** *([****1****] = [****9-Et-A****] = 1.9 mM, 400 MHz, 25°C,* $CDCl_3$*).* ***b)*** *Region from stacked 1D NOESY spectra obtained upon selective irradiation of diagnostic protons ([****1****] = [****9-Et-A****] = 1.9 mM, 400 MHz, 25°C,* $CDCl_3$*). Colored bolts and circles indicate the irradiated signals and those correlating with them, respectively.* ***c)*** *Proposed structure of the* ***1/9-Et-A*** *complex based on these experiments.*

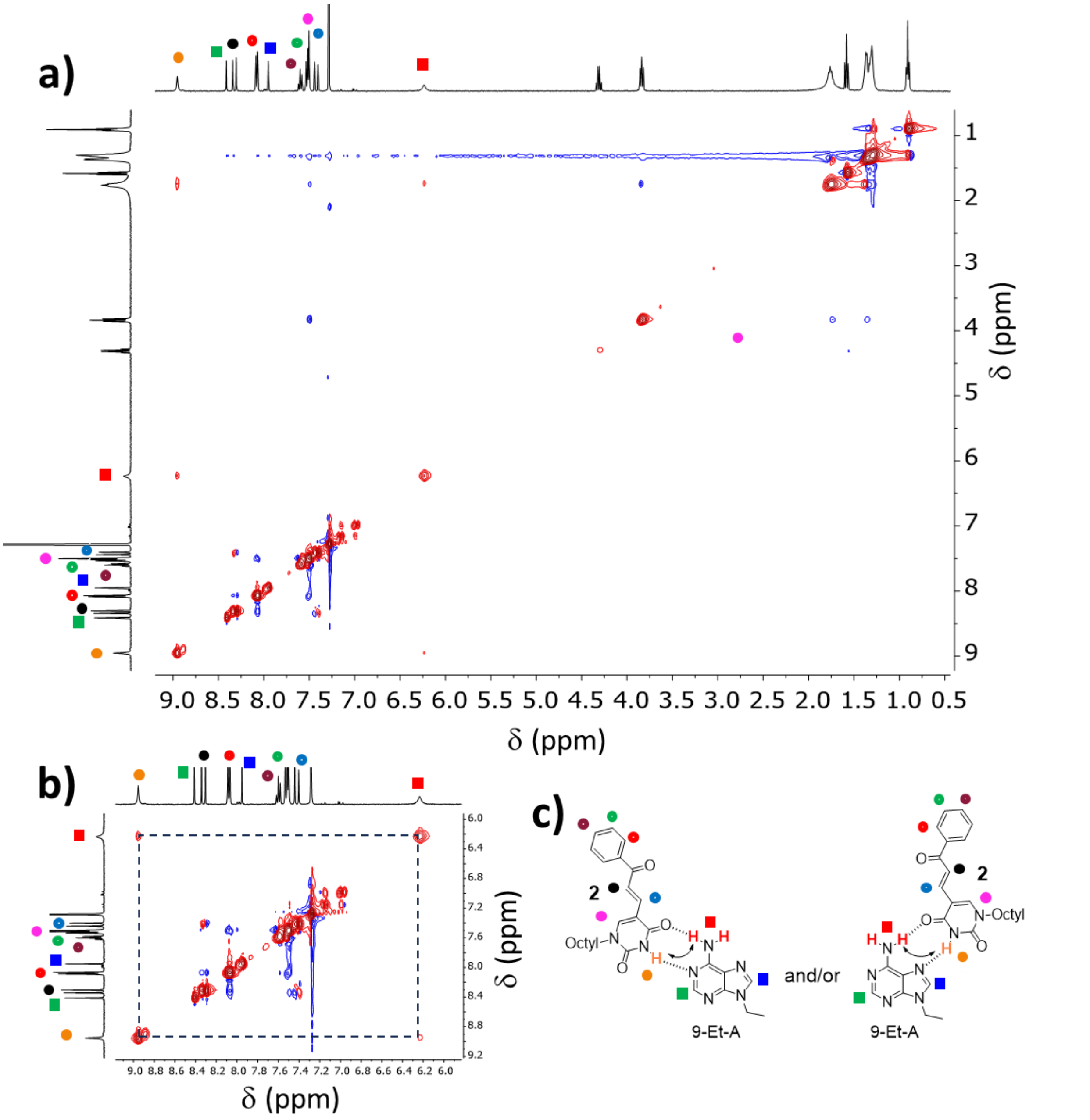

Figure S11. ***a)*** *Full-scale 2D NOESY spectrum of an equimolar mixture of control compound* ***2*** *and* ***9-Et-A*** *([****2****] = [****9-Et-A****] = 1.9 mM, 400 MHz, 25°C, $CDCl_3$).* ***b)*** *Region from the same spectrum revealing a cross-peak between the imidic proton of* ***2*** *and the NH_2 ones of* ***9-Et-A*** *(yellow circles and red squares, respectively).* ***c)*** *Possible structures of the* ***2/9-Et-A*** *complex based on these experiments.*

4. Nucleoside Selective Phase-Transfer experiments

All the samples were prepared in the form of the following stock solutions:

- **Dimer stock**: 1.0 mM **1** in $CDCl_3$.
- **Monomer stock**: 1.0 mM **2** in $CDCl_3$.
- **Ade stock**: 5 mM adenosine (**Ade**) and ammonium formate (internal standard) in D_2O.
- **C/U/G stock**: 5 mM cytidine, uridine, and guanosine in D_2O. To avoid precipitation of guanosine, the solution should be sonicated for 30 minutes at 50°C before use.

4.1 Precipitation of adenosine

The precipitation experiments described in the main text were first set up using plain adenosine (**Ade**). Precisely, D_2O solutions of **Ade** were stirred against $CDCl_3$ solutions of **1** (500 rpm, 2 hours, r. t., [**1**] = [**Ade**] **=** 1mM) to assess whether this artificial receptor could extract the target ribonucleoside in the organic layer. Although nucleoside extraction was not observed, the formation of a precipitate at the interphase suggested the formation of an insoluble complex including **1** and **Ade**. In fact, ^1H NMR analysis in DMSO-*d6* of the isolated solid revealed that it was composed of a 1/1 mixture of the tested receptor and substrate, as inferred from peak integration and comparison with the reference spectra of the two components in the same medium (**Figure S12b**). This solid was not soluble in less competitive solvents (i. e. CD_3OD, acetonitrile-*d3*, Acetone-*d6*, $CDCl_3$), avoiding further investigations on the mechanisms underlying **Ade** precipitation. Nevertheless, control experiments performed by stirring the same D_2O solution against blank $CDCl_3$ or a $CDCl_3$ solution of **2** did not lead to precipitate formation.

Procedure:

In a glass screw cap tube, a 1.4 mL of 1.0 mM solution of **Ade** and ammonium formate was prepared by diluting 280 μL of **Ade stock** with 1.12 mL of D_2O, and a 600 μL aliquot was submitted to ^1H NMR analysis to obtain a control spectrum of the mother adenosine solution (**Figure S12a**, top panel). Then, 800 μL of $CDCl_3$ (blank experiments), **Dimer stock** (experiments involving artificial receptor **1**), or **Monomer Stock** (experiments involving control compound **2**) were added to the remaining 800 μL of adenosine solution, and the resulting biphasic mixtures were stirred at 500 rpm, 25°C for 2 hours. Subsequently, the stirring was stopped, and the mixtures were transferred to an Eppendorf microtube. The experiments involving receptor **1** produced a white precipitate, which was collected at the interphase via centrifugation at 5000 rpm for 5 minutes. Then, 600 μL of the D_2O layer were withdrawn and submitted to ^1H NMR analysis (**Figure S12a**, bottom panel). The precipitate was isolated by removing, in the order, the $CDCl_3$ and the remaining D_2O solutions, dried under vacuum, and taken in DMSO-*d6* to record an ^1H NMR spectrum (**Figure S12b**, central panel). The precipitate was identified as a 1:1 mixture of **1** and adenosine by comparing the reference spectra of these two components in DMSO-*d6* (**Figure S12b**, top and bottom panels, respectively). The blank experiments and those involving the use of compound **2**, instead, produced clear mixtures. In these cases, the $CDCl_3$ and D_2O layers were decanted, and 600 μL of the latter were withdrawn and submitted to ^1H NMR analysis (**Figure S12a**, second and third panels, respectively). All the experiments were performed in triplicate.

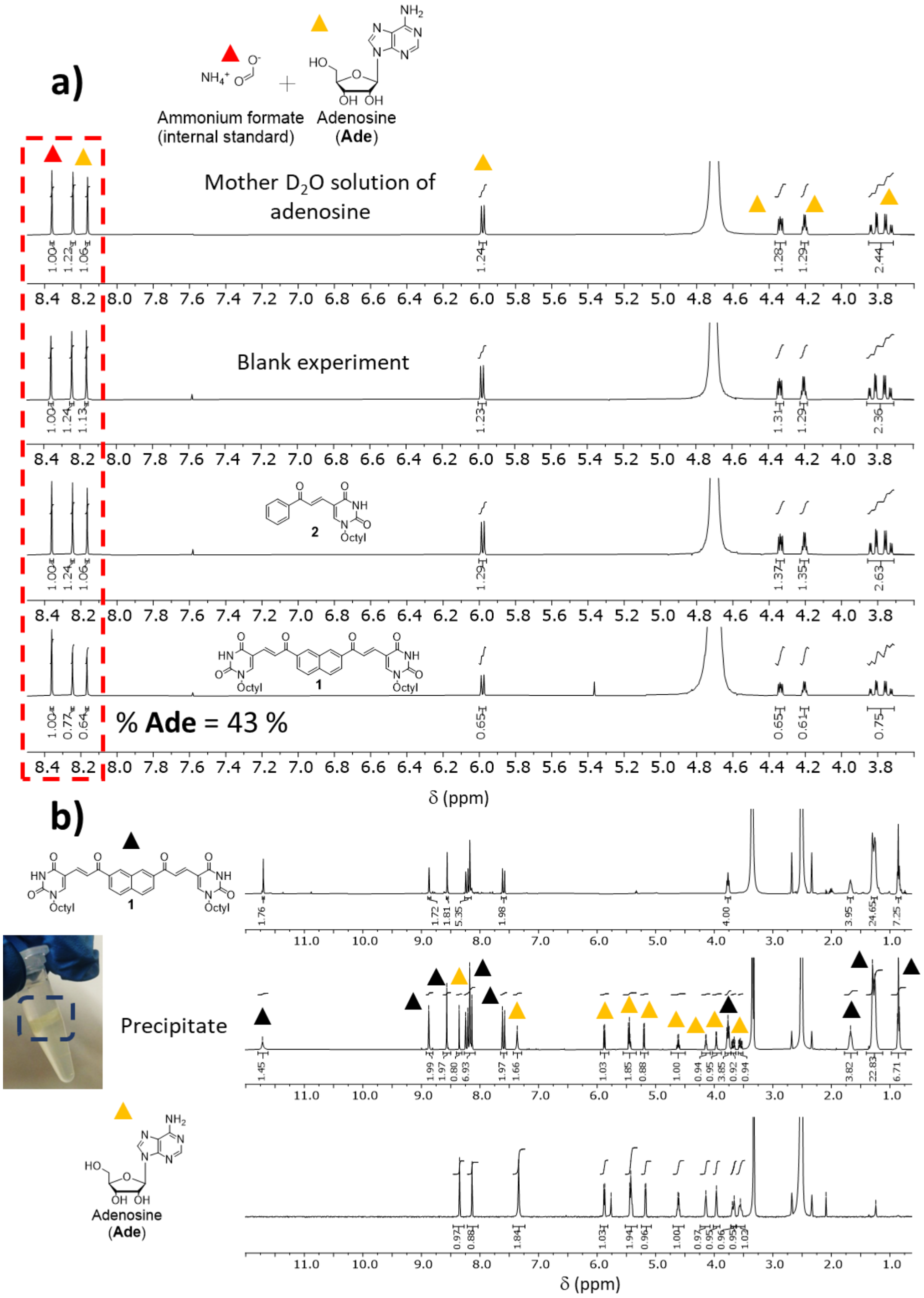

Figure S12. ***a)*** *Stacked ^{1}H NMR spectra (400 MHz, 25°C) of a mother equimolar mixture of* ***Ade*** *(yellow triangles) and ammonium formate (red triangles) in D_2O (1 mM each, top panel) and aliquots of the same mixture which were stirred against blank $CDCl_3$ (second panel) and $CDCl_3$ solutions of compounds* ***2*** *and* ***1*** *(1 mM each, third and bottom panel, respectively). Ammonium formate was used as internal standard for peak integration. Receptor-mediated precipitation of adenosine (****%Ade****) was monitored by comparing the averaged integrals of the* ***Ade*** *aromatic protons measured in D_2O solutions stirred against blank $CDCl_3$ and $CDCl_3$ solutions of* ***1. b)*** *Stacked ^{1}H NMR spectra of, from top to bottom, artificial receptor* ***1*** *(black triangles), the solid isolated from precipitation experiments, and* ***Ade*** *(yellow triangles), (400 MHz, 25°C, DMSO-d^6). Comparison between these spectra allows to identify the isolated precipitate as an equimolar mixture of* ***1*** *and* ***Ade****.*

4.2 Precipitation of adenosine from a mixture of four nucleosides

In a glass screw cap tube, an equimolar solution of ammonium formate, **Ade**, cytidine, uridine, and guanosine (1.4 mL x 1.0 mM each) was prepared by mixing 280 μL of **Ade stock**, 280 μL of **CUG stock**, and 840 μL of D_2O, and a 600 μL aliquot was submitted to ^{1}H NMR analysis to obtain a control spectrum of the mother nucleoside mixture (**Figure S13b**). Then, precipitation experiments in the presence of blank $CDCl_3$, artificial receptor **1**, or control compound **2** were performed with the remaining 800 μL of nucleoside mixture, as reported in **section S4.1**. Also in this case, only the use of receptor **1** led to the formation of a white precipitate (**Fig. 3**, **S14b**), which was isolated as reported in the **section S4.1** and identified as a 1:1 mixture of **1** and adenosine (**Figure 3,** bottom, and **S14b**). The experiments were performed in triplicate.

4.3 Quantification of precipitated adenosine

For the spectra generated by the equimolar mixtures of four ribonucleosides, peak assignment was performed through comparison with the separated spectra of 1 mM adenosine, cytidine, uridine, and guanosine in D_2O (**Fig S13a**). For each precipitation experiment (**sections S4.1** and **S4.2**), the ^{1}H NMR spectra of the withdrawn D_2O layers were normalized to the formyl signal of ammonium formate, which served as an internal standard for peak integration (**Figures 3**, top, **S14a**, and **S12a**)
The extent of adenosine precipitation caused by artificial receptor **1** was estimated according to the following equation:

$$\%\boldsymbol{Ade} = \left[1 - \left(\frac{I_{prec}}{I_{blank}}\right)\right] \cdot 100$$

where ***%Ade*** is the % of precipitated adenosine, while I_{blank} and I_{prec} are the averaged integral values of the adenosine aromatic protons in D_2O aliquots stirred against blank $CDCl_3$ and a $CDCl_3$ solution of **1**, respectively.
%Ade = 43% and 44% for precipitation experiments performed on plain adenosine solutions (**Fig. S12a**) and equimolar mixtures of four ribonucleosides (**Figure S14a**), respectively.
Receptor-induced precipitation was monitored by comparing the concentration of **Ade** in D_2O aliquots taken after experiments using **1** or **2** with that in control aliquots stirred against blank $CDCl_3$. In each case, ammonium formate was chosen as internal standard for peak integration, and the area of its formyl peak (red triangles, **Figure S14a**) was used to normalize the signals of **Ade** (yellow triangles, **Figure S14a**). The integrals measured in the mother mixture of **Ade** and ammonium formate (1mM each) remained unaffected upon performing blank experiments (**Figure S14a**, top and second panels), excluding undesired extraction of the internal standard to the organic layer. Instead, the averaged integral of the **Ade** aromatic protons was 1.29 and 0.74 in D_2O aliquots treated with blank $CDCl_3$ and a $CDCl_3$ solution of **1**, respectively (Fig. **S14a**, second and bottom panel). This indicated that the dimeric receptor precipitated circa 44% of adenosine, as estimated through division of the latter value by the former (**%Ade**, **section S4.1**). Conversely, control investigations involving $CDCl_3$ solutions of **2** did not evidence significant variations of these integrals compared to blank experiments (**Figure S14a**, second and third panel, respectively), confirming that the monofunctional compound **2** was not capable of causing **Ade** precipitation. The signals belonging to the ribose scaffold of **Ade** were not included in these calculations to facilitate the translation of the quantification method to complex mixtures of ribonucleosides. In fact, preliminary ^{1}H NMR analyses of an equimolar mixture of the four possible ribonucleosides evidenced signal overlapping in the spectral region including their sugar units, while the aromatic signals of these substrates remained relatively isolated (**Figure S14a,b**). Accordingly, the aromatic protons of **Ade**, cytidine, uridine and guanosine were used to monitor their concentration in the following precipitation experiments (**Figures 3**, top and **S14a**).

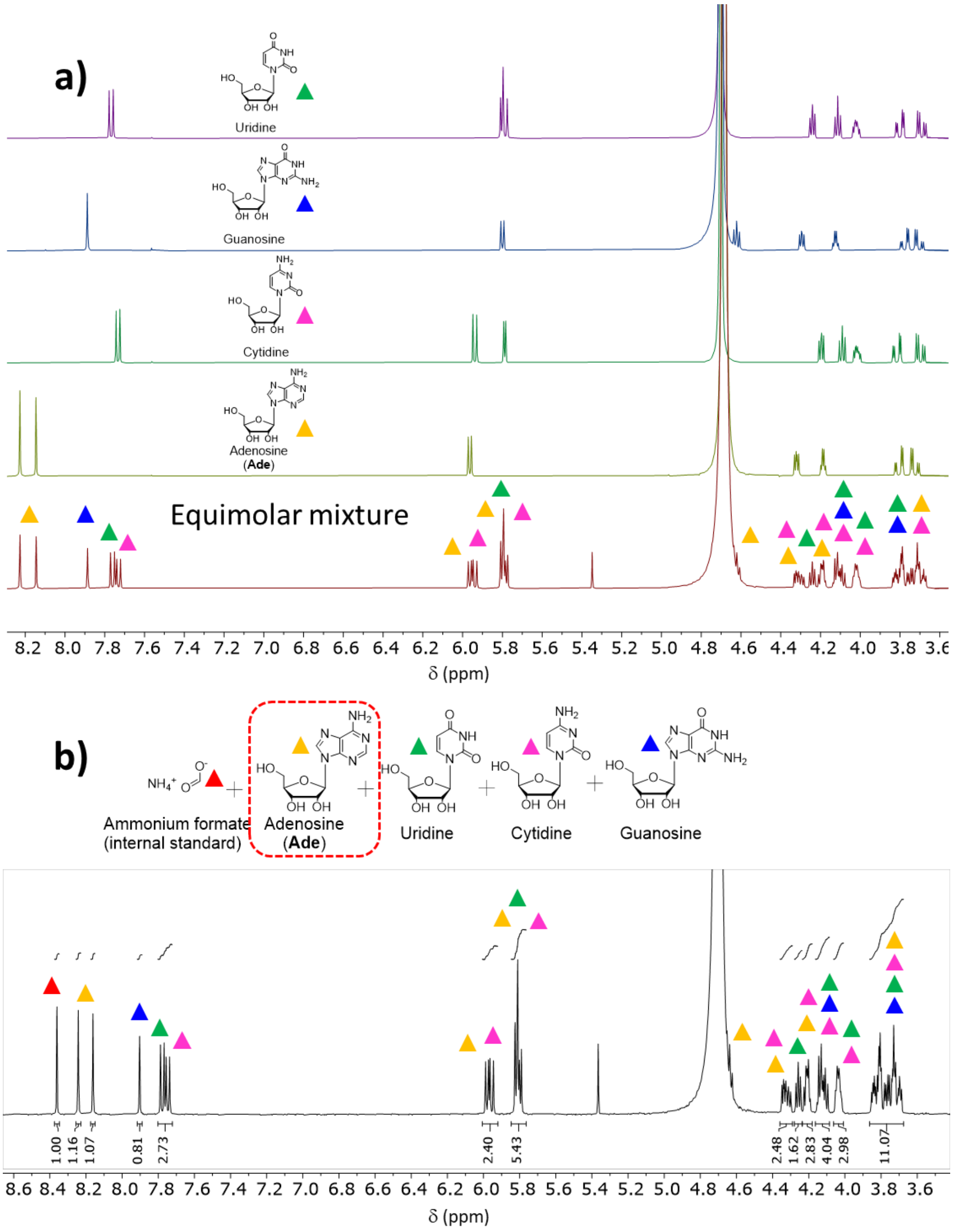

Figure S3. ***a)*** *Stacked* ^{1}H *NMR spectra of, from top to bottom, uridine (green triangles), guanosine (blue triangles), cytidine (magenta triangles), adenosine (**Ade**, yellow triangles), and an equimolar mixture of all the four ribonucleosides (1 mM each, 400 MHz, 25°C,* D_2O*).* ***b)*** ^{1}H *NMR spectrum of an equimolar mixture of ammonium formate (red triangles) and all the four ribonucleosides (1 mM each, 400 MHz, 25°C,* D_2O*).*

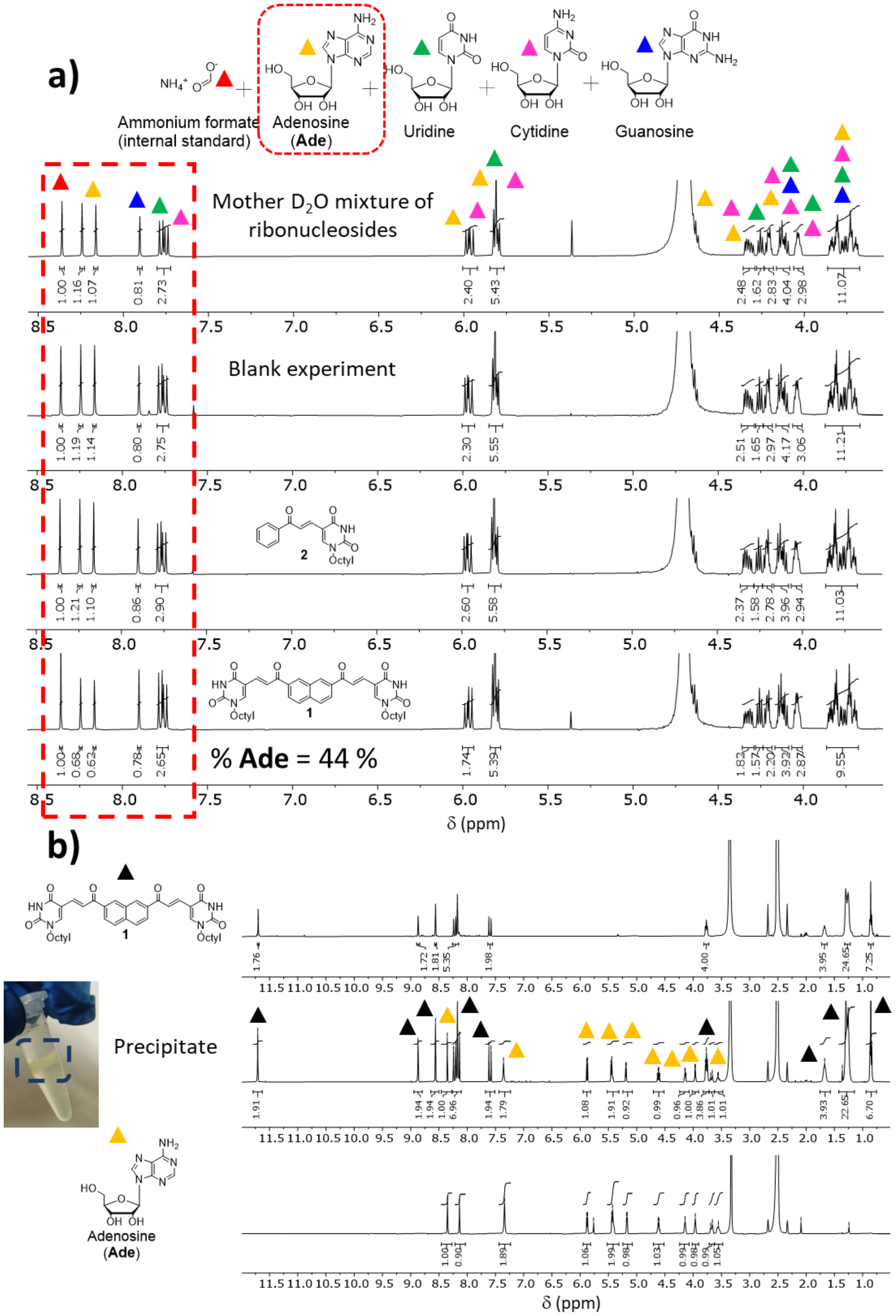

Figure S14: ***a)*** *Stacked* [1]*H NMR spectra (400 MHz, 25°C) of a mother equimolar mixture of* ***Ade*** *(yellow triangles), uridine (green triangles), cytidine (magenta triangles), guanosine (blue triangles), and ammonium formate (red triangles) in D_2O (1 mM each, top panel) and aliquots of the same mixture which were stirred against blank $CDCl_3$ (second panel) and $CDCl_3$ solutions of compounds* ***2*** *and* ***1*** *(1 mM each, third and bottom panel, respectively). Ammonium formate was used as internal standard for peak integration. Receptor-mediated precipitation of adenosine (****%Ade****) was monitored by comparing the averaged integrals of the* ***Ade*** *aromatic protons measured in D_2O solutions stirred against blank $CDCl_3$ and $CDCl_3$ solutions of* ***1****.* ***b)*** *Stacked* [1]*H NMR spectra of, from top to bottom, artificial receptor* ***1*** *(black triangles), the solid isolated from the precipitation experiments, and* ***Ade*** *(yellow triangles), (400 MHz, 25°C, DMSO-d_6). Comparison between these spectra allows to identify the isolated precipitate as an equimolar mixture of* ***1*** *and* ***Ade****.*

5. Additional Figures- NMR characterizations

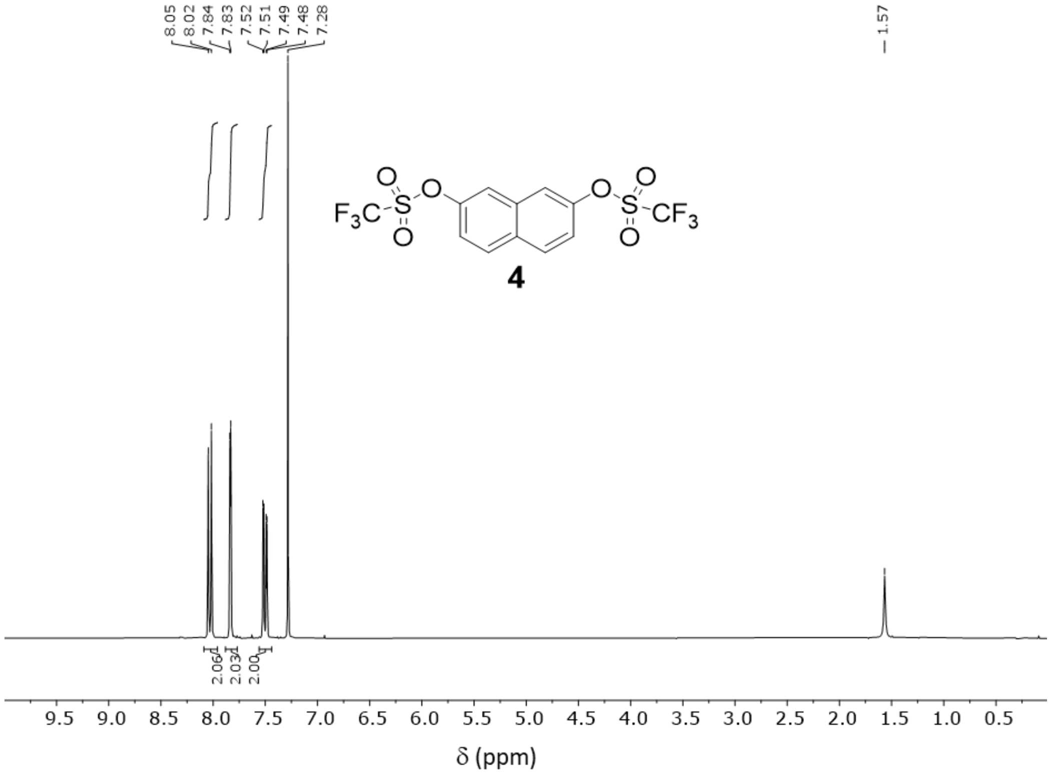

Figure S15. *^{1}H NMR spectrum of compound **4** (400 MHz, 25°C, $CDCl_3$).*

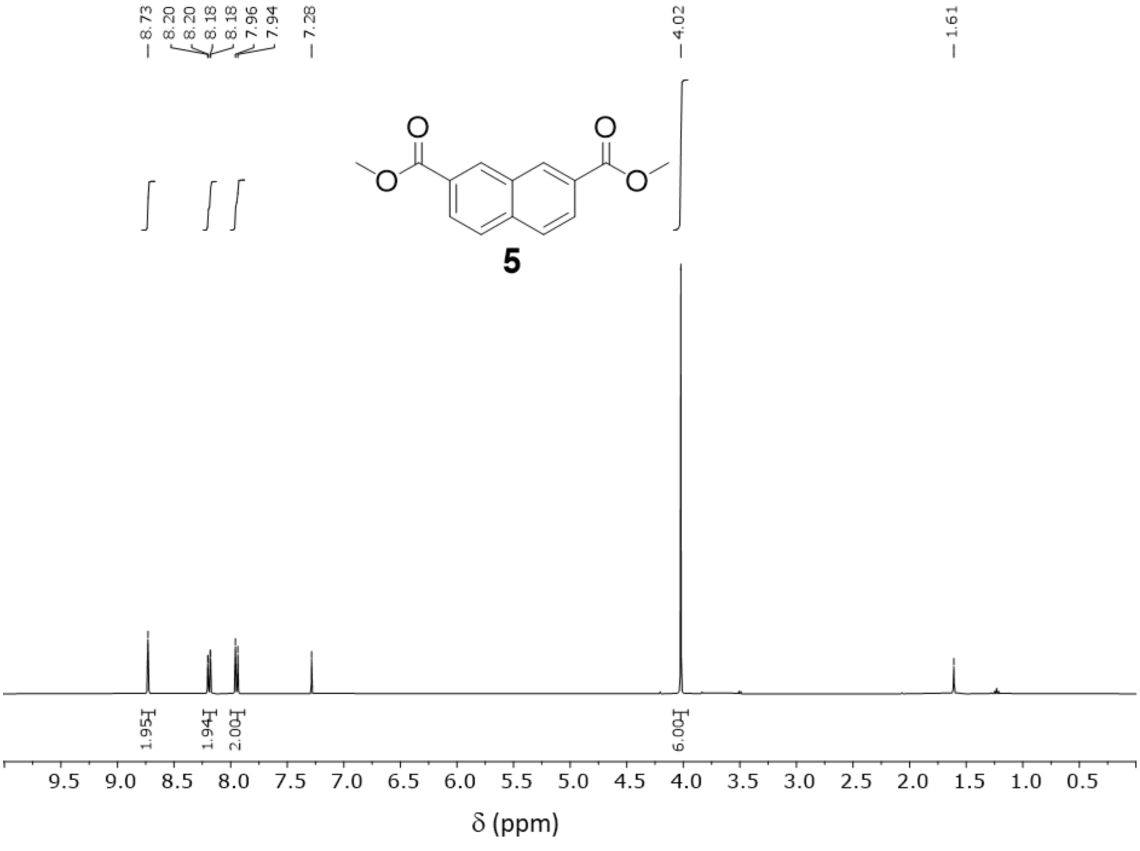

Figure S16. *^{1}H NMR spectrum of compound **5** (400 MHz, 25°C, $CDCl_3$).*

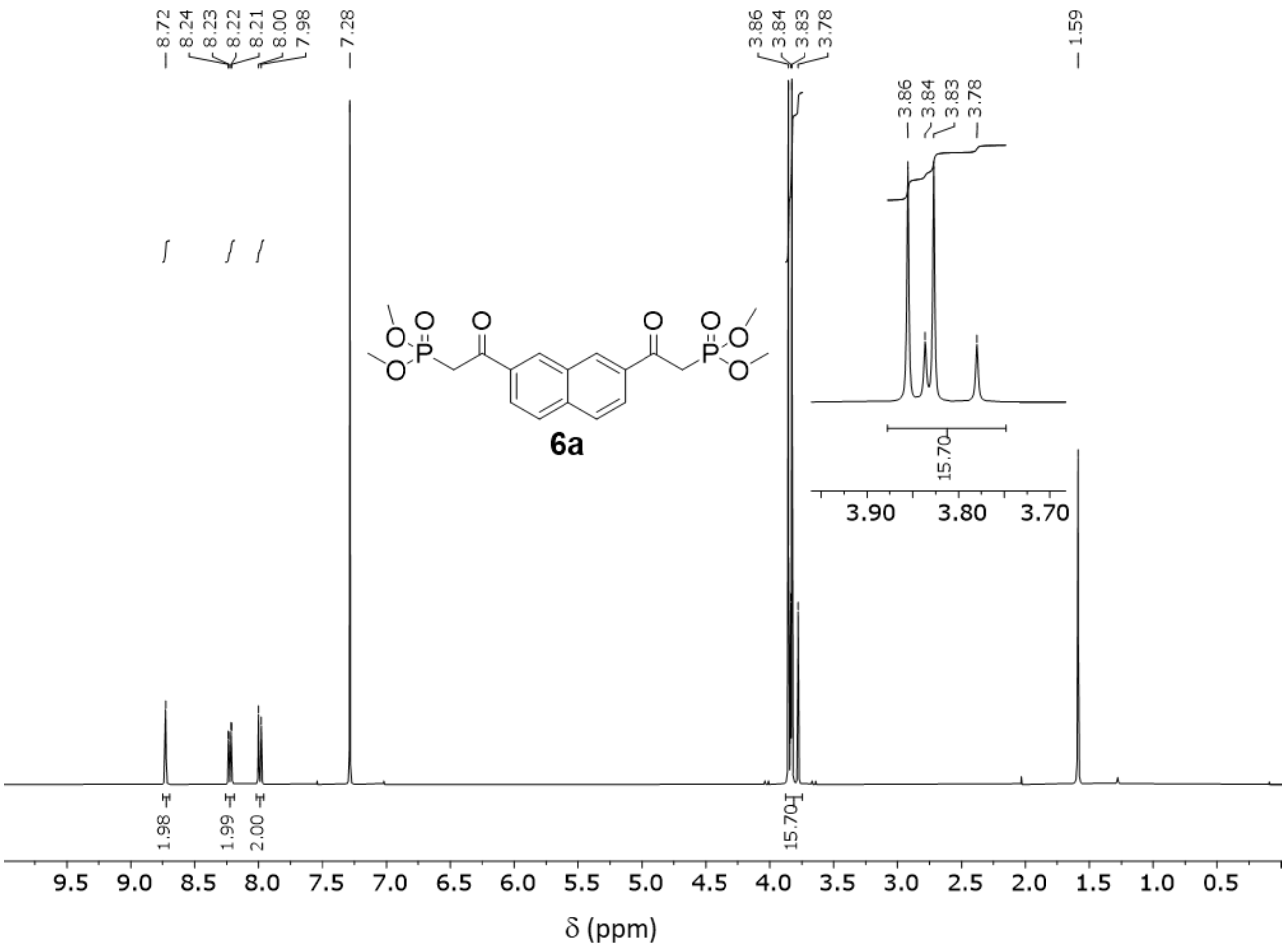

Figure S17. *^{1}H NMR spectrum of compound* ***6a*** *(400 MHz, 25°C, $CDCl_3$).*

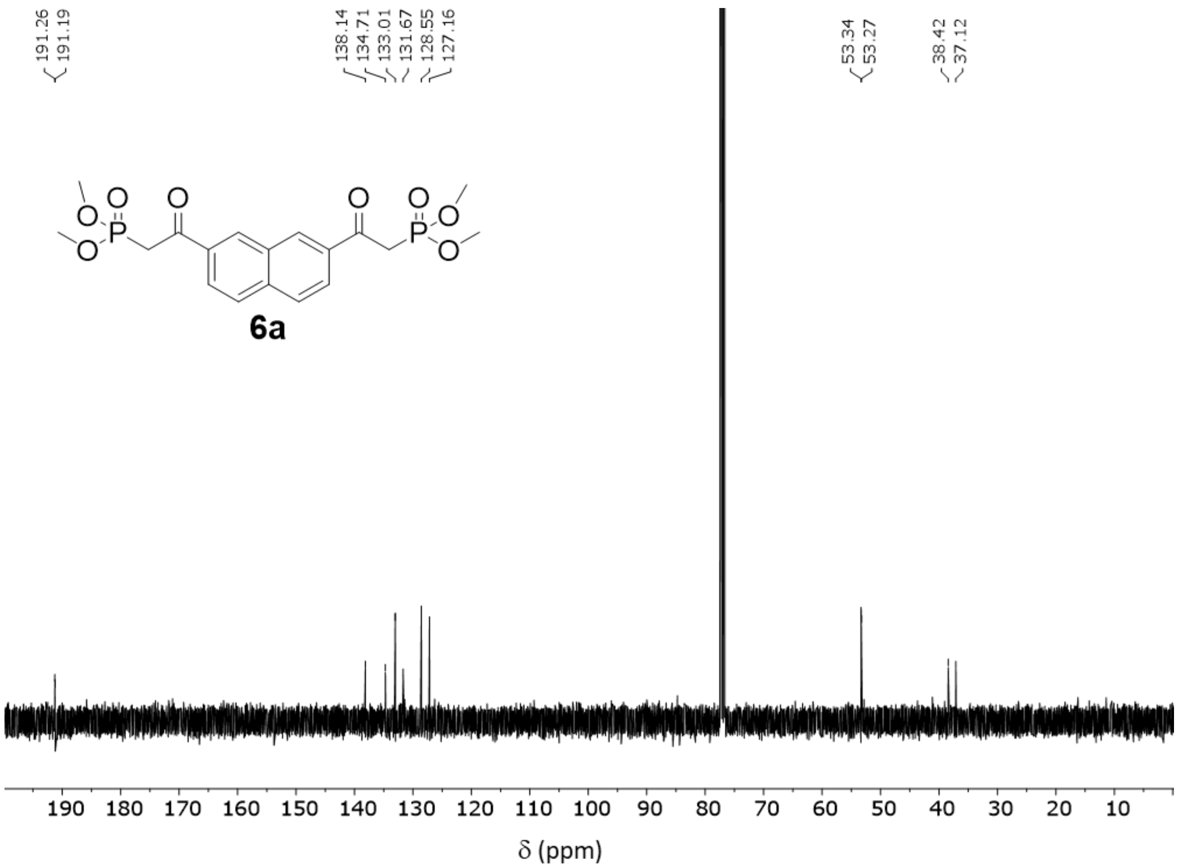

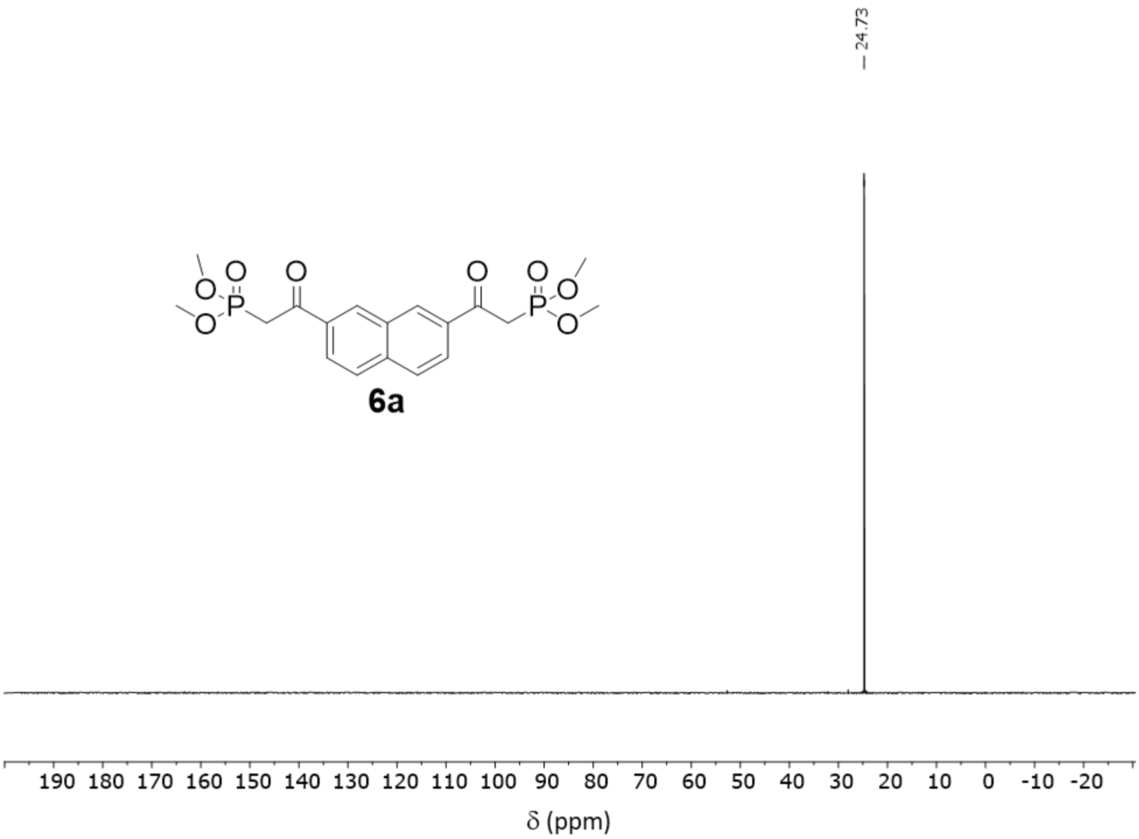

Figure S18. *^{13}C (top) and ^{31}P NMR (bottom) of compound **6a** (101 and 162 MHz, respectively, 25°C, $CDCl_3$).*

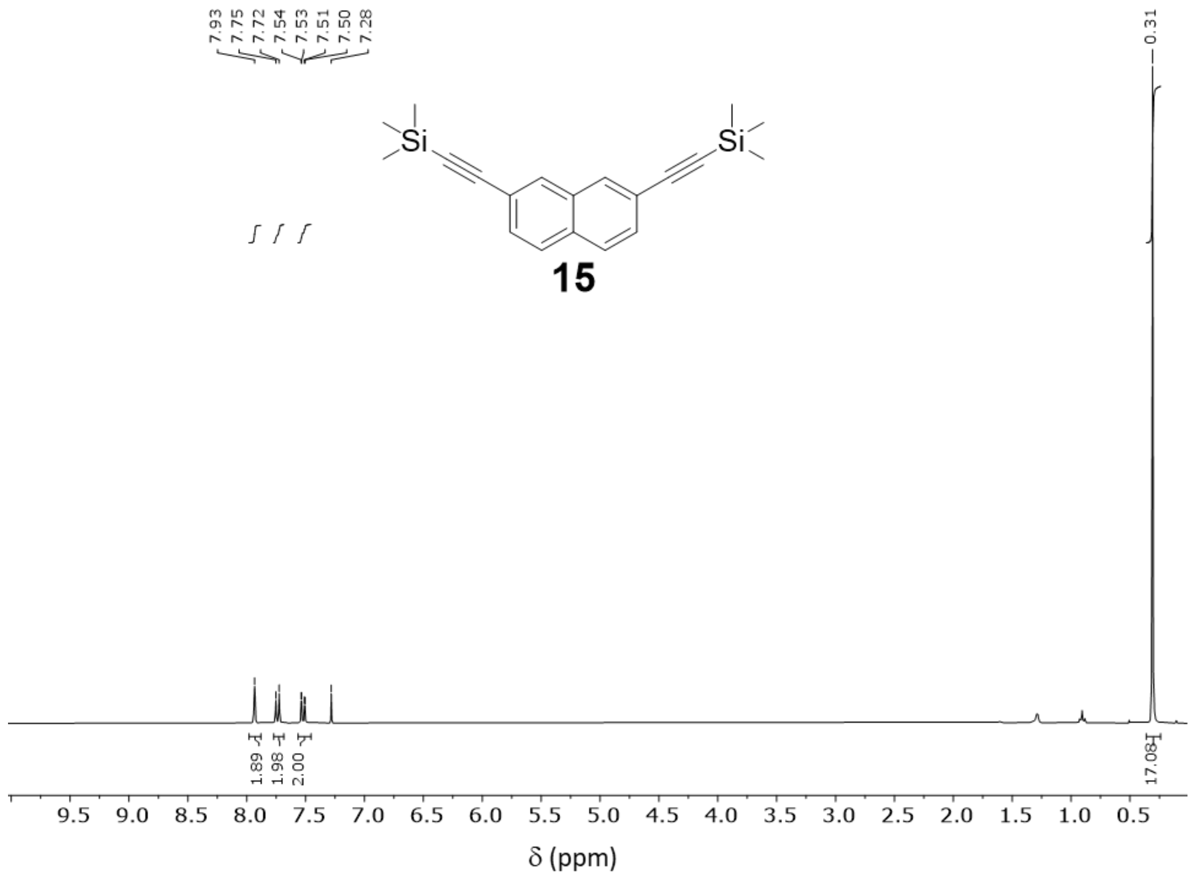

Figure S19. *^{1}H NMR spectrum of compound* ***15*** *(300 MHz, 25°C, $CDCl_3$).*

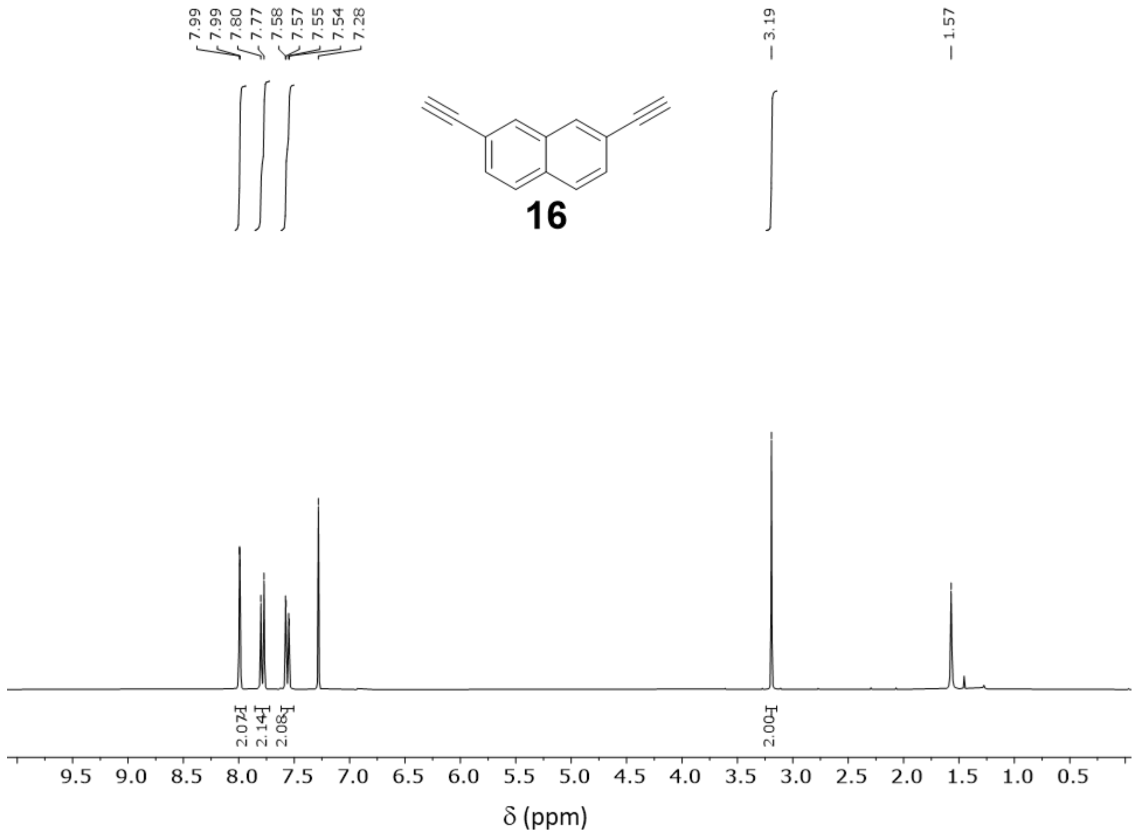

Figure S20. *^{1}H NMR spectrum of compound* ***16*** *(300 MHz, 25°C, $CDCl_3$).*

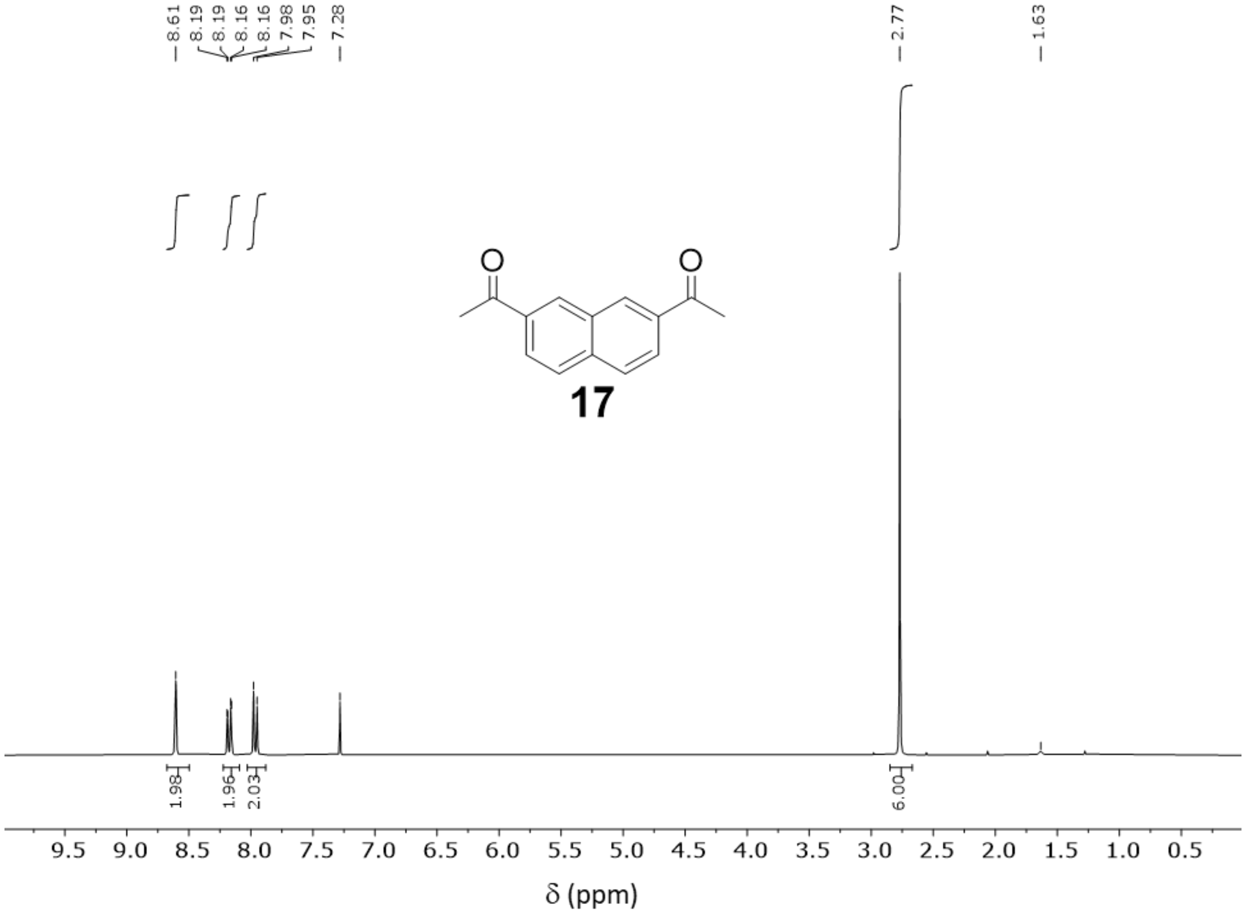

Figure S21. *^{1}H NMR spectrum of compound* ***17*** *(300 MHz, 25°C, $CDCl_3$).*

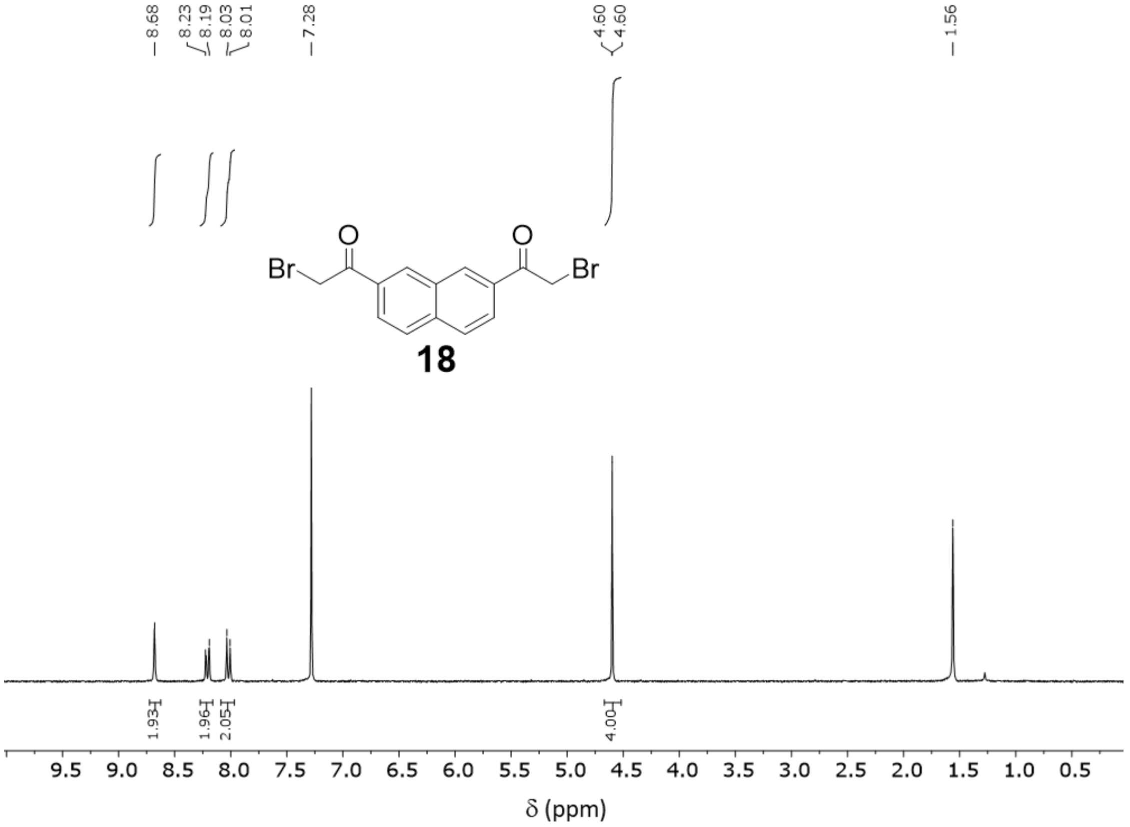

Figure S22. *^{1}H NMR spectrum of compound **18** (400 MHz, 25°C, $CDCl_3$).*

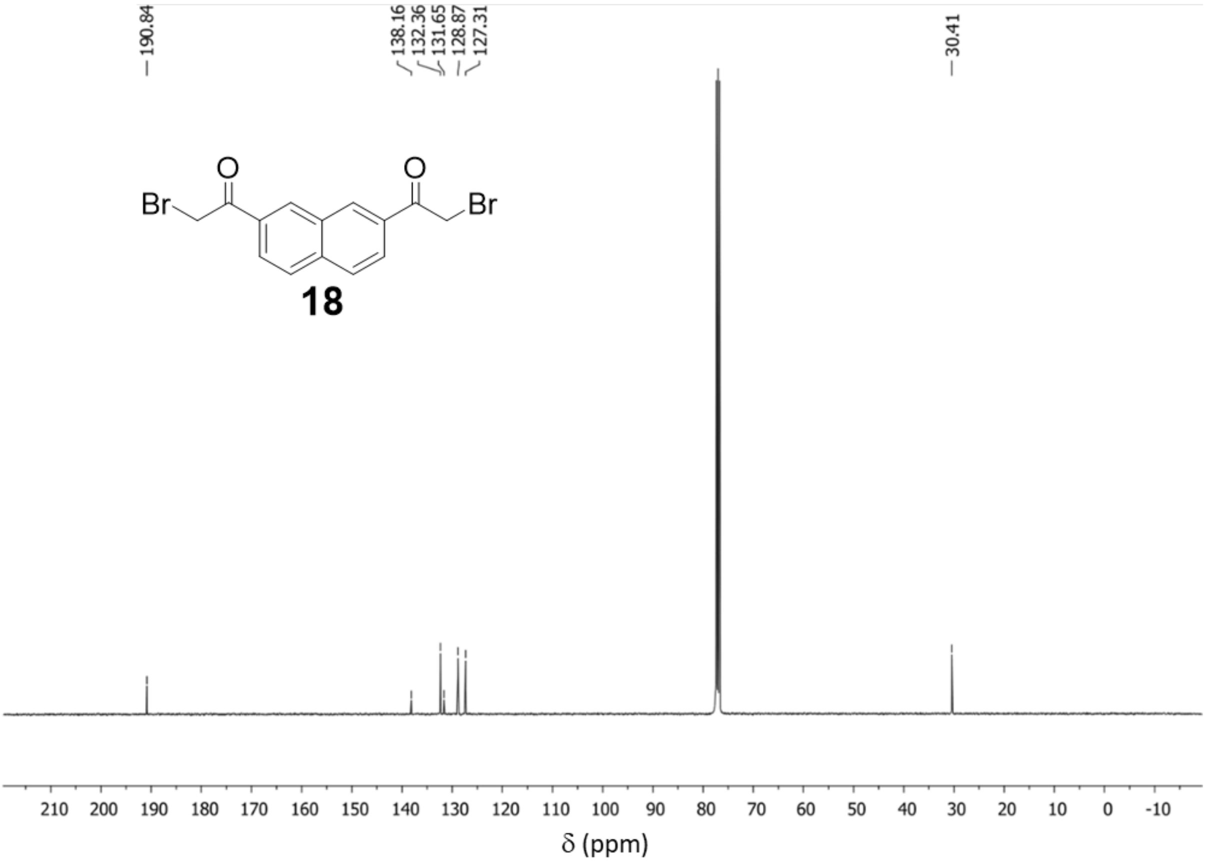

Figure S23. *^{13}C NMR spectrum of compound **18** (101 MHz, 25°C, $CDCl_3$).*

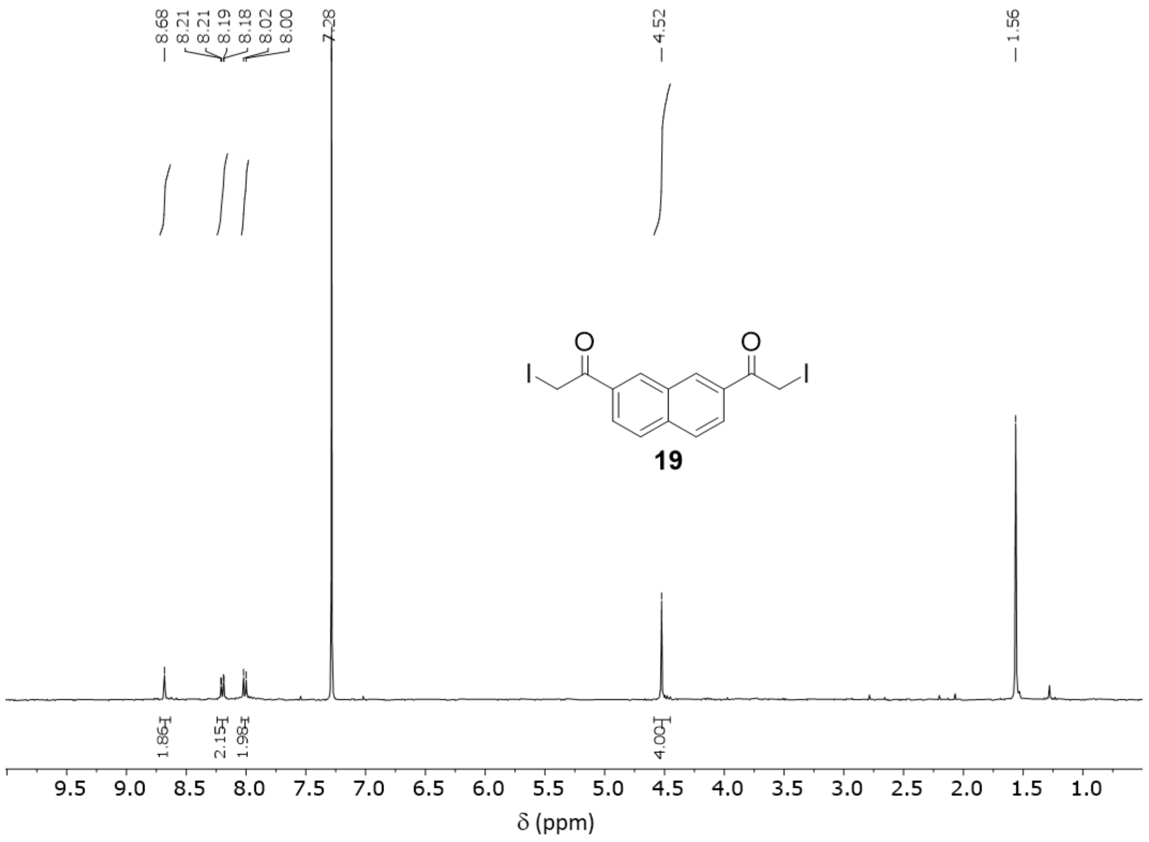

Figure S24. *^{1}H NMR spectrum of compound* ***19*** *(300 MHz, 25°C, CDCl₃).*

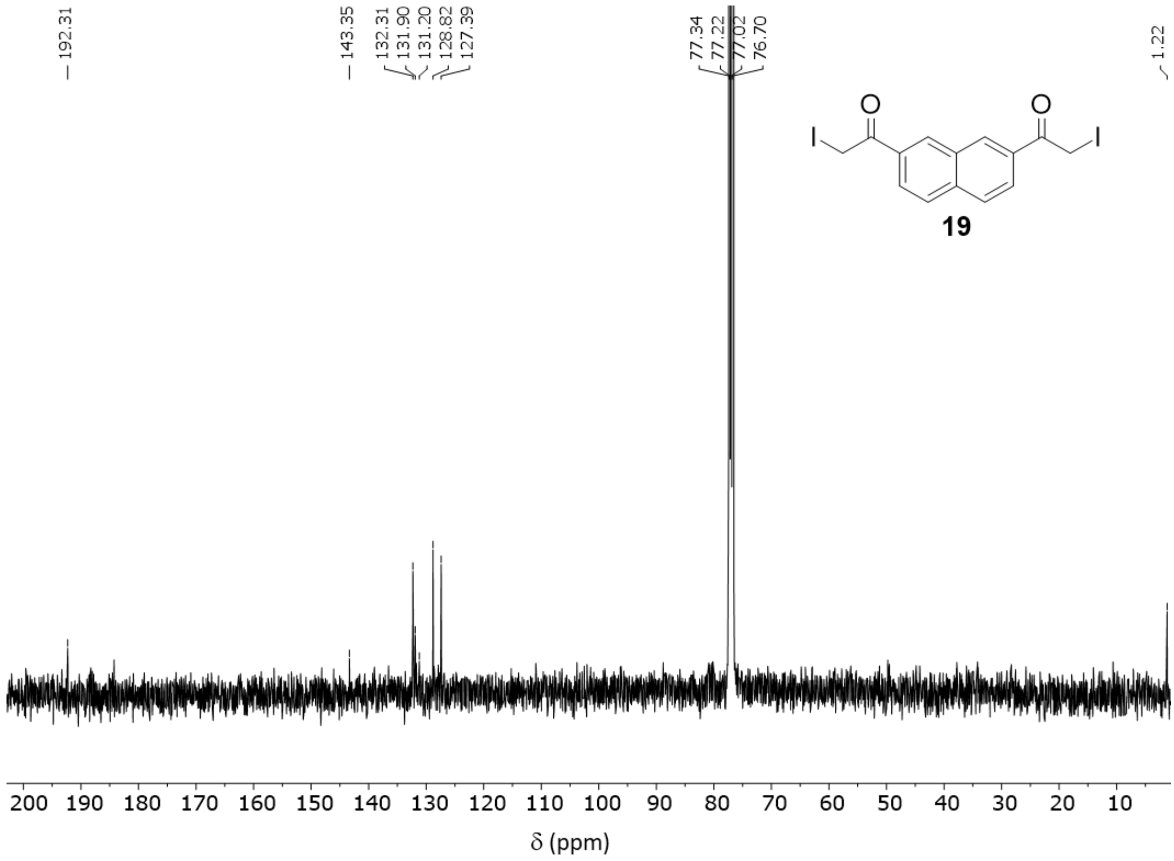

Figure S25. *^{13}C NMR spectrum of compound* ***19*** *(101 MHz, 25°C, CDCl₃). The formation of dark precipitates during this experiment suggested compound degradation, which hindered the acquisition of higher-quality spectra.*

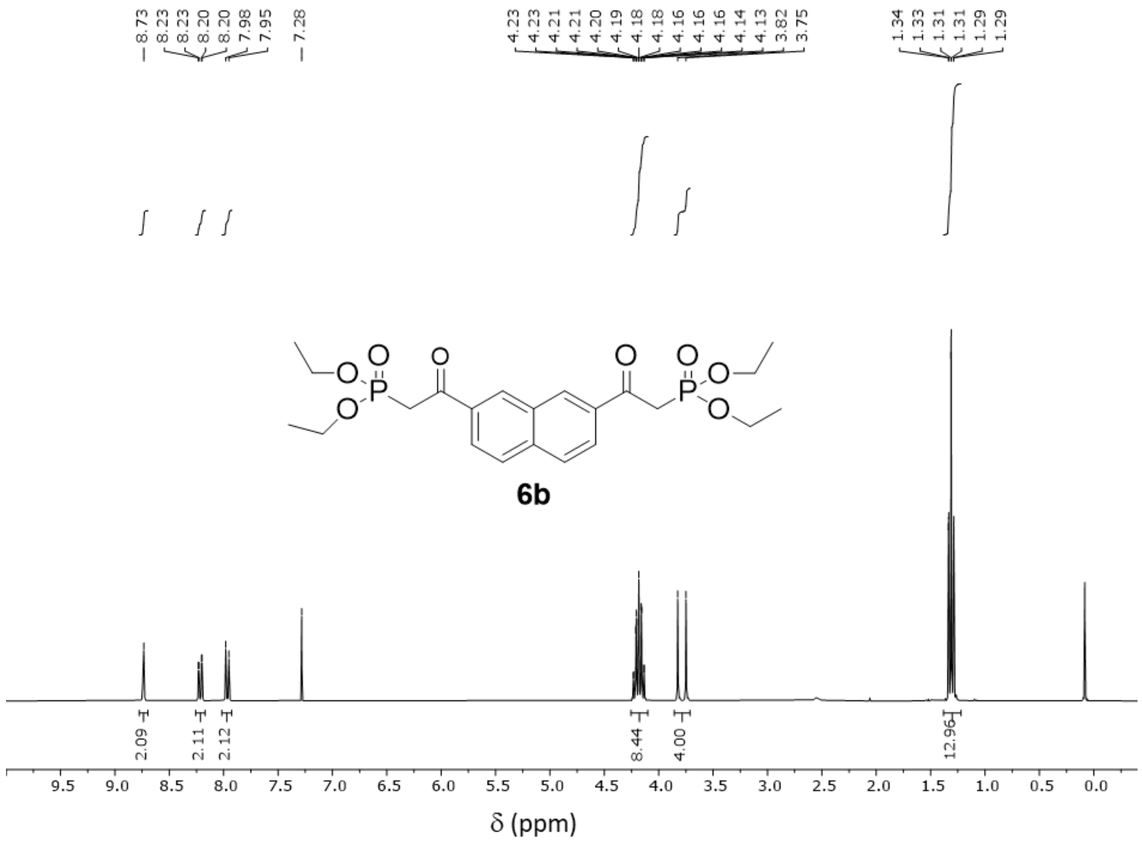

Figure S26. *^{1}H NMR spectrum of compound* ***6b*** *(400 MHz, 25°C, $CDCl_3$).*

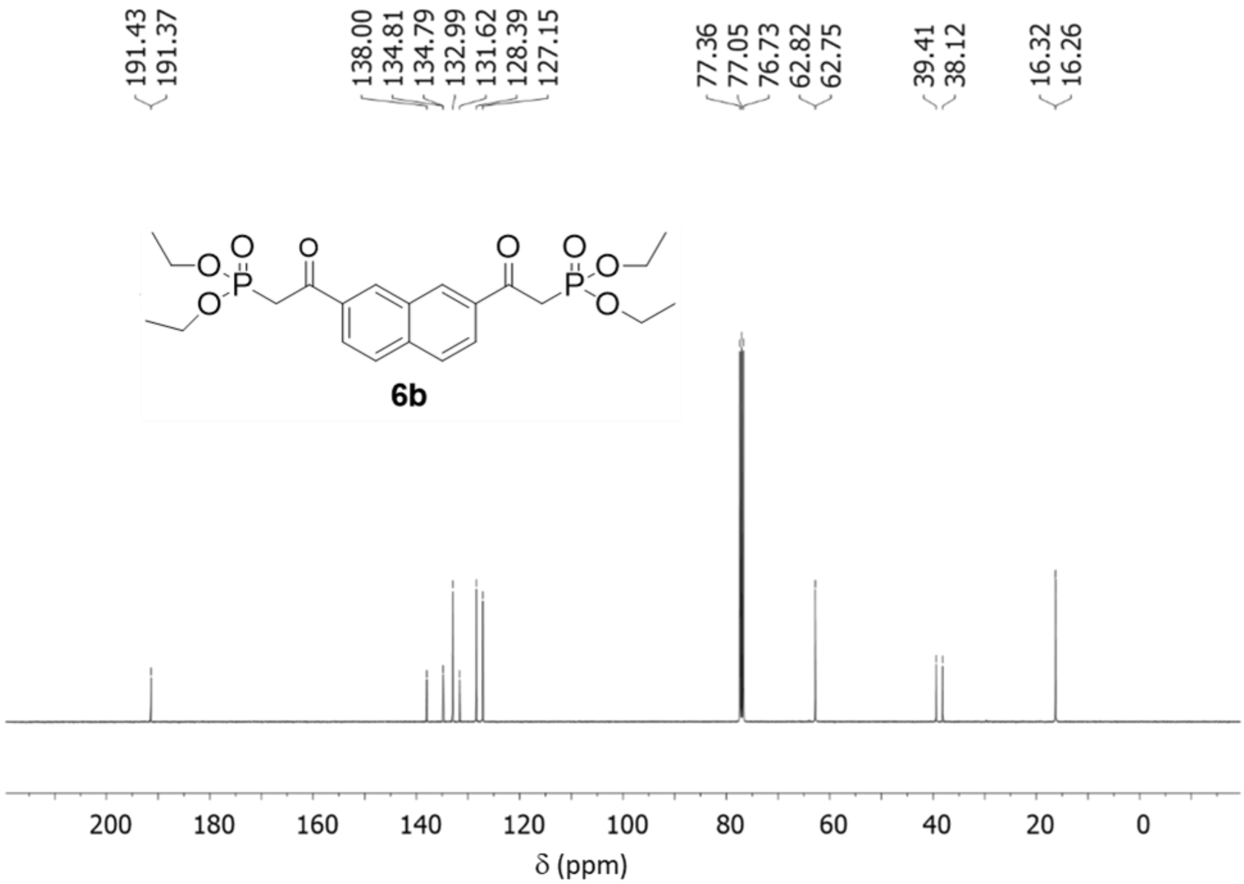

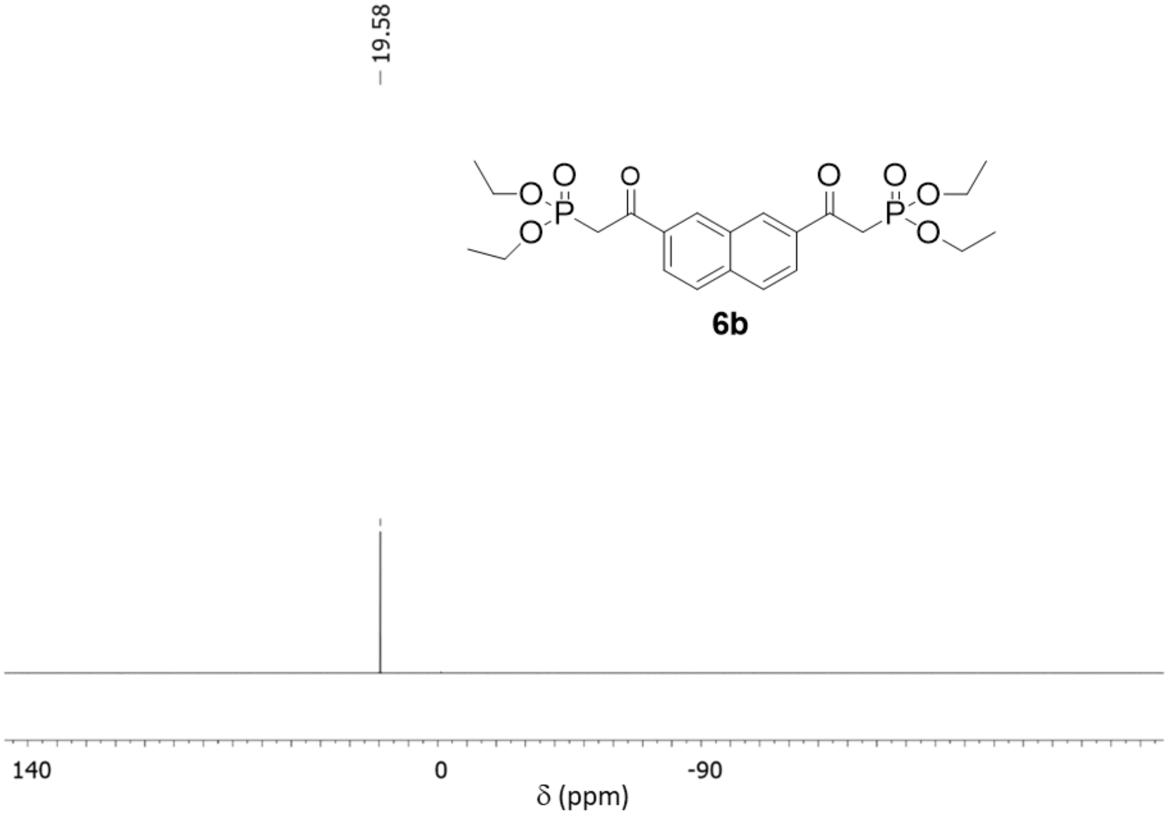

Figure S27. *^{13}C (top) and ^{31}P NMR (bottom) of compound **6b** (101 and 162 MHz, respectively, 25°C, $CDCl_3$).*

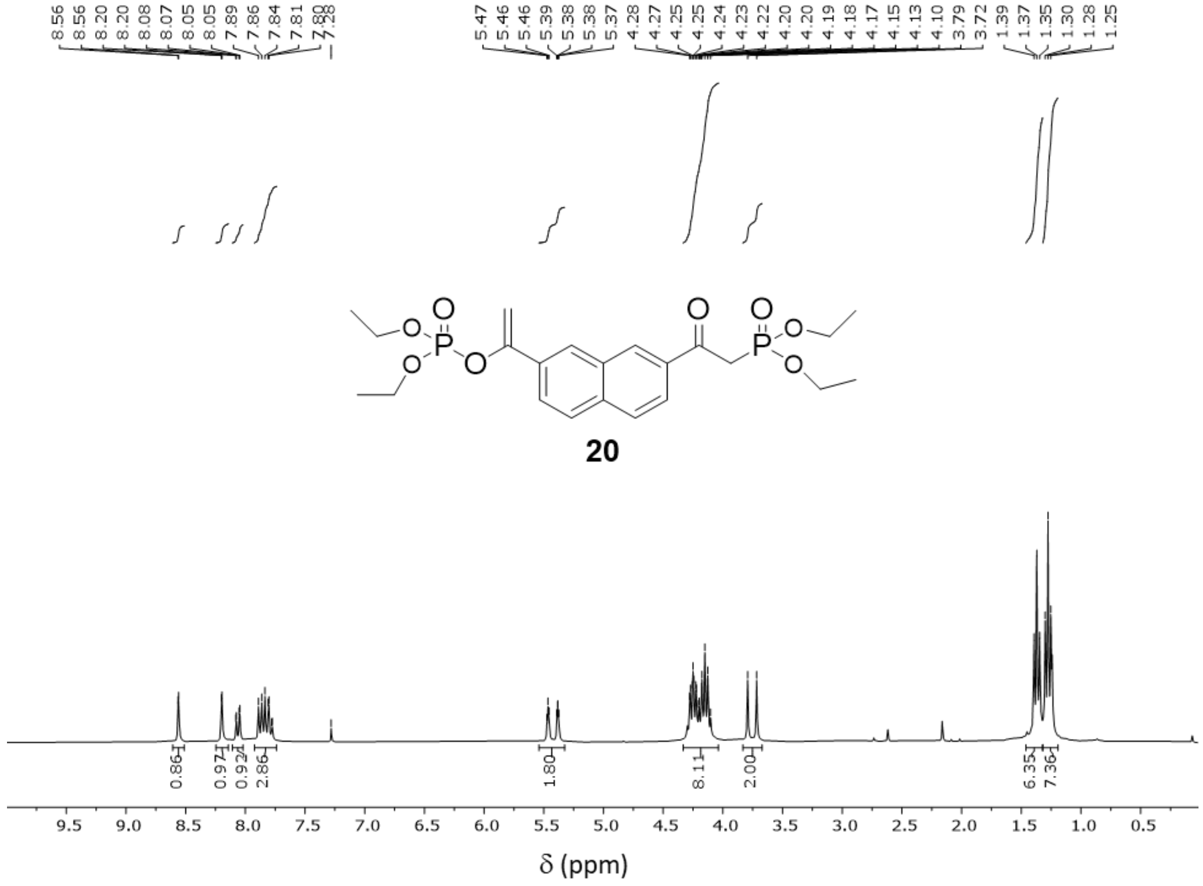

Figure S28. *^{1}H NMR spectrum of compound **20** (300 MHz, 25°C, $CDCl_3$).*

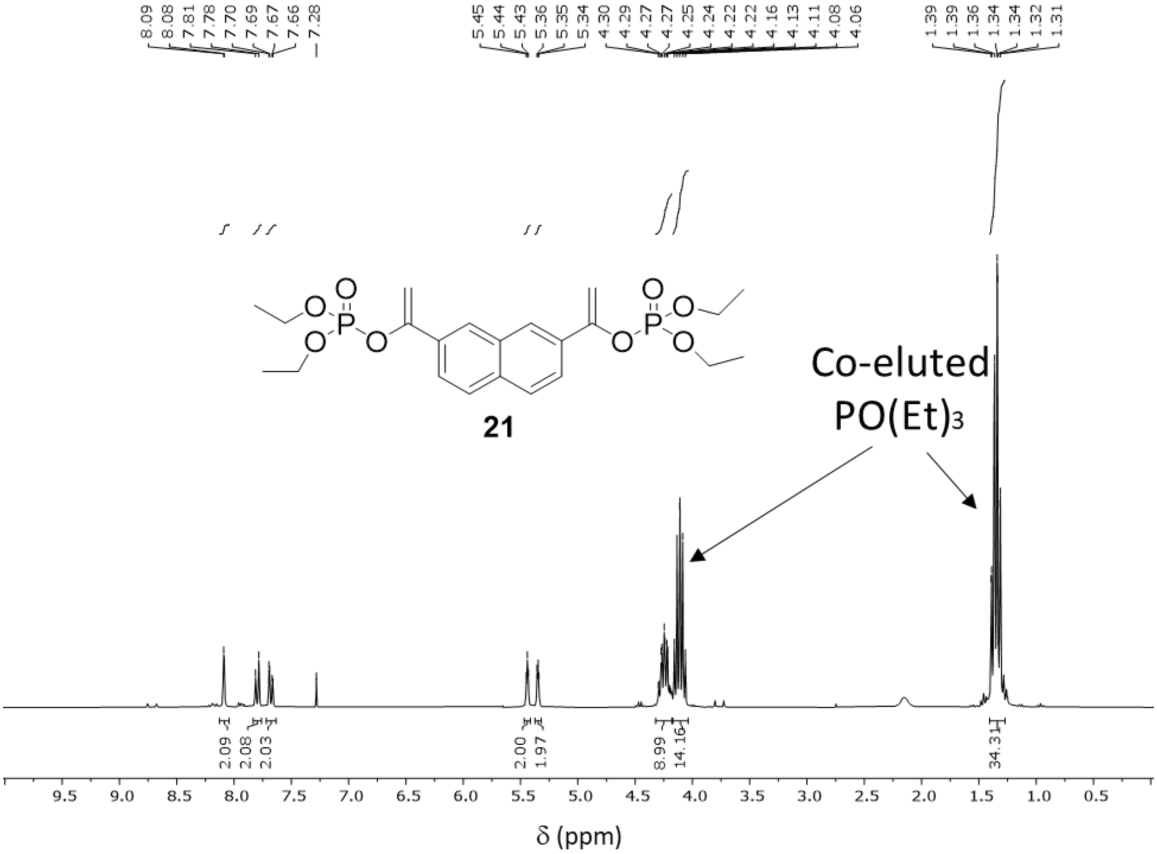

Figure S29. *^{1}H NMR spectrum of compound **21** (400 MHz, 25°C, $CDCl_3$).*

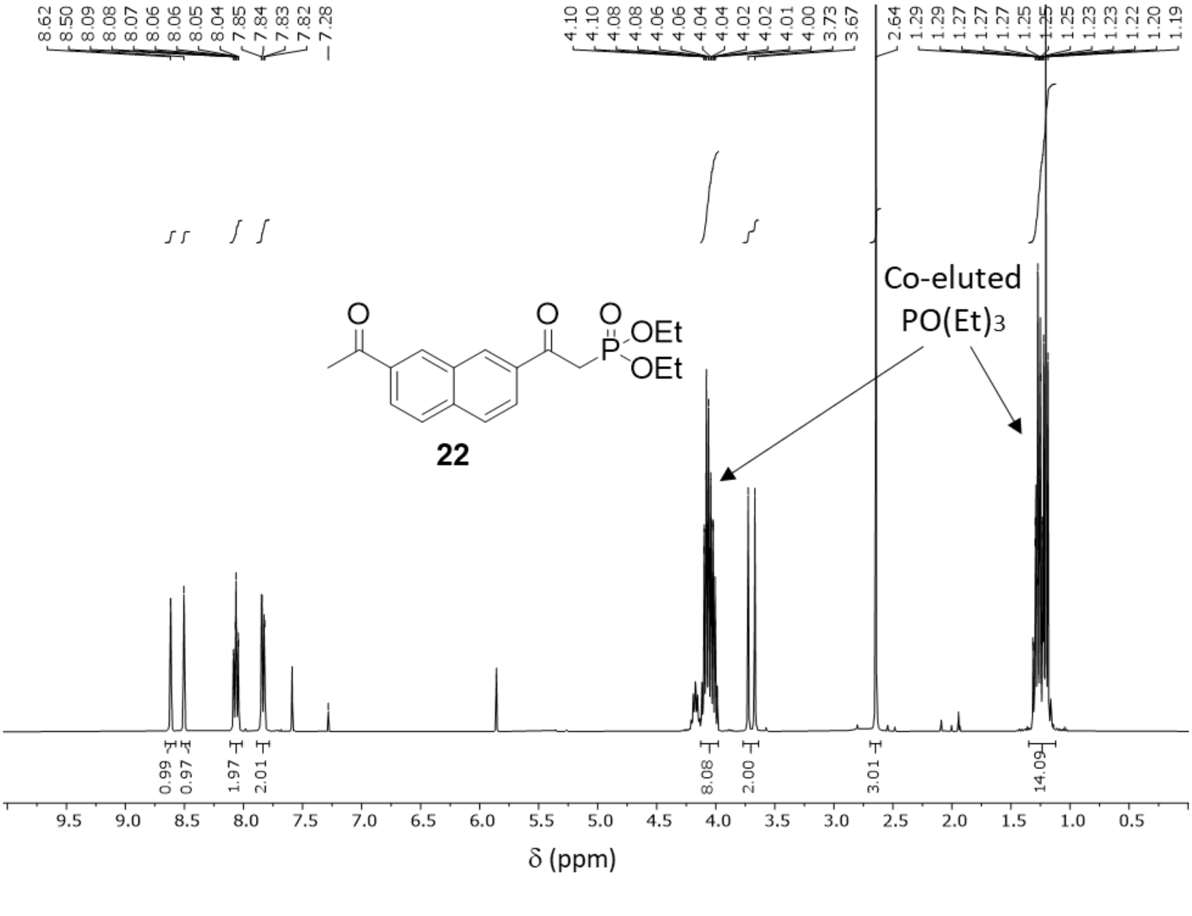

Figure S30: *^{1}H NMR spectrum of compound **22** (400 MHz, 25°C, $CDCl_3$).*

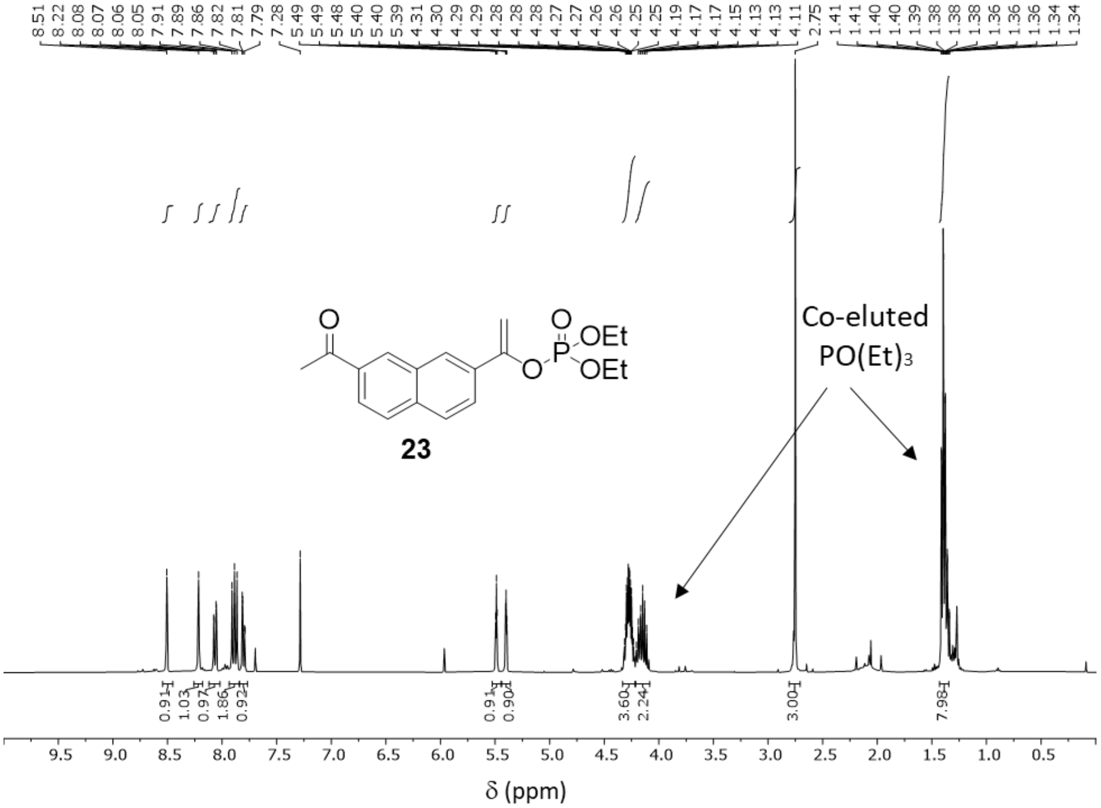

Figure S 31: *^{1}H NMR spectrum of compound* **23** *(400 MHz, 25°C, $CDCl_3$).*

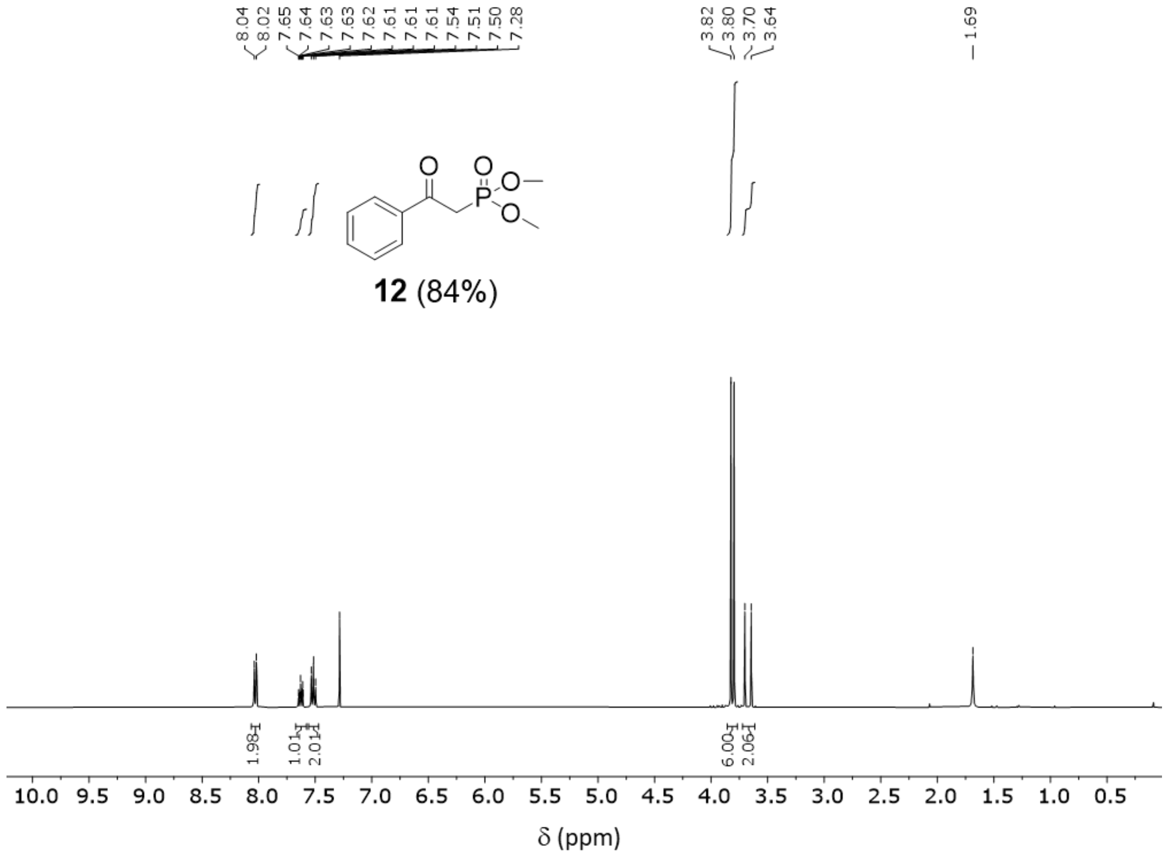

Figure S 32. *^{1}H NMR spectrum of compound* **12** *(400 MHz, 25°C, $CDCl_3$).*

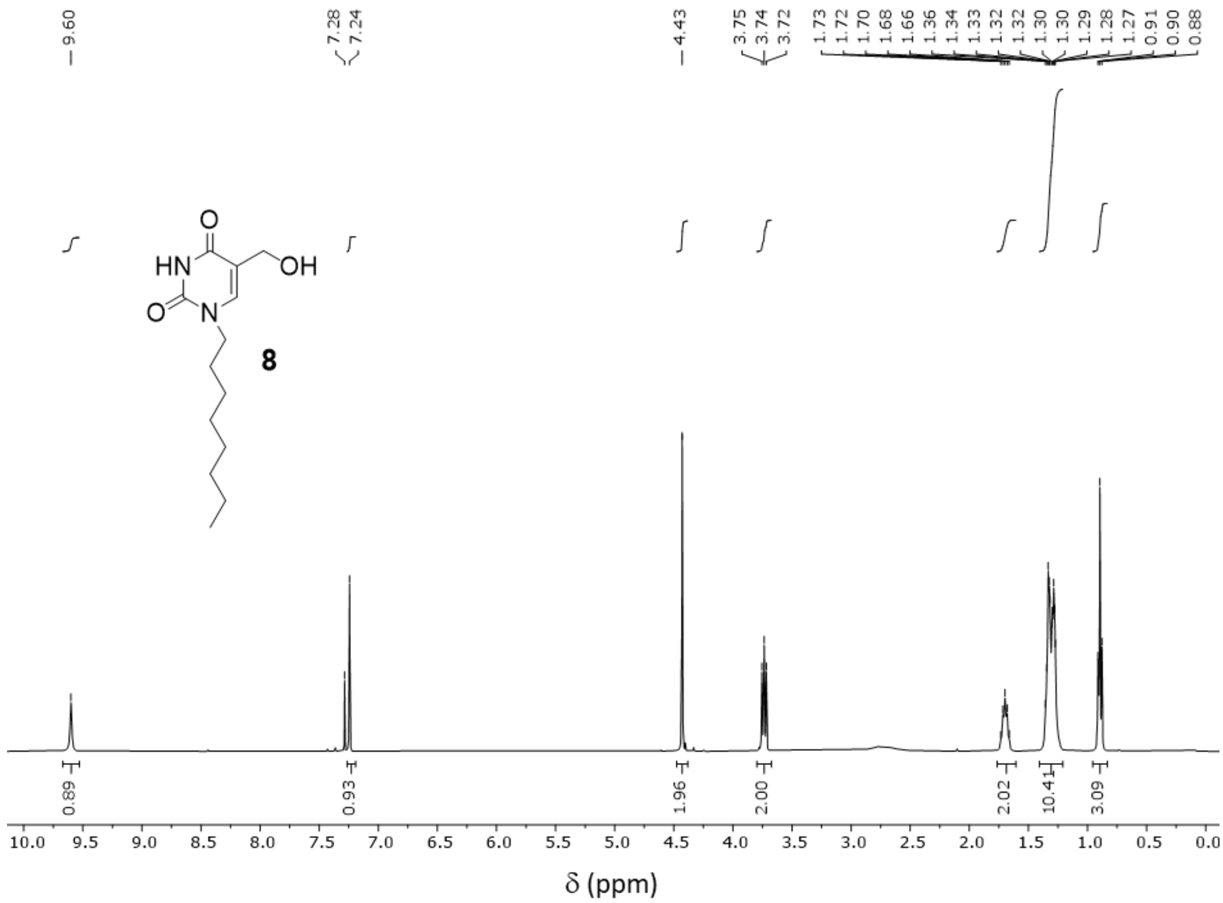

Figure S 33. *^{1}H NMR spectrum of compound **8** (400 MHz, 25°C, CDCl₃).*

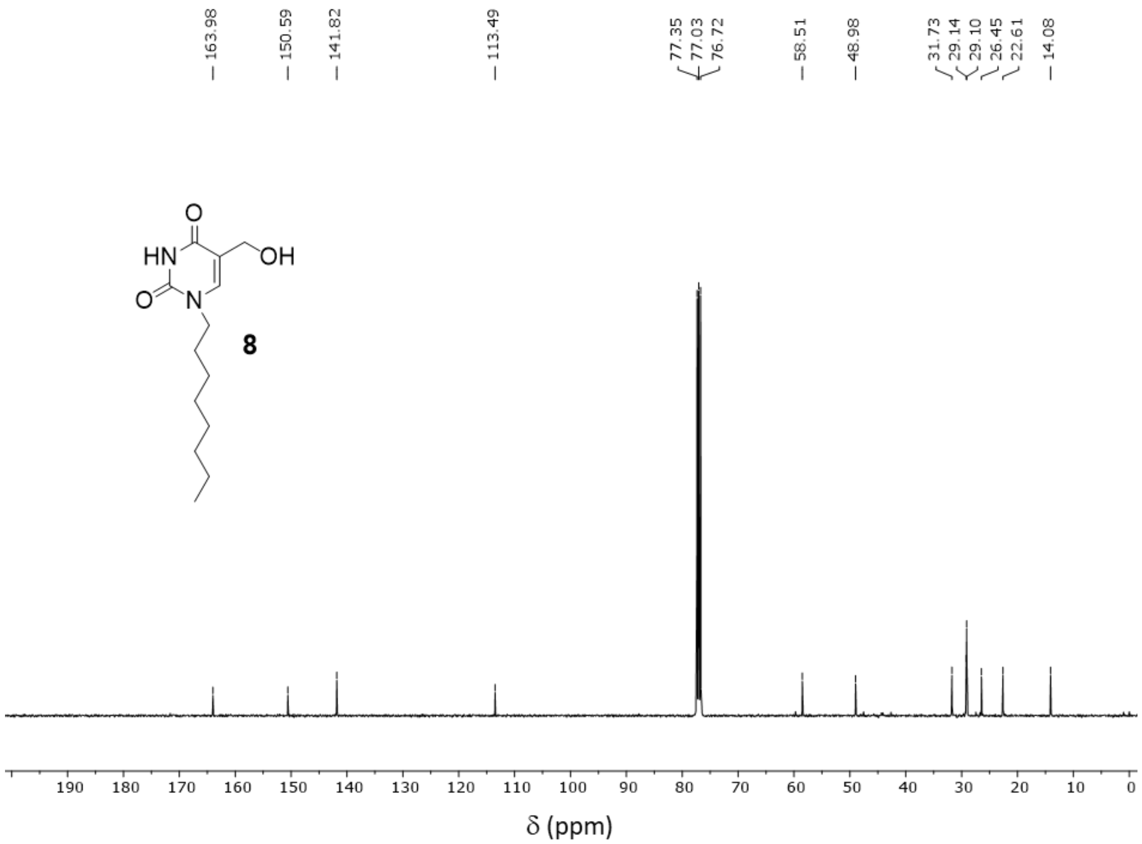

Figure S34. *^{13}C NMR spectrum of compound **8** (101 MHz, 25°C, $CDCl_3$).*

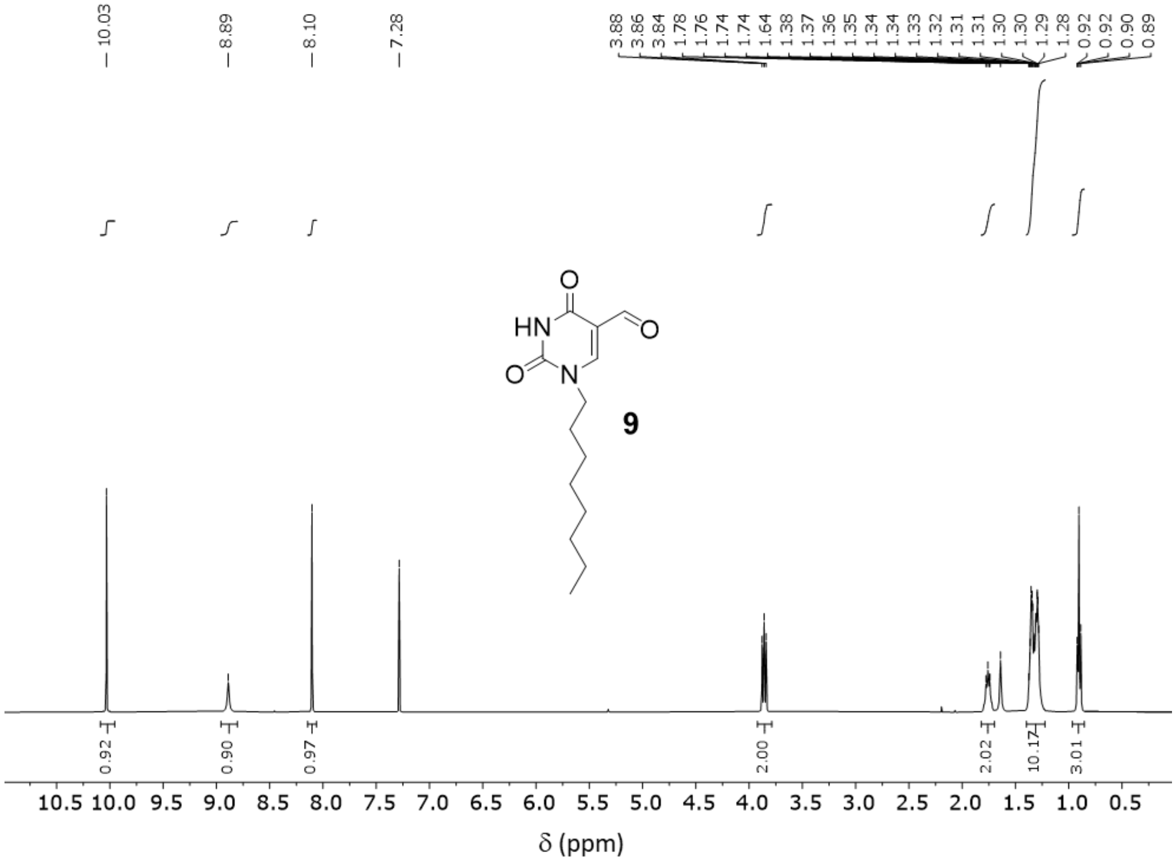

Figure S 35. *^{1}H NMR spectrum of compound **9** (400 MHz, 25°C, $CDCl_3$).*

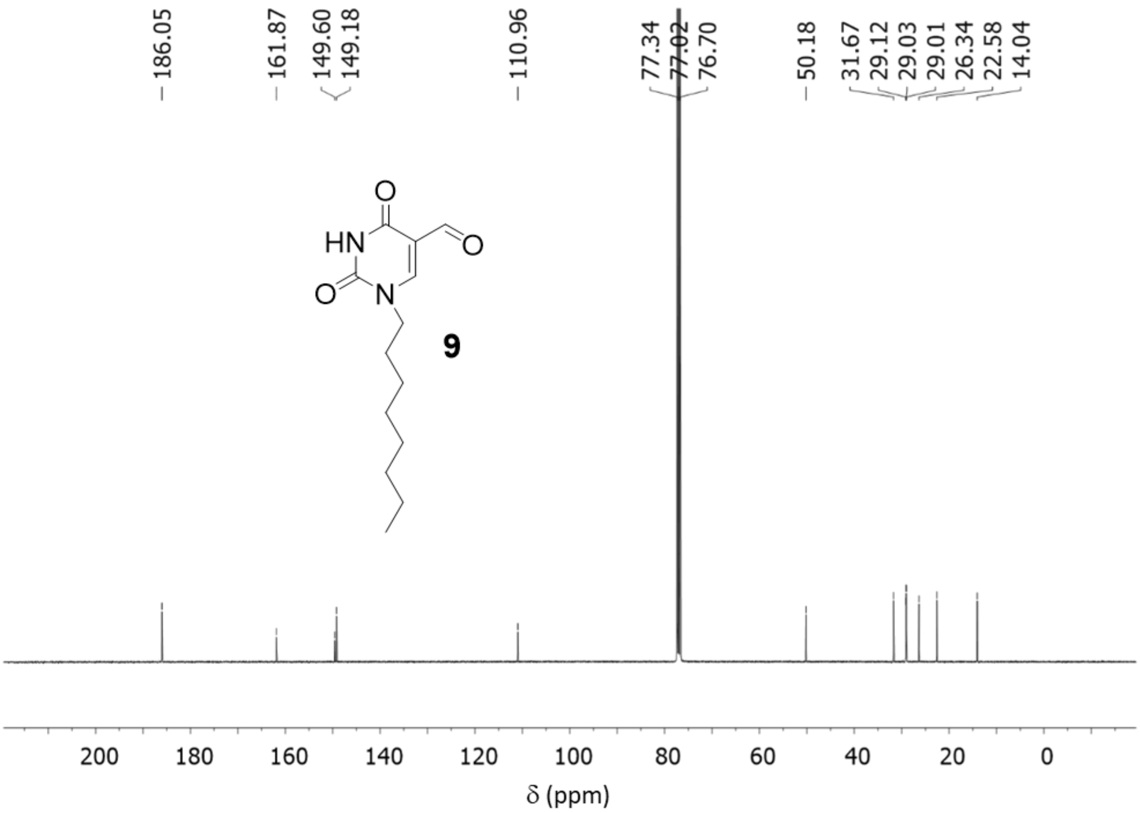

Figure S36. *^{13}C NMR spectrum of compound* ***9*** *(101 MHz, 25°C, $CDCl_3$).*

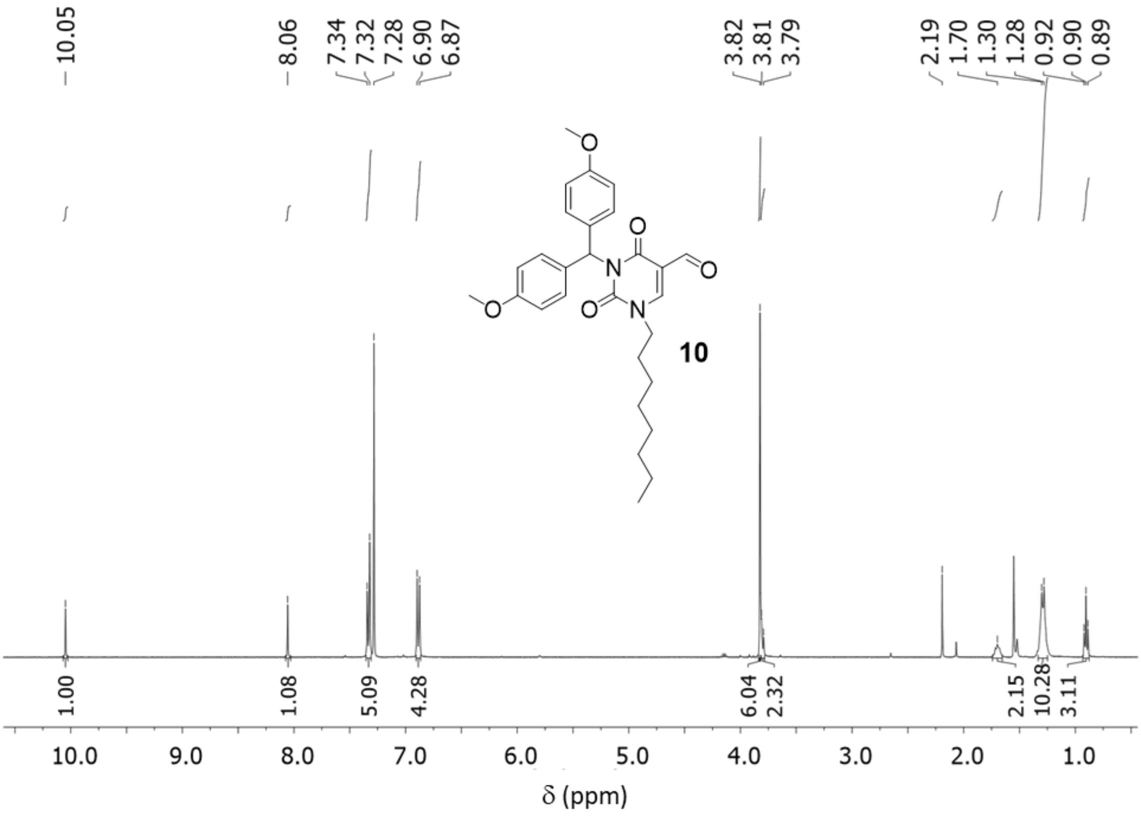

Figure S37. *^{1}H NMR spectrum of compound* ***10*** *(400 MHz, 25°C, $CDCl_3$).*

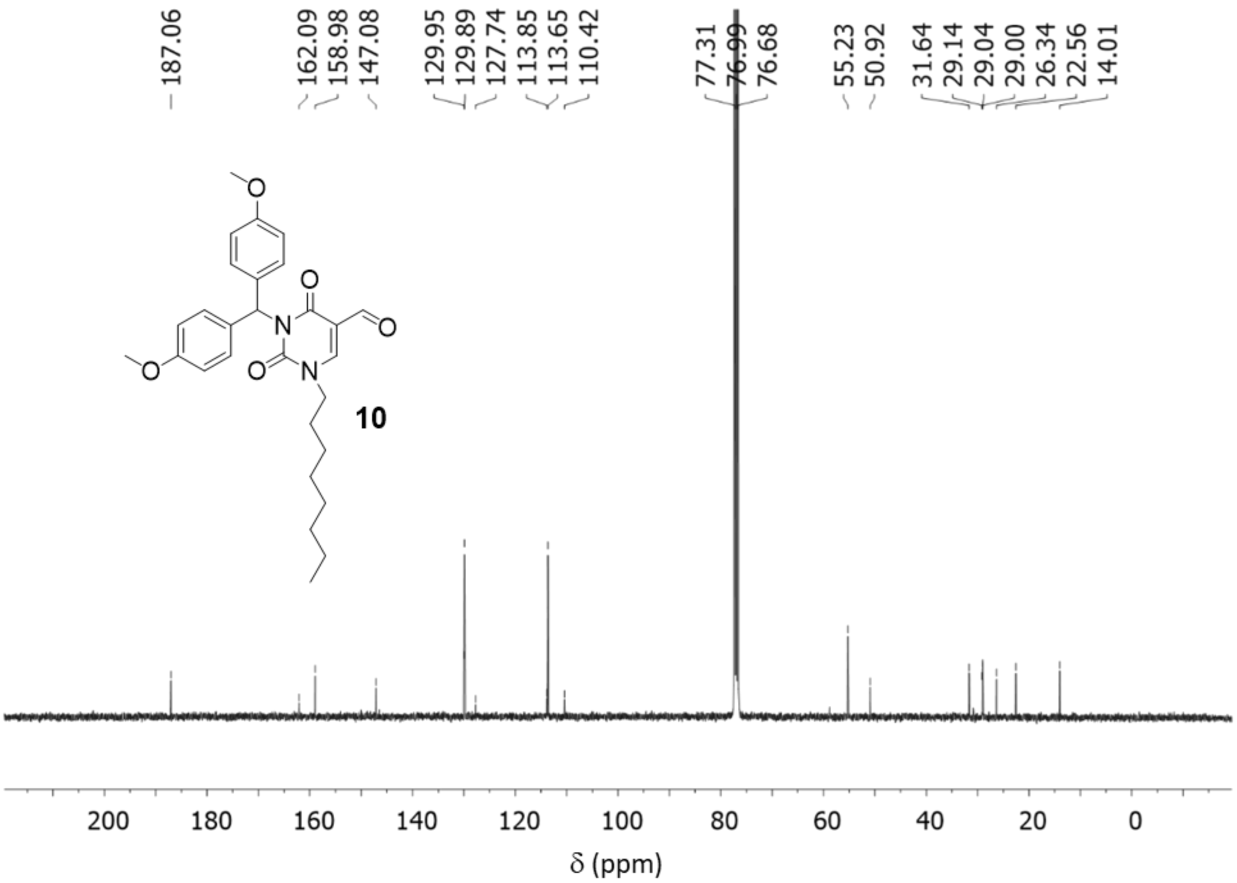

Figure S38. *^{13}C NMR spectrum of compound **10** (101 MHz, 25°C, CDCl$_3$).*

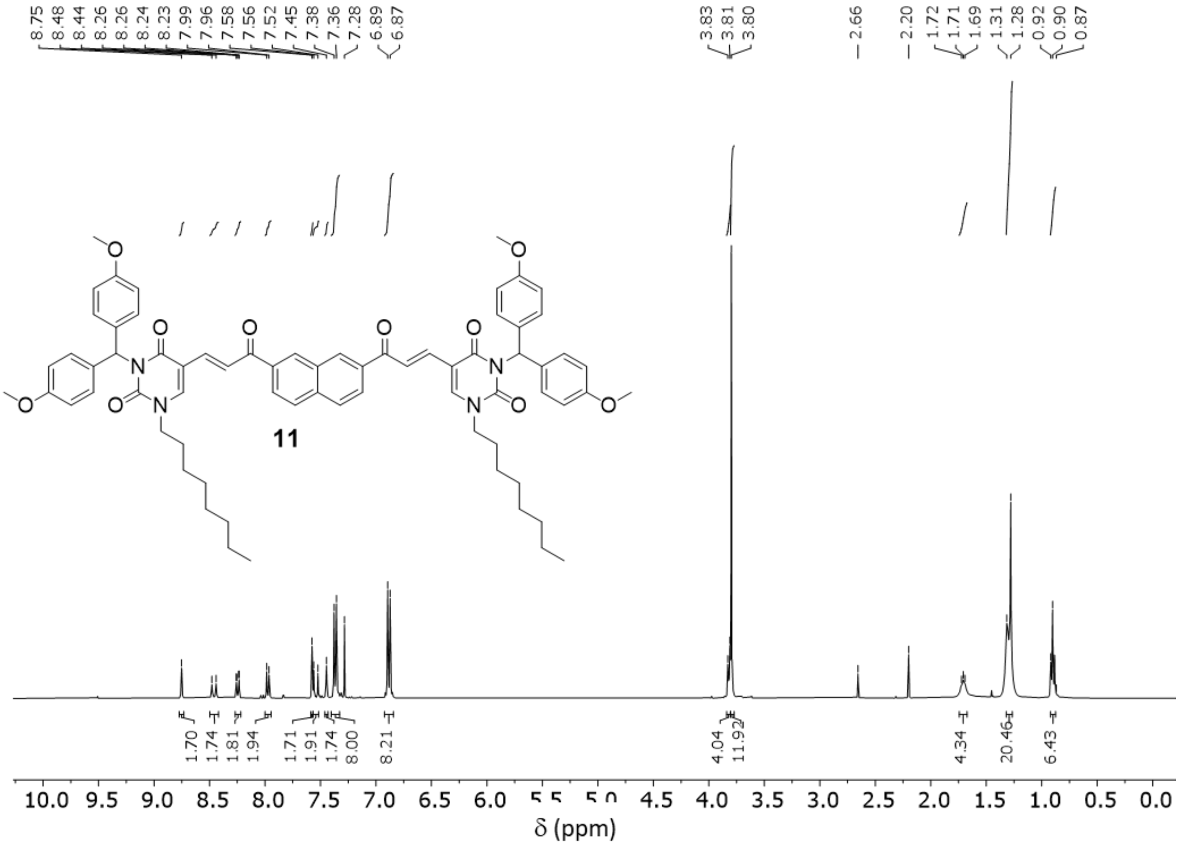

Figure S39. *^{1}H NMR spectrum of compound **11** (400 MHz, 25°C, CDCl$_3$).*

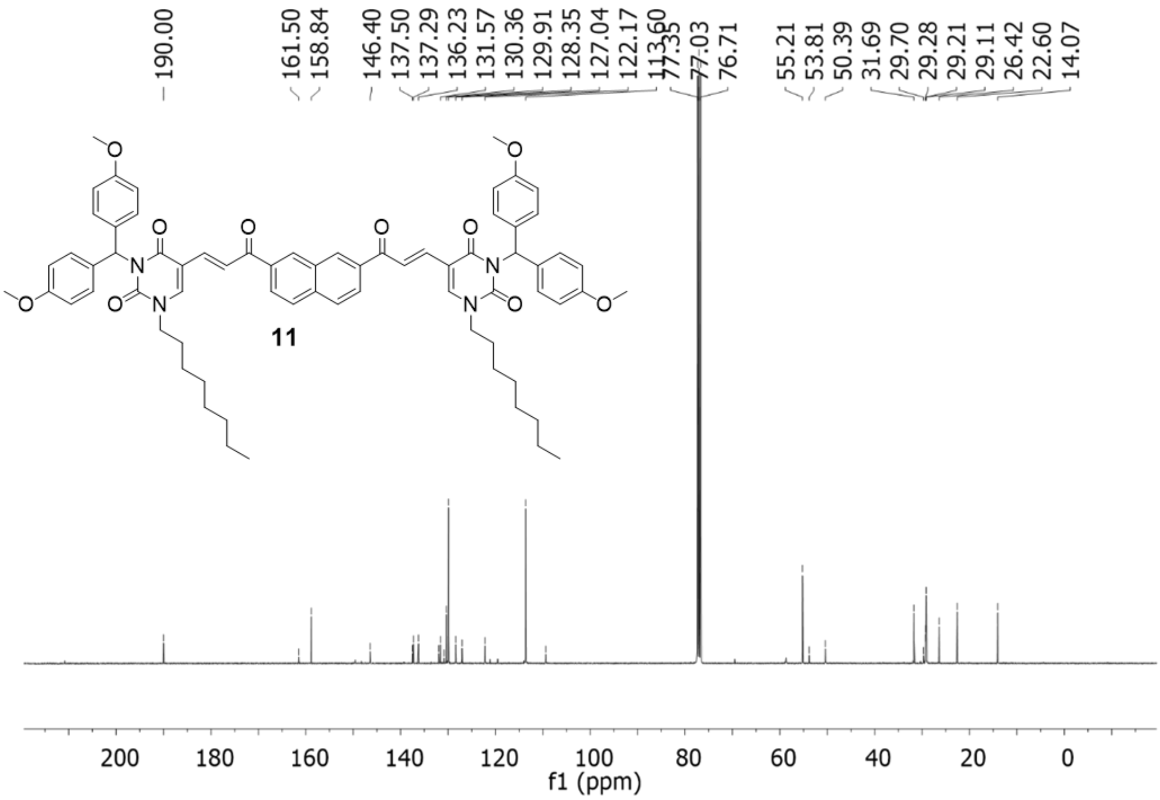

Figure S40. *^{13}C NMR spectrum of compound **11** (101 MHz, 25°C, CDCl$_3$).*

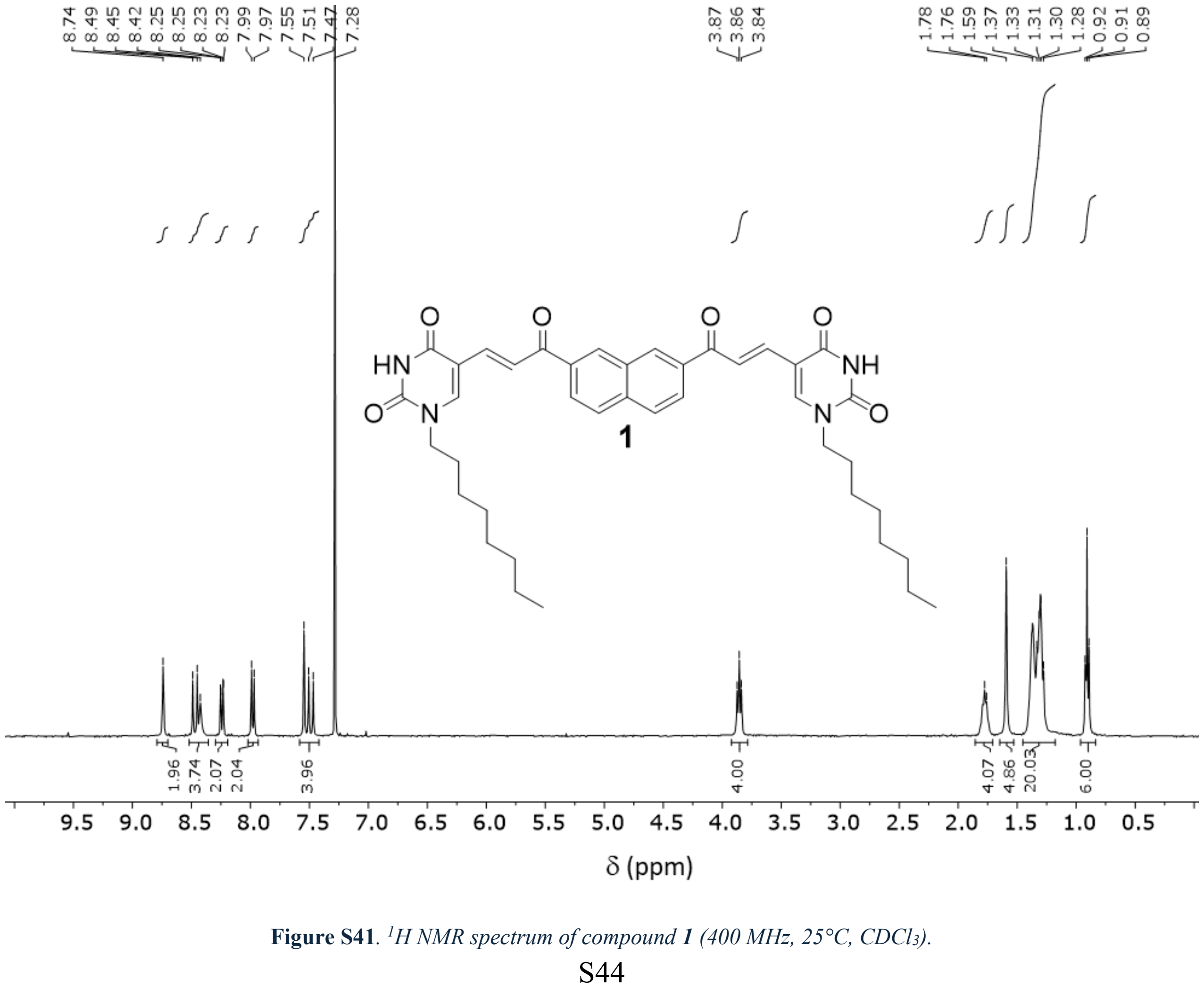

Figure S41. *^{1}H NMR spectrum of compound **1** (400 MHz, 25°C, CDCl$_3$).*

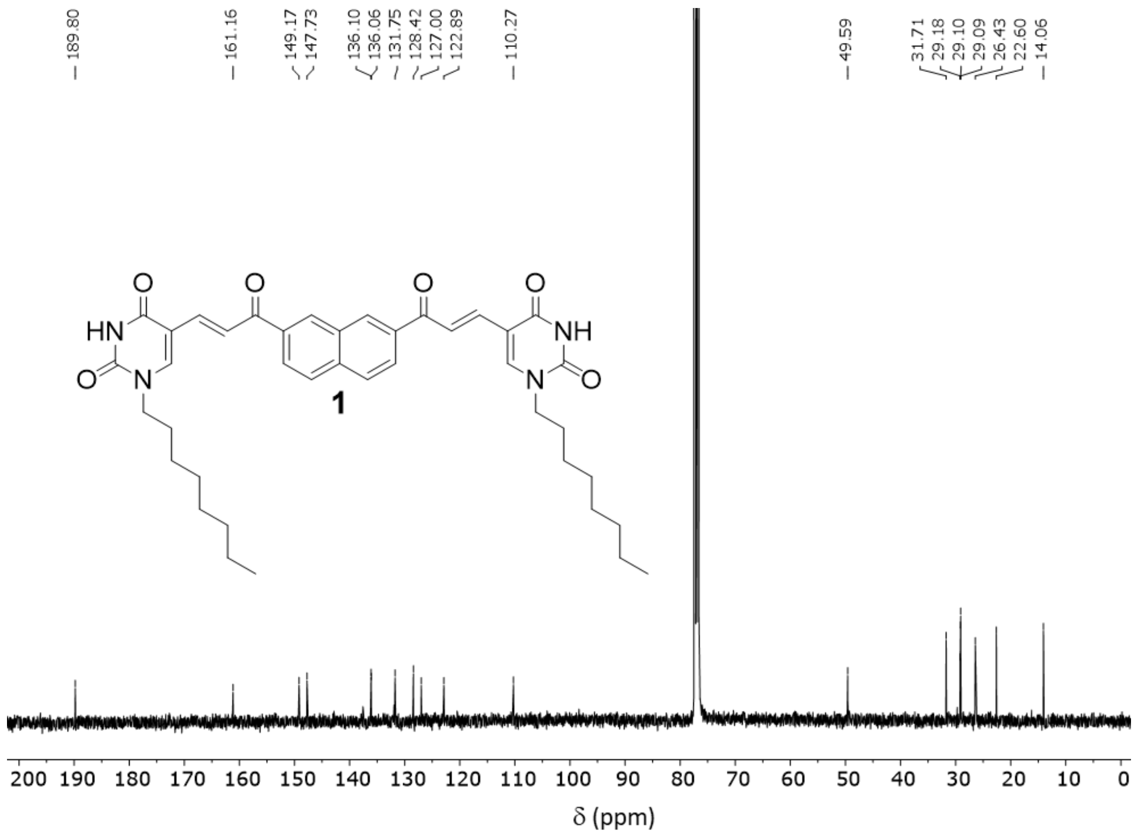

Figure S42. *^{13}C NMR spectrum of compound **1** (101 MHz, 25°C, $CDCl_3$).*

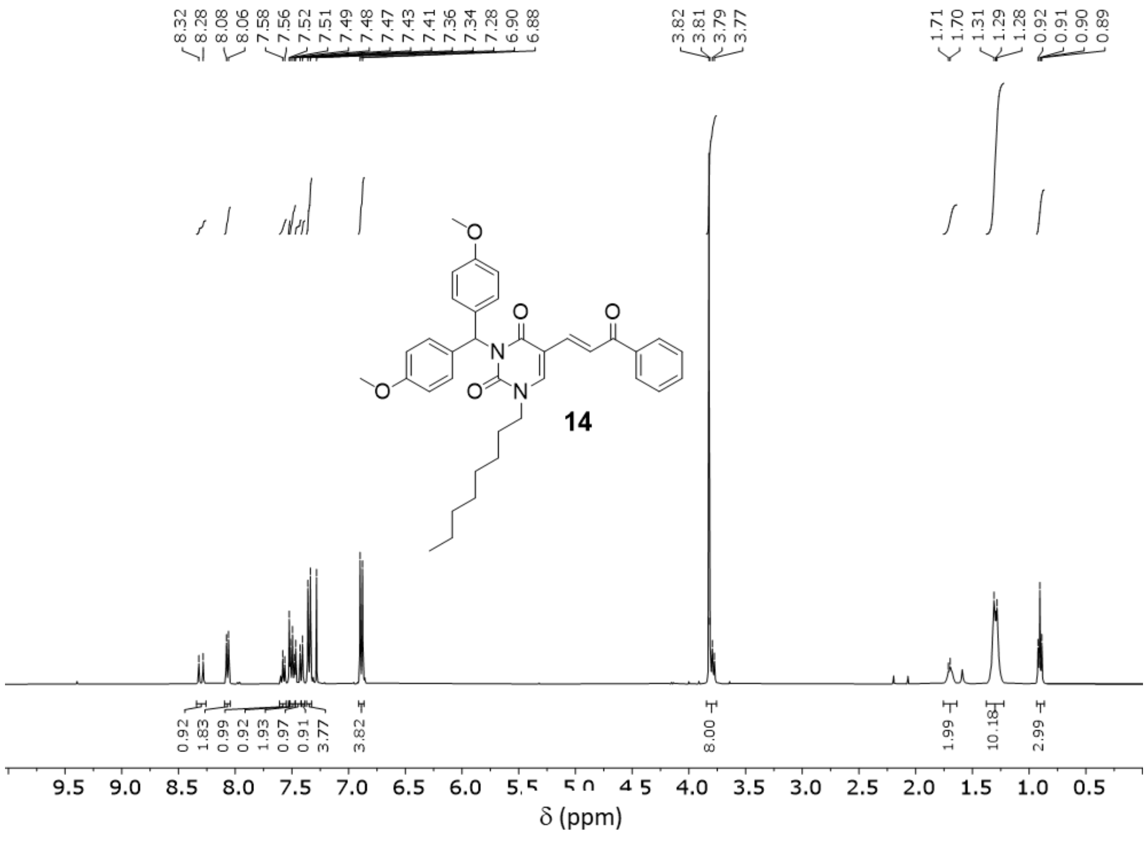

Figure S 43. *^{1}H NMR spectrum of compound **14** (400 MHz, 25°C, $CDCl_3$).*

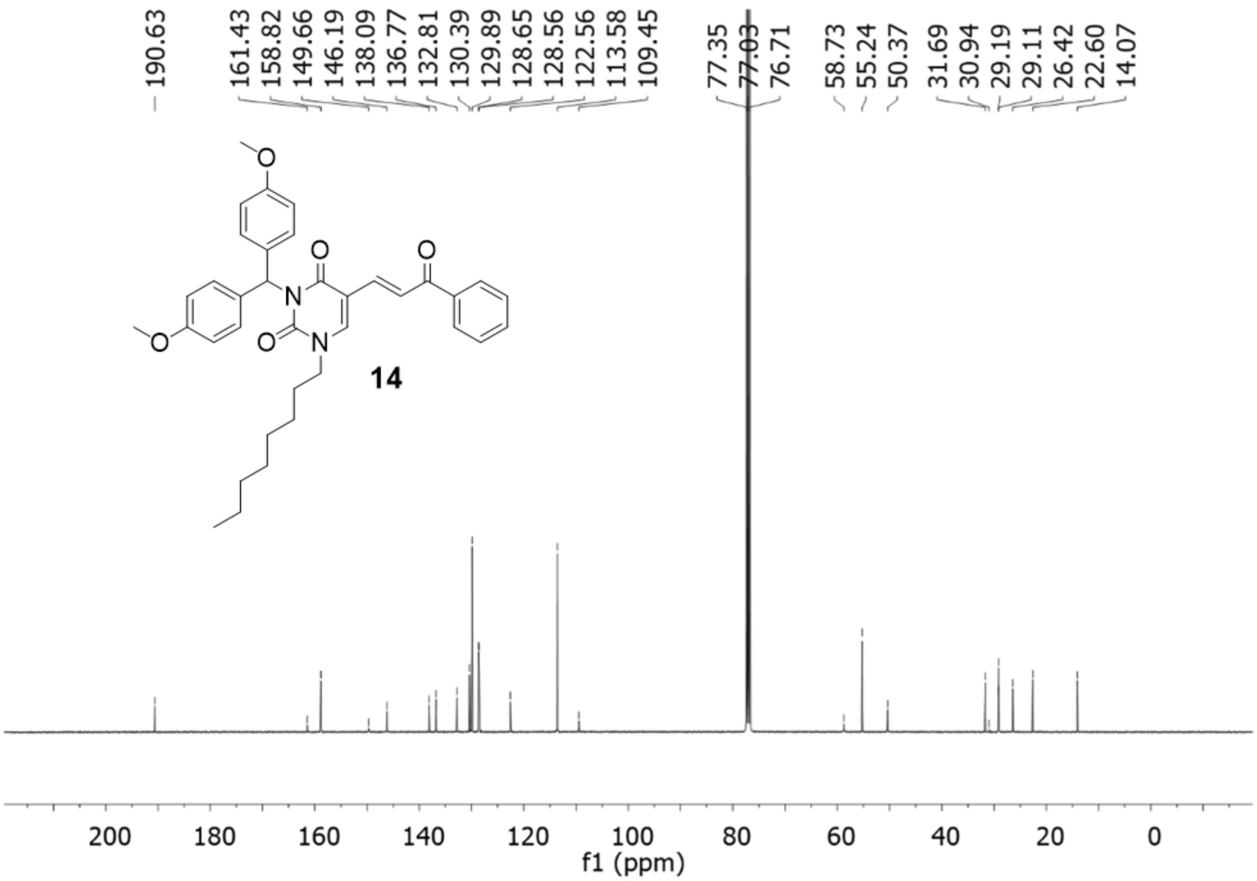

Figure S44. *^{13}C NMR spectrum of compound* ***14*** *(101 MHz, 25°C, $CDCl_3$).*

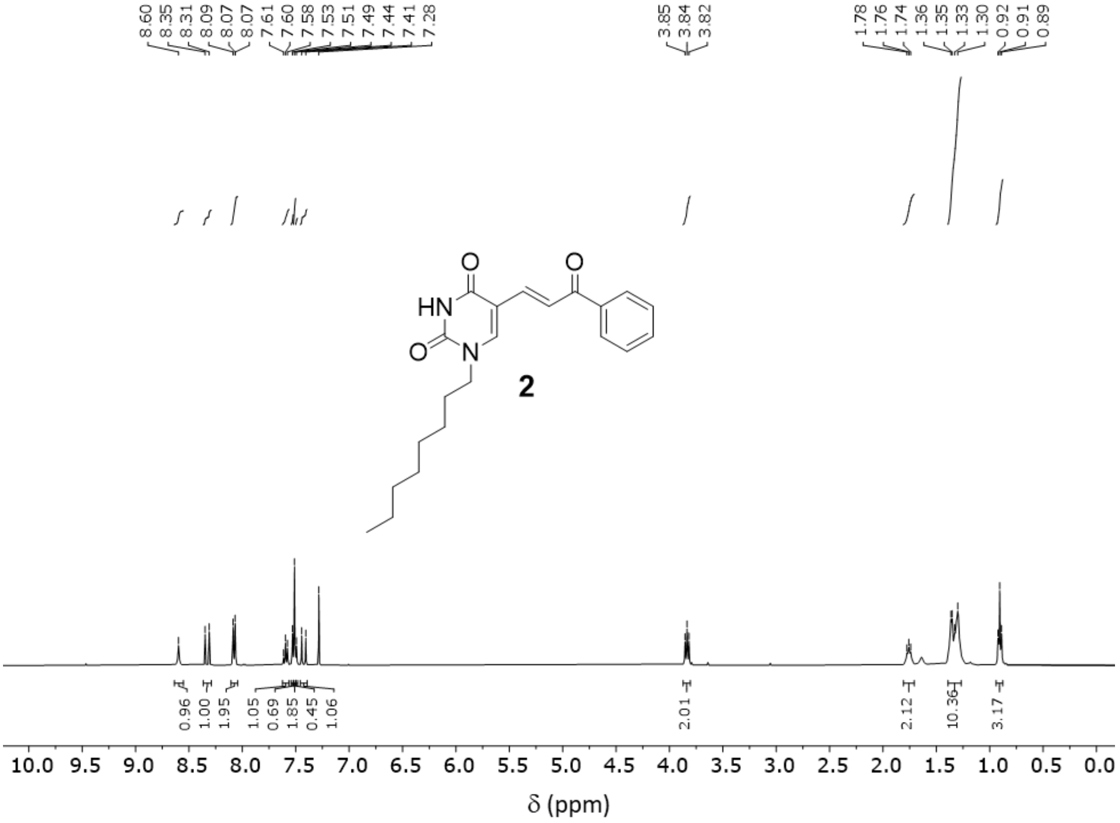

Figure S45: *^{1}H NMR spectrum of compound* ***2*** *(400 MHz, 25°C, $CDCl_3$).*

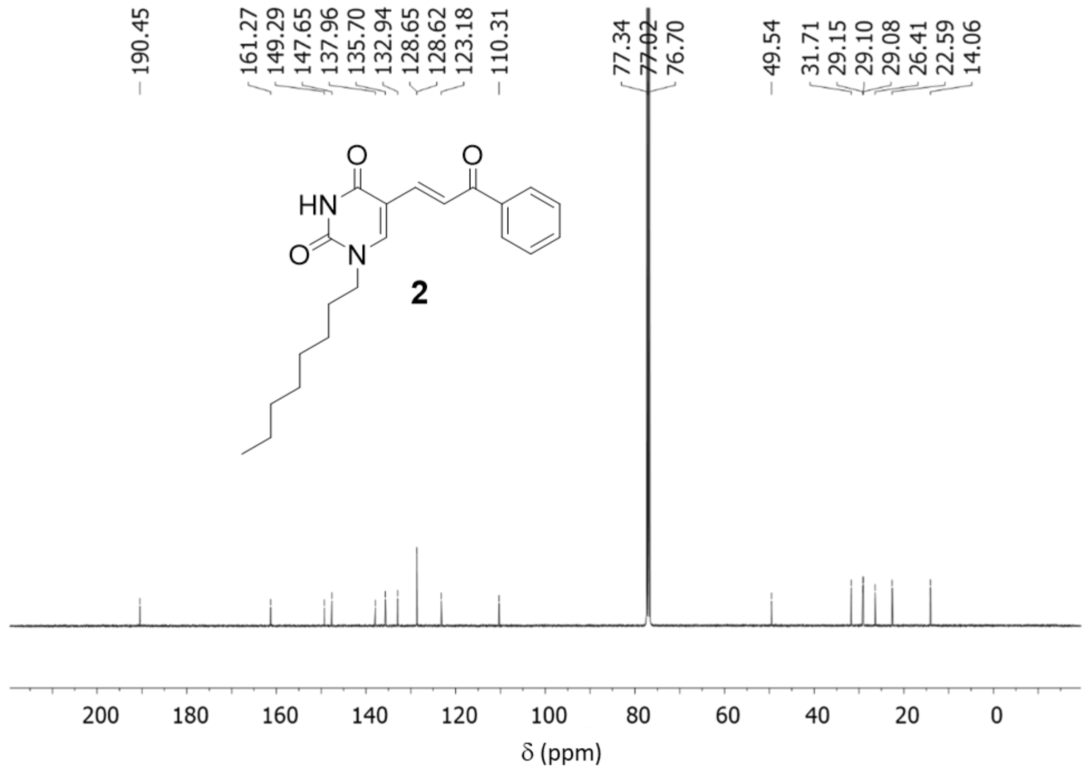

Figure S46. *^{13}C NMR spectrum of compound **2** (101 MHz, 25°C, $CDCl_3$).*

7. References

(1) Accetta, A.; Corradini, R.; Sforza, S.; Tedeschi, T.; Brognara, E.; Borgatti, M.; Gambari, R.; Marchelli, R. New Uracil Dimers Showing Erythroid Differentiation Inducing Activities. *J Med Chem* **2009**, *52* (1), 87–94. https://doi.org/10.1021/jm800982q.

(2) Accetta, A. *Molecular Engineering of PNA Using Modified Uracil Derivatives and Porphyrins - PhD Thesis*; University of Parma: Parma, IT, 2010.

(3) Verona, D. M. *Modified PNA Design and Synthesis: A Novel Approach Using Molecular Dynamics and Metadynamics - PhD Thesis*; University of Parma: Parma, IT, 2016.

(4) Betts, L.; Josey, J. A.; Veal, J. M.; Jordan, S. R. A Nucleic Acid Triple Helix Formed by a Peptide Nucleic Acid-DNA Complex. *Science (1979)* **1995**, *270* (5243), 1838–1841. https://doi.org/10.1126/science.270.5243.1838.

(5) Itahara, T.; Fujii, Y.; Tada, M. Oxidation of Thymines by Peroxosulfate Ions in Water. *Journal of Organic Chemistry* **1988**, *53* (15), 3421–3424. https://doi.org/10.1021/jo00250a003.

(6) King, A.; Doepner, A.; Turton, D.; Ciobota, D. M.; Da Pieve, C.; Wong Te Fong, A. C.; Kramer-Marek, G.; Chung, Y. L.; Smith, G. Radiosynthesis of the Anticancer Nucleoside Analogue Trifluridine Using an Automated 18F-Trifluoromethylation Procedure. *Org Biomol Chem* **2018**, *16* (16), 2986–2996. https://doi.org/10.1039/c8ob00432c.

(7) Mahajan, T. R.; Ytre-Arne, M. E.; StrØm-Andersen, P.; Dalhus, B.; Gundersen, L. L. Synthetic Routes to N-9 Alkylated 8-Oxoguanines; Weak Inhibitors of the Human DNA Glycosylase OGG1. *Molecules* **2015**, *20* (9), 15944–15965. https://doi.org/10.3390/molecules200915944.

(8) Pothukanuri, S.; Pianowski, Z.; Winssinger, N. Expanding the Scope and Orthogonality of PNA Synthesis. *European J Org Chem* **2008**, No. 18, 3141–3148. https://doi.org/10.1002/ejoc.200800141.

(9) Roselló, Y.; Benito, M.; Barceló-Oliver, M.; Frontera, A.; Molins, E. 1-Ethyluracil, a New Scaffold for Preparing Multicomponent Forms: Synthesis, Characterization, and Computational Studies. *Cryst Growth Des* **2021**, *21* (9), 4857–4870. https://doi.org/10.1021/acs.cgd.1c00175.

(10) Lombardo, C. M.; Welsh, S. J.; Strauss, S. J.; Dale, A. G.; Todd, A. K.; Nanjunda, R.; Wilson, W. D.; Neidle, S. A Novel Series of G-Quadruplex Ligands with Selectivity for HIF-Expressing Osteosarcoma and Renal Cancer Cell Lines. *Bioorg Med Chem Lett* **2012**, *22* (18), 5984–5988. https://doi.org/10.1016/j.bmcl.2012.07.009.

(11) Yao, Z. J.; Ye, B.; Wu, X. W.; Wang, S.; Wu, L.; Zhang, Z. Y.; Burke, T. R. Structure-Based Design and Synthesis of Small Molecule Protein-Tyrosine Phosphatase 1B Inhibitors. *Bioorg Med Chem* **1998**, *6* (10), 1799–1810. https://doi.org/10.1016/S0968-0896(98)00140-0.

(12) Somu, R. V.; Boshoff, H.; Qiao, C.; Bennett, E. M.; Barry, C. E.; Aldrich, C. C. Rationally-Designed Nucleoside Antibiotics That Inhibit Siderophore Biosynthesis of Mycobacterium Tuberculosis. *J Med Chem* **2006**, *49* (1), 31–34. https://doi.org/10.1021/jm051060o.

(13) Borowitz, I. J.; Firstenberg, S.; Borowitz, G. B.; Schuessler, D. Organophosphorus Chemistry. XVII. Kinetics and Mechanism of the Perkow Reaction. *J Am Chem Soc* **1972**, *94* (5), 1623–1628. https://doi.org/10.1021/ja00760a032.

(14) Bhattacharya, A. K.; Thyagarajan, G. Michaelis-Arbuzov Rearrangement. *Chem Rev* **1981**, *81* (4), 415–430. https://doi.org/10.1021/cr00044a004.

(15) Friesen, R. W.; Blouin, M. Preparation of γ,δ-Unsaturated β-Ketophosphonates from Tertiary α-Allenic Alcohols. The Synthesis of (±)-(E)-α-Atlantone. *J Org Chem* **1996**, *61* (20), 7202–7206. https://doi.org/10.1021/jo960894z.

(16) Chemagin, A. V.; Yashin, N. V.; Grishin, Y. K.; Kuznetsova, T. S.; Zefirov, N. S. Diethyl [Nitro(Diazo)Methyl]Phosphonate: Synthesis and Reactivity towards Alkenes. *Synthesis (Stuttg)* **2010**, No. 2, 259–266. https://doi.org/10.1055/s-0029-1217103.

(17) Jacobson, H. I.; Griffin, M. J.; Preis, S.; Jensen, E. V. Phosphonic Acids. IV.1 Preparation and Reactions of β-Ketophosphonate and Enol Phosphate Esters2. *J Am Chem Soc* **1957**, *79* (10), 2608–2612. https://doi.org/10.1021/ja01567a067.

(18) Katz, T. J.; Liu, L.; Willmore, N. D.; Fox, J. M.; Rheingold, A. L.; Shi, S.; Nuckolls, C.; Rickman, B. H. An Efficient Synthesis of Functionalized Helicenes. *J Am Chem Soc* **1997**, *119* (42), 10054–10063. https://doi.org/10.1021/ja9721327.

(19) Coutouli-Argyropoulou, E.; Zachariadou, C. Synthesis of 5-Substituted Uracils and 2,4-Dimethoxypyrimidines by Wittig Olefination. *J Heterocycl Chem* **2005**, *42* (6), 1135–1142. https://doi.org/10.1002/jhet.5570420615.

(20) Thordarson, P. Determining Association Constants from Titration Experiments in Supramolecular Chemistry. *Chem Soc Rev* **2011**, *40* (3), 1305–1323. https://doi.org/10.1039/c0cs00062k.

(21) Brynn Hibbert, D.; Thordarson, P. The Death of the Job Plot, Transparency, Open Science and Online Tools, Uncertainty Estimation Methods and Other Developments in Supramolecular

Chemistry Data Analysis. *Chemical Communications* **2016**, *52* (87), 12792–12805. https://doi.org/10.1039/c6cc03888c.
